# *Aedes aegypti* from Amazon Basin Harbor High Diversity of Novel Viral Species

**DOI:** 10.3390/v12080866

**Published:** 2020-08-08

**Authors:** Geovani de Oliveira Ribeiro, Vanessa S. Morais, Fred Julio Costa Monteiro, Edcelha Soares D’Athaide Ribeiro, Marlisson Octavio da S Rego, Raimundo Nonato Picanço Souto, Fabiola Villanova, Roozbeh Tahmasebi, Philip Michael Hefford, Xutao Deng, Eric Delwart, Ester Cerdeira Sabino, Licia Natal Fernandes, Antonio Charlys da Costa, Élcio Leal

**Affiliations:** 1 Institute of Biological Sciences, Federal University of Pará, Belém, Pará, 66075-000, Brazil; geovanibiotec@gmail.com (G.d.O.R.); fevface@gmail.com (F.V.); 2 Institute of Tropical Medicine, University of São Paulo, São Paulo 05403-000, Brazil; va.morais@usp.br (V.S.M.); roozbeh@usp.br (R.T.); sabinoec@gmail.com (E.C.S.); licianatal@usp.br (L.N.F.); 3 Public Health Laboratory of Amapa-LACEN/AP, Health Surveillance Superintendence of Amapa, Macapa 68905-230, Amapa, Brazil; fredjulio@gmail.com (F.J.C.M.); edcelhamanu@hotmail.com (E.S.D.R.); farmarlisson@hotmail.com (M.O.d.S.R.); 4 Arthropoda Laboratory, Federal University of Amapa, Macapa 68905-230, Amapa, Brazil; rnpsouto@unifap.br; 5 University Hospitals Plymouth NHS Trust, Derriford Road, Crownhill, Plymouth PL6 8DH, UK; p.hefford@nhs.net; 6 Vitalant Research Institute, 270 Masonic Avenue, San Francisco, CA 94118-4417, USA; xdeng@vitalant.org (X.D.); eric.delwart@ucsf.edu (E.D.); 7 Department Laboratory Medicine, University of California San Francisco, San Francisco, CA 94143, USA

**Keywords:** microbiota, viral diversity, NGS, insect-virus, *Aedes aegypti*, Amazon, mosquito

## Abstract

Viruses are the most diverse and abundant microorganisms on earth, highly adaptive to a wide range of hosts. Viral diversity within invertebrate hosts has gained notoriety in recent years in public health as several such viruses have been of medical importance. *Aedes aegypti* serves as a vector for several viruses that have caused epidemics within the last year throughout Brazil; including Dengue, Zika and Chikungunya. This study aimed to identify new viral agents within *Aedes aegypti* mosquito in a city of the Amazonian region, where it is highly endemic. Metagenomic investigation was performed on 60 mosquito pools and viral RNA sequences present in their microbiota were characterized using genomic and phylogenetic tools. In total, we identified five putative novel virus species related to the *Sobemovirus* genus, *Iflavirus* genus and *Permutatetraviridae* family. These findings indicate a diverse taxonomy of viruses present in the mosquito microbiota of the Amazon, the region with the greatest invertebrate diversity in the world.

## 1. Introduction

Next-generation sequencing (NGS) is a revolutionary tool in molecular biology research. Courtesy of NGS, great numbers of insect microbiota have been explored, allowing the discovery of novel microorganisms, especially viruses [1,2,3]. Although the collective insect microbiota harbors many human pathogenic viruses, most viruses are non-pathogenic and have no direct public health impact. Despite this, some viruses are hypothesized to influence on mosquito susceptibility to certain arboviruses [4,5,6,7,8]. Furthermore, some phylogenetic studies indicate the evolution of pathogenic viruses from insect-specific virus to that capable of dual host tropism [1,9,10]. Therefore, metagenomic mosquito surveillance will enable new insights into the diversity and evolution of arboviruses.

The Amazon forest is considered one of the largest mosquito-related viral reservoirs in the world. Climatic and geographic conditions (frequent rainfall, year-round high temperature and dense forests) favors dissemination of several species of hematophagous diptera (mosquito, sandflies and ticks) and sylvatic animals [11]. Many mosquito-borne virus of medical importance are endemic throughout the Amazon, such as Dengue virus (DENV), Yellow Fever Virus, Oropouche virus and Mayaro virus; constituting significant morbidity for its population [12].

*Ae. aegypti* is an exotic mosquito species introduced to the Americas via slave ships coming from Africa [13]. Globally, the vector is closely associated with human habitations, demonstrating a great adaptive capacity to varied environments. The favorable climatic conditions of the Amazon have contributed to its domestication here [14]. Today, *Ae. aegypti* is responsible for transmitting a number of arboviruses such as dengue, Zika and chikungunya viruses, causing several epidemics in Brazil in the last few years [15].

Macapa in North Brazil is a typical Amazon city whereby it rains from December to May and has a dry summer from June to November. A recent study demonstrated high infestation rates of female *Ae. Aegypti* in Macapa [16]. It is well known that Dengue virus, Zika virus and Chikungunya virus are prevalent in this mosquito population. There is significant potential for the prevalence of other not-yet-characterized viruses within *Ae. Aegypti* of the Amazon and as such, may pose an emergent threat to public health. Previous studies from our group have described unusual viral sequences from *Flaviridae* and *Reoviridae* families in *Ae. aegypti* mosquitoes in the Amazon [17,18]. Using an NGS metagenomic approach, this study aimed to identify novel viruses in *Ae. aegypti* mosquito captured throughout an area of high-infestation from Macapa city, North Brazil. We found five potential novel viral species, suggestive of a rich taxonomy of arboviruses yet to be discovered in this Amazon region.

## 2. Materials and Methods

### 2.1. Mosquitoes Collection

Mosquitoes (Diptera: *Culicidae*) were collected from city of Macapá, Amapá state, North Brazil (see Figure S1), twice a month from January to March 2017. Electric manual aspirators and entomological nets were used to collect the mosquitoes. The mosquitoes were then transported to the laboratory, euthanized with ethyl acetate and morphologically identified using the dichotomous keys of Consoli and Lourenço-de-Oliveira [19] legs and wings removed. Between one and five females were grouped in pools according to their taxonomic category, place and date of collection. In total, 60 pools of mosquitoes were stored in a −80 °C freezer.

### 2.2. Sample Processing and Next Generation Sequencing (NGS)

The following metagenomics deep sequencing protocol was used. Initially, each mosquito pool was homogenized in 2 mL impact-resistant tube containing lysing matrix C (MP Biomedicals, USA) added to 900 μL of Hanks’ buffered salt solution (HBSS). The homogenized sample was centrifuged at 12,000× *g* for 10 min and approximately 300 μL of the supernatant was then filtrated through a 0.45 μm filter (Merck Millipore, Billerica, MA, USA). Next, 100 μL of cold PEG-it Virus Precipitation Solution (System Biosciences, CA, USA) was added to the obtained filtrate, mixed and incubated at 4 °C for 24 h. After, the mixture was centrifuged at 10,000× *g* for 30 min at 4 °C and supernatant discarded. The pellet rich in viral particles was treated with a mix of nuclease enzymes to digest unprotected nucleic acids. Viral nucleic acids were obtained using ZR & ZR-96 Viral DNA/RNA Kit (Zymo Research, CA, USA) according to the manufacturer’s protocol. The cDNA synthesis was conducted with AMV Reverse transcription (Promega, WI, USA). A second strand of cDNA synthesis was conducted using DNA Polymerase I Large Fragment (Promega, WI, USA). Then, DNA library was performed using Nextera XT Sample Preparation Kit (Illumina, CA, USA). The library was deep-sequenced using the HiSeq 2500 Sequencer (Illumina, CA, USA) with 126 bp ends. Bioinformatic analysis was performed according to the protocol previously described by Deng et al. [20]. The singlets and contigs were analyzed via BLAST (BLASTn and BLASTx) to look for similarity to viral sequence in GenBank’s Virus.

### 2.3. Phylogeny and Viral Annotation

Firstly, viral sequences identified in this study were used to query against NCBI protein database using the BLASTp tool to determine the closest sequences, its taxonomic classification and similarity. Secondly, based on BLAST result, the best hit sequences were download and aligned using Mafft software online [21] and phylogenetic trees were constructed using PhyML software [22] by Maximum Likelihood approach. Branch support values were assessed using the approximate likelihood ratio test (aLRT) on a Shimodaira-Hasegawa-like test. Evolutionary models and gamma distribution were selected according to the Bayesian information criterion (BIC) implemented in the jModeltest software [23]. Thirdly, the ORFs were annotated using InterProScan [24] and CD-search web using the CCD 3030 database and e-value < 0.05 [25].

## 3. Results

A total of 60 pools of *Ae. aegypti* female was collected (each pool containing between 1 and 5 specimens of mosquitoes, see the locations of sampling in the Figure S1 and characteristics of these pools in the Table S1), of which 24 were from Central and 36 were from Marabaixo and subsequently submitted to NGS protocol. Raw data were processed and after assembly the viral sequences were identified based on similarity of BLASTX comparison against to all RefSeq database in GenBank (details of viral richness in pools of pools contained viruses describe in this study were summarized in the Figures S4–S9, Supplementary Materials). We found nine virus-like sequences in two samples (AP59 and AP60) with <90% amino acid identity to different unclassified viruses, related to Sobemo-like virus, Iflavi-like virus and Permutotetra-like virus. These sequences represent five putative novel viruses, named *Aedes Sobemo-like virus, Aedes Iflavi-like virus 1, Aedes Iflavi-like virus 2, Aedes permutotetra-like virus 1* and *Aedes permutotetra-like virus 2* (Table 1). All sequences generated in this study were deposited in the GenBank (GenbBank acession: MT808014-MT808054) and they are also available in a fasta formatted file (Supplementary Materials; File S1).

### 3.1. Sobemo-Related Virus

**Figure 1:**
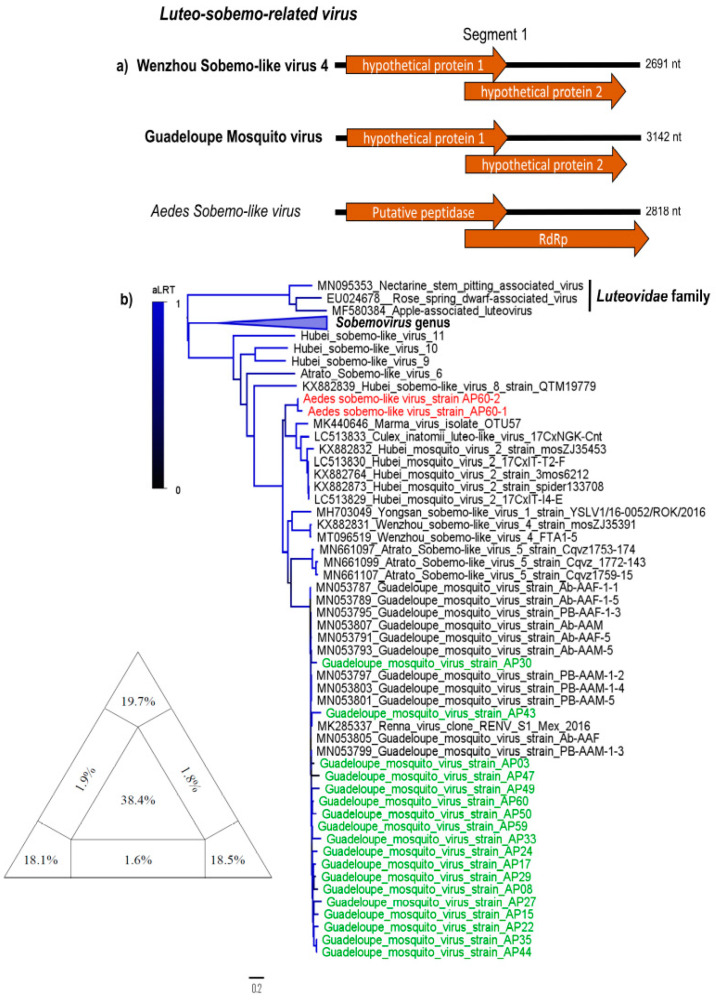
Luteo-sobemo-related viruses genomes map and phylogeny. (**a**) Open read-frame organization of closest related virus to *Aedes sobemo-like virus* and *Guadeloupe mosquito virus*. (**b**) Nucleotide maximum likelihood tree based on segment 1 for Sobemo-like viruses and *Luteovirus* and *Sobemovirus* genus. *Aedes sobemo-like virus* and *Guadeloupe mosquito virus* sequenced in this study are highlighted in orange and green, respectively. The diagram in the base of the tree is the likelihood map of the nucleotide alignment of genomes of Luteo-sobemo-related viruses. The likelihood quartet mapping is a method that allows to visualize the tree-likeness of all quartets in a single graph and provide a direct measure of the phylogenetic signal in an alignment. The triangle shows the location of all quartets calculated with the alignment used to infer the ML tree. Values in the center of the triangle represent the percentage of unresolved quartet trees (star-like trees), values in the vertices represent the percentage of fully resolved trees and values in the intermediate areas (between vertices) are the percentage of conflicting trees. The analysis was performed using GTR+ gamma correction model as is implemented in the tree puzzle software v 5.3 (http://www.tree-puzzle.de).

Two Sobemo-like virus sequences (3000 and 2768 nt) were identified in two pools, tentatively named *Aedes Sobemo-like virus* (ASLV). Sobemo-like virus belongs to an unclassified group distantly related to the International Committee on Taxonomy of viruses (ICTV) *Sobemovirus* genus and *Luteoviridae* family. Sobemo-like viruses are widely found in insects and have a particular genomic organization; many of these novel viruses have (bi) segmented genomes, different to *Sobemovirus* genus and *Luteoviridae* family viruses which are monopartite [1]. We characterized two complete segment 1s of ASLV, which possesses two open read-frame (ORFs) corresponding to a putative peptidase and RNA-dependent RNA-polymarase (RdRp), respectively (Figure 1a). The ASLV RdRp gene encodes a 450-aa protein with amino acid identity varying between 72% and 83% with hypothetical protein 2 of *Wenzhou Sobemo-like virus 4*, the closest aligned sequence in the blastx, while the capsid shares 36–54% amino acid identity with same virus (Table 1). The ASLV has a typical overlapping reading frame, −1 frameshift and a protein layout similar to that of other known sobemoviruses (Figure 1a).

In addition, eighteen viral genomes with complete coding regions similar to Guadeloupe Mosquito virus (GMV) were obtained from several analyzed pools. The viral genomes contain two segments, encoding a putative peptidase and RdRp protein on segment 1 (2400–3000 nt) (Figure 1a) and a putative capsid and one hypothetical protein encoded by segment 2 (1000–1800 nt) (data not shown). Similarly to ASLV, GMV, Wenzhou sobemo-like virus 4 and Hubei mosquito virus 2 are all currently unclassified viruses with a distant relationship to the *Luteoviridae* family and *Sobemovirus* genus [26]. Phylogenetic analysis based on segment 1 indicates that GMV Brazilian sequences are highly similar to GMV, recently detected in Guadeloupe, and Renna virus isolated from Mexico City, sharing about 100% nucleotide sequence identity, clustered into a unique clade (Figure 1b).

### 3.2. Iflavi-Related Virus

**Figure 2:**
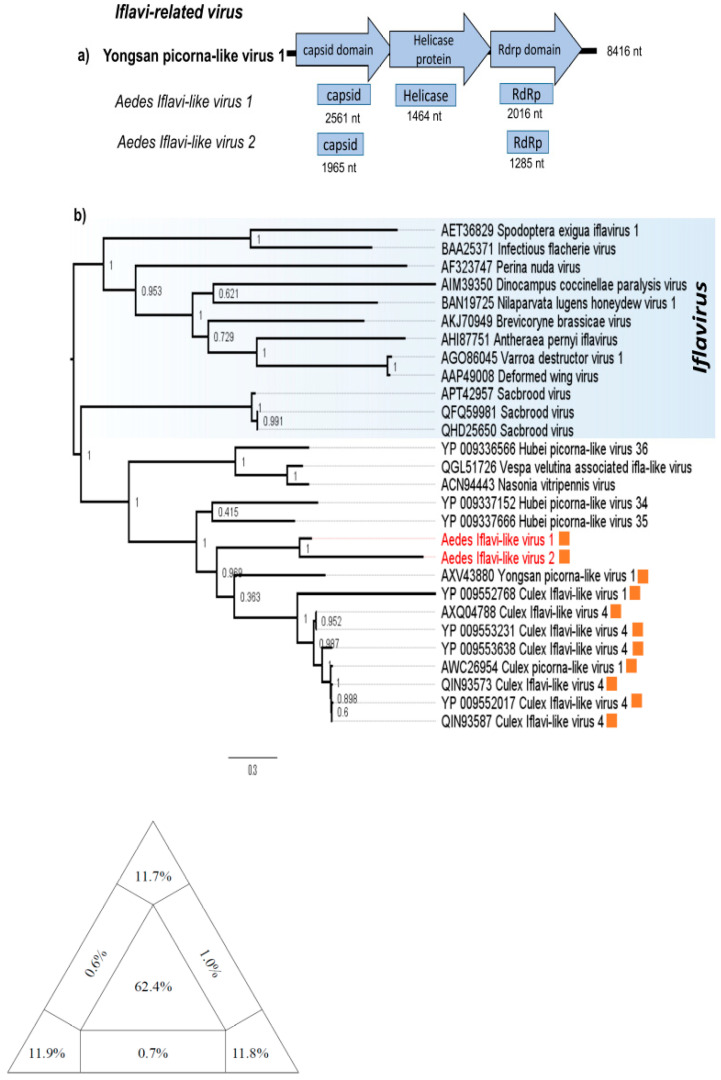
Iflavi-related contigs position in Yongsan picorna-like virus genome. (**a**) Contigs position of *Aedes Iflavi-like virus* and structure-based alignment with relative closest virus, Yongsan picorna-like virus. (**b**) Maximum likelihood tree (ML) for RdRp protein of *Aedes Iflavi-like virus* (orange) with Iflavirus genus (indicated in a blue area) and Iflavi-like sequences related to *Aedes Iflavi-like virus* by Blastp search. Sequences from the current study are colored in red. The diagram in the base of the tree is the likelihood map of the nucleotide alignment of genomes of Iflavi-related viruses. The likelihood quartet mapping is a method that allows to visualize the tree-likeness of all quartets in a single graph and provide a direct measure of the phylogenetic signal in an alignment. The triangle shows the location of all quartets calculated with the alignment used to infer the ML tree. Values in the center of the triangle represent the percentage of unresolved quartet trees (star-like trees), values in the vertices represent the percentage of fully resolved trees and values in the intermediate areas (between vertices) are the percentage of conflicting trees. The analysis was performed using JTT model as is implemented in the tree puzzle software v 5.3 (http://www.tree-puzzle.de).

One contig corresponding to the helicase gene and two contigs corresponding to the capsid and RdRp genes of two putative novel viruses; designated *Aedes Iflavi-like virus 1* (AILV 1) and *Aedes Iflavi-like virus 2* (AILV 2), were found in two mosquitoes pools (Figure 2a). All three fragments have low amino acid identity (<56%) with *Yongsan picorna-like virus 1*, the best hit with blastx (Table 1). This low identity is reflected in the topology of RdRp-based phylogeny, with AILV 1 and AILV 2 grouped in a separated clade from other Iflavi-like viruses (Figure 2b). The similar topologies were observed in the helicase and capsid-based ML phylogeny (Supplementary Materials). The phylogeny of best hits on blastx and Iflavirus members show diversity of Iflavi-like viruses, with all Iflavi-like and Iflavirus members previously isolated from arthropods, mostly insects [27]. AILV strains grouped into a cluster which shares a common ancestor with other viruses originally described in mosquitoes, dismembered from other insect-viruses (Figure 2b), however, only Yongsan picorna-like virus 1, AILV 1 and AILV 2 have been found in *Aedes* mosquitoes (unpublished). Since we used NGS to amplify viral sequences it is possible that contigs of ALV1 were derived from distinct viral genomes, likewise contigs of ALV2 also may have been amplified from distinct genomes. Nevertheless the phylogenetic analysis showed ALV1 and ALV2 are not the same virus and they likely represent new species.

### 3.3. Permutotetra-Like Virus

**Figure 3:**
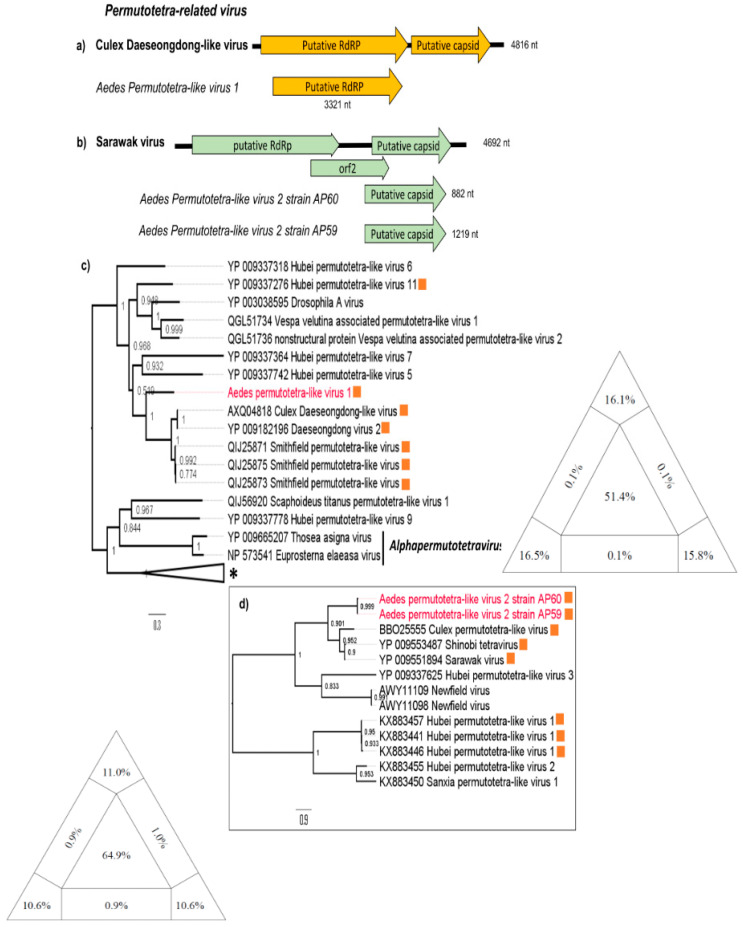
Permutotetra-related contigs position in most related viruses genomes. (**a**) Schematic representation of Culex Daeseongdong-like virus, *Aedes permutetra-like virus 1*, (**b**) Sarawak virus and *Aedes permutetra-like virus 2*. (**c**) Maximum likelihood phylogenetic tree for RdRp protein and (**d**) maximum likelihood phylogenetic tree for capsid protein. Viruses originally found in mosquitoes are marked by orange. Sequences from this study are indicated in red. * Indicate the location in the RdRp tree of the reference viruses used to construct the capsid tree. The diagram in the base of the tree is the likelihood map. The likelihood quartet mapping is a method that allows to visualize the tree-likeness of all quartets in a single graph and provide a direct measure of the phylogenetic signal in an alignment. The triangle shows the location of all quartets calculated with the alignment used to infer the ML tree. Values in the center of the triangle represent the percentage of unresolved quartet trees (star-like trees), values in the vertices represent the percentage of fully resolved trees and values in the intermediate areas (between vertices) are the percentage of conflicting trees. The analysis was performed using JTT+gamma correction model as is implemented in the tree puzzle software v 5.3 (http://www.tree-puzzle.de).

We found three partial genomic segments from two putative novel Permutotetra-like viruses (Figure 3). A RdRp sequence (3321 nt) presented 53% of amino acid identity with Culex Daeseongdong-like virus, the most similar virus. This putative novel virus was named *Aedes permutotetra-like virus 1* (APLV1) (Figure 3a). Another two capsid sequences (882 and 1219 nt) belonging to this group shared ~48% amino acid identity with the most similar virus, Sarawak virus. This putative novel virus was named *Aedes permutotetra-like virus 2* (APLV2) (Figure 3b). The cluster formed by APLV-1, Culex Daeseongdong-like, Daeseongdong virus 2 and Smothfield permutotetra-like virus have been found in mosquitoes (Figure 3c) [3,28]; similarly, the clade formed by APLV-2, Culex permutotetra virus, Shinobi tetravirus and Sarawak virus have also been detected in different mosquito species (Figure 3d) [29,30,31].

## 4. Discussion

In this study, we analyzed 60 pools of *Ae. aegypti*, and, as expected, we found several highly divergent sequences, which possibly represent novel viral species. These viruses belong to the Luteo-sobemo-related virus, *Iflavirus* and *Alphapermutotetravirus* genus.

One novel Luteo-sobemo related virus was found in two *Ae. aegypti* samples, named *Aedes sobemo-like virus* (ASLV). The sobemo-like viruses are (+) ssRNA unclassified viruses distantly related to the *Sobemovirus* genus and *Luteoviridae* family (Figure 1b), which infects plants and is known to be vectored by arthropods. Although viruses belonging to *Sobemovirus* genus and *Luteoviridae* family are known plant viruses and are of monopartite genome, Sobemo-like virus members have bi-segmented genomes and have been isolated primarily from insects [1,26]. It has been speculated that this group of viruses should be proposed as a new family [32].

Iflavirus members are a new recognized family called *Iflaviridae* (order *Picornavirales*), under the *Iflavirus* genus. All Iflavirus members are insect-infecting viruses and they have been identified in a wide range of hosts belonging to the class *Insecta*, although plant-infecting Iflavirus-like virus has been reported from tomato (*Solanum lycopersicum*) [33]. Currently, there are fifteen species in the genus *Iflavirus* recognized in the last report of ICTV [34]. However, sequence identity at the amino acid level of the capsid proteins above 90% is used for species demarcation criteria for the *Iflavirus* genus and several tentative novel viruses have been identified showing sequence similarity to members of the genus Iflavirus and yet are classified as iflavi-like viruses. Through NGS analysis, we assembled five contigs that showed similarity to Iflavi-like viruses, here named *Aedes Iflavi-like virus 1* (AILV1) and *Aedes Iflavi-like virus 2* (AILV2). For this reason, there is a possibility of these contigs belong to distinct viral genomes. Our analysis shows that ALV1 and ALV2 share a common ancestor that diverge from each other and likely represent two new viral species. BLASTp searches showed that both AILV1 and AILV2 shared low sequence identity (less than 90%) with other members of *Iflaviridae* at the amino acid level (Table 1), indicating that both are novel species of *Iflaviridae* family [34]. Additionally, sequence analysis showed that the AILV1 and AILV2 shared 50% capsid amino acid sequence identity (data not shown), suggesting that they are members of the different species. The phylogenetic analysis showed that AILV1 and AILV2 form a well-supported clade, suggesting the representation of a novel clade within the *Iflaviridae* family. According to ICTV, “The *Iflaviridae* family is expanding rapidly and will likely undergo revision in the near future” and possibly new species and genus will be included in the official taxonomy.

Additionally, we also detected three partial genomes of two putative novel viruses (APLV 1 and APLV2) closely related to unclassified permutotetra-like viruses. *Permutotetraviridae* is a recent classified family with a single genus (*Alphapermutotetravirus*) and two prototype species (*Euprosterna elaeasa virus* and *Thosea asigna virus*), restricted mainly to insects in the order Lepidoptera (butterflies and moths). In recent years, a wide range of highly divergent viruses distantly related to *Permutotetraviridae* family has been identified [1,3,30,35]. The lack of common genomic organizations in permutotetra-like virus members and the formation of two large and well-supported clades (Figure 3) support the need to create new groups for the current unclassified viruses of this family. So, the permutotetra-like viruses (APLV1 and APLV2) found in this study may represent new species within different genus/family. Both viruses are grouped with viruses isolated only from mosquitoes, indicating a likely common origin within their respective clades (Figure 3c,d).

All novel viruses reported here share a common ancestor with other viruses originally described in mosquitoes, dismembered from other insect-viruses, suggesting a close evolution with their mosquito hosts. Recent phylogenetic studies in several RNA insect-virus families have indicated that they are ancient agents with highly distinct lineages, leading to the credence of probable co-evolution and expansion with their insect hosts [10,36,37]. The hypothesis that insect-viruses have been closely associated with their insect hosts for a long period of time is supported by studies that demonstrate vertical/transovarial transmission (TOT), whereas some become integrated into the genomes of their own arthropod hosts [38,39,40]. Another possibility regarding insect-virus evolution is of host association, whereby dual host viruses evolved from insect-specific progenitors, with many arthropod-borne viruses possibly emerging to vertebrates and plants in this way, including complete adaptation to vertebrate or plant hosts and thereby losing the need for an invertebrate host [2,41]. Importantly, none of these novel viruses are closely related to known vector-borne pathogens of humans or other mammals. The alignments used to construct maximum likelihood trees (ML) have a poor phylogenetic signal, as determined by the high proportion of star-like trees in the likelihood mapping analysis. Although the low quality of alignments has little effect on the topology of trees constructed with other methods, it may have a significant outcome to estimates mutation rates or to the measurement of divergent-time. Consequently, in the future with the identification of new viral sequences the interpretation of data may also be affected.

The diversity of arboviruses remains to be explored, especially in the Amazon, known for being a rich bioma with many viral species. For example, the Amazon region is identified as the starting point for transmission of the yellow fever virus in a recent outbreak in Brazil that killed almost 700 people between December 2016 and March 2018 and the Amazon rainforest functions as a ‘reservoir’ region for several arboviruses [42]. Other studies have been identified novel virus presents in the mosquito microbiota in this region, in the host *Aedes aegypti* and anophelines [17,18,43], reinforcing the idea that our current knowledge about the diversity of viruses is still very limited. Furthermore, insect-specific virus (ISV) compose the majority of mosquito virome the virus-virus interactions may affect the transmission of some viral pathogens [44,45]. From description and characterization of these viral agents, we can gain knowledge useful to some biotechnological strategies in combating epidemic viruses, such as Dengue, Zika and Chikungunya.

Recent massive metagenomics studies have expanded our knowledge about diversity of a great number of invertebrate viruses, which include many unclassified groups, inclusive of luteo-sobemo-like virus, Ifla-like virus, permutotetra-like virus, among others [1,2,3,46]. The ICTV officialization of these taxonomic proposals requires time, alongside large quantities of sequence analysis and therefore, the identification of novel viral sequence within this study greatly contributes to the correct taxonomic classification of these “virus-like” sequences. Ultimately, our results highlight the importance of identifying and characterizing novel viruses to expand our understanding of the taxonomic diversity of viral groups (families, genera and species), which is currently poor. The host range influence and vector biology for these viruses, alongside the ecological and evolutionary history of *Aedes aegypti* microbiota, the principle arboviral vectors of Brazil, need to be further studied.

## Figures and Tables

**Table 1:** Amino acid similarity of sequences identified in metagenomics analysis from *Ae. aegypti* pools.

Virus Name	Closely Related Viruses ^1^	Gene	Length (nt)	Cover ^1^	Amino Acid Identity ^1^
Aedes Sobemo-like virus strain AP60-1 *	Wenzhou Sobemo-like virus 4	Peptidase	1719	42%	36%
		RdRp	1308	88%	72%
Aedes Sobemo-like virus strain AP60-2 *	Wenzhou Sobemo-like virus 4	Peptidase	2768	99%	54%
		RdRp	1308	100%	83%
*Aedes Iflavi-like virus 1*	Yongsan picorna-like virus 1	Capsid	2561	60%	49%
		Helicase	1464	100%	46%
		RdRp	2016	97%	48%
*Aedes Iflavi-like virus 2*	Yongsan picorna-like virus 1	Capsid	1965	49%	47%
		RdRp	1285	50%	52%
*Aedes permutotetra-like virus 1*	Culex Daeseongdong-like virus	RdRp	3321	92%	53%
Aedes permutotetra-like virus 2 strain AP59 *	Sarawak virus	Capsid	1219	91%	48%
Aedes permutotetra-like virus 2 strain AP60 *	Sarawak virus	Capsid	882	94%	49%

^1^ Based on Blastp. * Same virus (>90% amino acid identity between them).

## Supplementary Materials

The following are available online at https://www.mdpi.com/1999-4915/12/8/866/s1, File 1: Text containing all contigs in fasta format; File 2: Figure S1. Map showing the coordinates where mosquitoes were captures in Brazil; Figure S2. Phylogenetic tree of Iflavi-like virus and Iflavirus genus members based on the alignment of capsid region protein; Figure S3. Phylogenetic tree of Iflavi-like virus and Iflavirus genus members based on the alignment of helicase region protein. Figures S4–S8. Virome composition of pools of Aedes mosquitoes. Figure S9. Comparison of viral richness of pool AP59 of Aedes mosquitoes. Table S1. Characteristics of pool samples of Aedes mosquitoes from Macapa.
