# Supplementary material for: Aedes aegypti from Amazon Basin Harbor High Diversity of Novel Viral Species
Source: Viruses. 2020 Aug 8;12(8):866. doi: 10.3390/v12080866 (PMC7472207; doi:10.3390/v12080866)
Supplement: Supplementary file 1 [file viruses-12-00866-s001.zip › viruses-862891-supplementary/viruses-862891supplementaryFile1forXML.pdf]

> Aedes_sombemo-like_virus_strain_AP60-1_MT808055
GAGTTTCTGAGTTATCTTCTCCGGATCTCTGCAAACTGCATAATCTCTAAATGTCGTTAACCC
TTTTAACAAGACGATGGTTATACTAGCAAACCTCCTGCTCTTTACGGTATTTGTGAAACGGA
TTGCATCGTGGACACAAGACACTATGATATCCATAGTGTCTTGTGTCCACGATACAATCCGTT
TCACAAATACCGTAAAGAGCAGGAGGTTTGCTAGTATAACCATCGTCTTGTTAAAAGGGTTT
AACGACATTTGGGAGATGTTATTACCAAAGCTATCATTATGGTCCCTGCTCTTTACGGTATTT
GTGAAACGGATTGTATCGTGGACACAAGACACTATGGATATCATAGTGTCTTGTGTCCACGA
TACAATCCGTTTCACAAATACCGTAAAGAGCAGGAGGTTTGCTAGTATAACCATCGTCTTGT
TAAAAGGGTTAACGACATTTAGAGATGTTATTACCAAAGCTATCATTATGGTCACCTGTAGAT
GGATGAGCCAATCTCCTTGGAAGAATTCTTGCACCTTTCCAGGCGCCTCACTCGATTCCATG
GTTAGCATGAGTGAAAGGAGTAACTTACCATGTCGTGGGCCAGTGTTTCTGGGTAGATGGG
GCCTGGTTACAGCCGCTCACGTCATAGAAGGGTTTGAGTATCTTAGCATTTACCGGGACGAT
CAACGCCGGATTGAGGTCAGTTCCGATGTGTTTGCTATAGGACATGGAGATTATGCAGTTTG
CAGAGATCCGGAGAAGATAACTCAGAAACTCGGCTTATCTAAAGCCAAACTTTCGAGATTA
GCAGTAAGTAAAGACAGTGGTCTTAGTGTAAACGTCACGGCCAACCATCAAAGAACCATTG
GGTTCTTGGAACAGCATGCCCAATTTGGGTTTGTTAAGTATACAGGCAGCACCGCTAAAGG
TTTTAGTGGAGCGCCGTATCATTTTGGCAGGACGGTCTTTGGAATGCACTTGGGCGCTGATG
CCTATAATATGGGTTACGATGCTGCTTTCCTCAAGTCAGAGCTGAAGCCGAGTCGTGTGATT
AAAGATATAACAGGAATGAGAGGCGAGGATAGTATAGATTGGCTGTATGACCAATTGGAAA
GATATGATGCTTTTCTGACCTCCCTGAGGCCGTATAACCCAGTGATGTCAGAAAGGAATTGG
CAAAGGAGAAAGACCCCGTTGTGGCTATGGACGAAAGGGTTGTTGAACGAGTGGGGTAGA
ACATCAATCTACCAGAATGAGTCGTTAGGCGTTGAGGATTTACCCCTAGCACCACGGAATGC
TATGAGCTTTAAGGATCAGGGAAACTTGATCAGGGCTCCAGCTGCCGTTGCTGGGGCCCAT
GGTATGGAGAATGTCCAGGAAGCTGCTCCGAGCCAAAACGGGCAAACATTGCCCCAAATG
GCTTGCACATCCCAGACACCTTCGGTTCCTTACCATATGGAATCCCTTTCGTCGACGCTTGC
TCAGCCAAACGTAGTTTCCGCGCGCACTGCAAGAAACAGGCAGAGACGGTTAAACCGCCA
ACGGCAGAGGAACGAACTCGCGCTGTACAAACAGCAATACGGTCCTATGCCCCATGGCGAT
ATGACCTCACCTCCGCCGCAGATCCCCATAGCTGGTTCGATTGTGAATTCAACAAGGCCTTT
AATGGAGATAGATTTAAACAGTACCCCAGGGTATTGCAGTCTTACTCTCTTAGGCTCTACGA
ACAAGCAGATTTTAGGATGGGATGGGATCAATTTTGATCCCGATCGAGTTACCATAGTCAGG
CAGCATGTGAAGTTCCGTTTCGATAAGCTCCTTGAGGGGATACCAGTTTACGATGACCTTAA
GGTTTTCGTTAAACAGGAACCCCACAAGTTGAAGAAACTCGAGGATGGGATCCTATCGCCA
GATCTATCAAGACAGCTTCACCGCCCCGTTCACCGACAGCGATTTTGTATAGCTTATCTACCC
CCCGCTCTACTGGCGTCAGTAGGACAGACTCCGTGTTTAGTCGGATGGAGTCCAGTACGAG
GAGGGTGGAGAGCTATACAAAATCGCTTCGGTAACGGGGCGGTGAACTGTCTTGATAGATC
TGCATGGGATTGGACGGTACAGGGATACTTGGTAGATCTGTGGATTTTGTTTCTTGAAAATT
TGCCAGTCAATCCTCCTGAGTGGTGGCTAGCCATGATGAAACTTCGGTTCAAGCTACTATTT
GAGGATGCTTGGTTCAAGTTCGAGGATGGAACAAGAGTGAAACAAGGGACTAGAGGTGTA
ATGAAGAGTGGGTGTTACCTAACTATTTTGTTGAATAGTTTGAGTAACAAGCACTGCTTCAT
TAGATACCACTGCCTGGACCGTGTCATCACCTATTCCATAGGTGATGACACTGGTCCAGGAG
GCCTTATGACATGGTCTAGCCGGCATTATTGGCTACCGGATTATGTCCGCCAATTGGAAATTT
TGGGAGTCACGGTCAAGGGTGCCAAAGTTCAGCACTGGGTAGAATTTGCCGGCTTTTGTTT
TGATGGAAAAACTTGTTATCCGTCGTACTGGCAGAAACACCTGTTTAATCTTGCGCATACGC
AGCGTCTTGGGGAGACGCTAGCTAGTTATCAATATTTGTACGTCAATGAGCCCGTGATGTAC
GAATTTCTCTGCAGAGTGGCTCGTGAATTAGGTCCTCAGTATGTTTTGCCTAAGATGGAAGC
ACTAGACATAATGAACCAGCCTGTTTGGGTTGGAATATGTCAAGTTCCCTTTCGTCTTGAGG
CAATTCCTATTGAGATGAGTTGGCTATTTATGATTTCCAGCCTGTTTAGGTTGGAATATGTCA
AGTTCCCTTTCGTCATGGAGGCATTCCTATTGATGACTAGAAAGGGAACTTGGACATATTCC

AACCTAGACAGGCTGGTKYWYRTGTACGTCAATGAGCCCGTGATGTACGAATTTCTCTGCA
GAGTGGCTCGTGAATTAGGTCCTCAGTATGTCTAGCCT

>Aedes_sombemo-like_virus_strain_AP60-2_MT808056
AAAAAAAAAATCTATCTACAGGTGACCATAATGATAGCTTTGGTAATAACATCTCTAAATGTC
GTTAACCCTTTTAACAAGACGATGGTTATACTAGCAAACCTCCTGCTCTTTACGGTATTTGTG
AAACGGATTGTATCGTGGACACAAGACACTATGATATCCATAGTGTATTGGACTTATAGAAA
GGCACAACGATGCTATGTGTTTCTCTCGACCTACCAATCCACTGGAAAGTGGCCTATTTGGT
TGGACACTGAGAAAATCATGGCGTTGACTATTTGTGCTTTTATAATTGTCATTTTCACGTACA
AGGCTGTGCAGTACTATAGAAGCAGGAGGATTAAGATAGTCTGGCCAACGGAAACAAAAA
CAGGCGGATATGGCAGTGAGAAAGCCATGTGTGGCAGTTCCTTTGATATCGCCAAAGTCTT
GCCTTCGTTCCAGGCTACAGTGTTAGCTTCTCTTGATGGAGTAACTTACCATGTCGTGGGCC
AGTGTTTCTGGGTAGATGAGGGCCTGGTTACAGCCGCTCACGTCATAGAAGGGTTTGAGTA
TCTTAGCATTTACCGGGACGATCAACGCCGGATTGAGGTCAGTTCCGATGTGTTTGGCATCG
GAAATGGAGATTATGCAGTATGCCGCCATCCGGAGAAGATAACTCAGAAACTCGGCTTATCT
AAAGCCAAACTTTCGAGATTAGCAGTAAGTAAAGACAGTGGTCTTAGTGTAAACGTCACGG
CCAACCATCAAAGAACCATTGGGTTCTTGGAACAGCATGCCCAATTTGGGTTTGTTAAGTAT
ACAGGCAGCACCGCTAAAGGTTTTAGTGGAGCGCCGTATCATTTTGGCAGGACGGTCTTTG
GAATGCACTTGGGCGCTGATGCCTATAATATGGGTTACGATGCTGCTTTCCTCAAGTCAGAG
CTGAAGCCGAGTCGTGTGATTAAAGATATAACAGGAATGAGAGGCGAGGATAGTATAGATT
GGCTGTATGACCAATTGGAAAGATATGATGATTTGGAATACTCCCGTTCACCGTATAACCCTG
ACGAGTACAGAGTCCGGATTGGAGGTAATTATCATATTGTTGATGAAGAAGTTTTGAGTAGT
TTGCTTCAAAGGCGAGCTGGCAAGAAAGCGTCTCAGGATATTGATTATCTCCCAGAAACAG
CCTTTTCTGACGGACGTGAGTCAGTAGACTCTAGTGATGTCAGAAAGGAATTGGCAAAGGA
GAAAGACCCCGTTGTGGCTATGACGAAAGGGTTGTTGAACGAGTGGGGTAGAACATCAAT
CTACCAGAATGAGTCGTTAGGCGTTGAGGATTTACCTTTAGCGCCTAGGAATGCCATGACGT
TTAATGATTCGGAAAACCTGATCAGGGCTCCAGCTGTAGTTGTTGGGGCCCGTGGCATGGA
GGAATATCAGAGGGAAAGTGTACAGAGCCACGACTTGCCAAGATGCAAGAAAATGGATTG
CATGTGCCAGAAACCTTTGGAGACCTACCATATGGAATCCCTTTCGTCGACGCTTGCTCAGC
CAAACGTAGTTTCCGCGCGCACTGCAAGAAACAGGCAGAGACGGTTAAACCGCCAACGGC
AGAGGAACGAACTCGCGCTGTACAAACAGCAATACGGTCCTATGCCCCATGGCGATATGAC
CTCACCTCCGCCGCAGATCCCCATAGCTGGTTCGATTGTGAATTCAACAAGGCCTTAAACGA
GATAGAGTTAAACAGTACCCCAGGGTATTGTAGTCTTATTCTTTTAGGCTCTACGAACAAGC
AGATTTTAGGATGGGATGGGATCAATTTTGATCCCGATCGAGTTACCATAGTCAGGCAGCAT
GTGAAGTTCCGTTTCGATAAGCTCCTTGAGGGGATACCAGTTTACGATGACCTTAAGGTTTT
TGTTAAACAGGAACCCCACAAGTTGAAGAAACTCGAGGATGGGACCTATCGCCTGATAAGT
GCTGTCAGCCTGATAGATACTTTCATAGACAGAATTTTGTTCGCTTGGTTAGCCCGAGCTCA
ACTGGCTTCAGTAGGACAGACTCCGTGTTTAGTCGGATGGAGCCCAGTACGAGGAGGGTG
GAGAGCTATACAAAATCGCTTCGGTAACGGGGCGGTGAACTGTCTTGATAGATCTGCATGG
GATTGGACGGTACAGGGATACTTGGTAGATCTGTGGATTTTGTTTCTTGAAAATTTGCCAGT
CAATCCTCCTGAGTGGTGGCTAGCCATGATGAAACTTCGGTTTAAGCTACTCTTTGAGGATG
CTTGGTTCAAGTTCGAGGATGGAACAAGAGTGAAACAAGGGACTAGAGGTGTAATGAAGA
GTGGGTGTTACCTAACTATTTTGTTGAATAGTTTGAGCCAAAGCCTGCTCCATTACATAGCA
AATGCCCGTTGCGGACAACCCATGGCTAGGAAACAACCCAATTCCATAGGTGATGACACGG
TCCAGGAGGCCTTAGAATGGCTACCGGATTATGTCCGCCAATTGGAAATTTTGGGAGTCACG
GTCAAGGGTGCCAAAGTTCAGCACTGGGTAGAATTTGCCGGCTTTTGTTTTGATGGAAAAA
CTTGTTATCCGTCGTACTGGCAGAAACACCTGTTTAATCTTGCGCATACGCAGCGTCTTGGG
GAGACGCTAGCTAGTTATCAATATTTGTACGTCAATGAGCCCGTGATGTACGAATTTCTCTGC
AGAGTGGCTCGTGAATTAGGTCCTCAGTATGTTTTGCCTAAGATGGAAGCACTAGACATA

>Aedes_Iflavi-like_virus_1_capsid_MT808057
TTTTTCGTTCTTGTTCTGTTGTTGAAAACGTTCTTTGTGTGTTTGAAGATTGAGATAGAATAA
CACTAGTGTATAAGAGACAAAAAAGTAGTTTCAAAGAGAACAACAAGAAAGAGACGCTAA
CTATAGAATGTATATTTTTAATCAAATACATTTGATCGTTAGCGTCTCTTTCTTGTTTTGTTTTT
TGAACCTATTGTATTCTATATTCAAACGCAGTTATAGTATAACTACGGTAGAAGAGACAATAC
AAGAGTTTCAAAGAGAACAACAAGAAAGAGACGCTAACGATCAAATGTATTTGATTAACAA
TATACAGTTGATAAATTGGACAAGAGTATGGAGGTACTTAAGAAGGCGGTTGTCTAGGAGC
CGCCTCCATACTCTCTATACTAGTGGTGTCCGTAATTATCTTGCTTCCAATCGCTTTTATGCGT
ATAGGCAGACGGAGAGCAACTATGGAGGAACCTACTGCATCACCATCACCTTGCGTACCCG
AGACGGCTCACGACAACTTCTTTTTGTAAGAAGAGAAGGGAATTCGAAACGTAATATCTTT
GAGGATGGGTACAAAGAGGTATTTCAGTATCTGATGAGTTGGAAGCCACAAGGATTGGATA
GTAATCAACAGACCCCACTGGCTATTAGTACAGATGAAAATGTTGAGGTGTCAGATAAAAA
GGAAAATGTTATTATTGAAGGAGCAGAAACCCGAGAGACTAAAGAAGCTAAAGTGAACTT
AGATCACAATTTAATTAATTGGTCAACCACAGAAGGCACTTATGACTTCACAGAAGGGGAG
GGATCTATTATGGCTAGGTGGATGCCTCTTAGTAGTGTAGAGGTGAACCAGACTATTGCTACT
AGAGCCCCAATTTGGCAAGCAAAAATAATAGAAGACATAGCAGCGTTGAAGAATATTAATG
TACTTCCAATGCGTGGATTTATGCTTGGAAAATTTGATGTGGAGTTTAAATTGGTTTTACAAG
CCACTCCTTTCCAGGCTTGTAGTCTGTTGCTATCATGGTGTCCCCATCCATATGGGTTGATGC
AGTACAATGCCATGGTTGGAACAGAGGCATTGGAGGCTACTAAATTGAATCAAAGACTTCC
AAAGTATGTACCATTTCGAGATTATTTAGATGTTACAACTGCCGTACAGCGACCAAACGTTAT
TTTGGATGTGGCTTCAGGAGGAGAAGCTGTGTTGCGAGTGGGCCAAAAATTTCACCGTACA
TTCATTCGCAACTTTGATTTTGACCGAGTCACTGGAGTGAATATGGGCATTCGAGGGGGATA
TCAAGGATTGTTGTTGTTACATCAGTTGACTCCAGTGATGGTTGGTAACGGAACACAATCAT
CATTTACTGCACGCATTTTCTATCGTTTTGTAGAAGCAAAATTGACTGCAATGACTCAACCA
GTGGAAGCCCGGACTAAAGTGGAATTACCATACATGTATGTAATTCCAAAAAATGAAGACG
CAAAATTTACCGAGATGAGTTCAGTAGCTGCCGAAGAGCAAAAGAGAACGATACTACGTAC
TAAATAGCGAACGCAAGGGCCGGCTATGGGTGCACTTTCCTTGATAAGGGGAGTAGGAATG
ACAGCTACAGGATTAATAGCTTCAGTAGAAGCTGTCTCTATGATACGAGGCACGAGACCAC
GAAATCGAGATAAACCCAATGATATAGTAGGACAAGTTGTTGTTTCTCCCCGACCTAGACCA
AATTTTACCAATGGGGAAGGAATTGATGGAGCTGCTGTTATTGGATTGAGTTTTTCGGAACT
GACAAATGTTTTGGAAAGTTTTAGTGATGAACCAACATCGTATAAGGATTTCGTTAACATAA
ATGGTTTGCTCACAACATTTGAGTGGAACACAGGTGATGCTGTGGGTTCAAACTTATGGGT
GTGGAATGGACATCCAACGGATTCTGGGAAGGCACTCACAGAAGACCAAATTGCCACAGG
AGGTTTTTCCGTTAATCAATTATTTACGCCCTTGTCAATTGCATCATCCGGTTTTACTAATTAT
GCAGGTACAATAAAATTGTATTTTCAATTTGTAAAAACAATATTCCATAAAGGAGCTGTGGA
GGTTTCAATCCAGTACGGACGGGAGCTAGTGGATAATGCTCTGGAGAATTCGTATGTAAAAG
TTATTAATTTGCAAGATTGTGCTGGGTTCGAAATGGTTTTCCCATATATTTATGACACACCGAT
GCGGTCAATTACGGGAACATCTACTCCAAGCTTTTTACCTTCAGTGGCTACTTCAGATATATC
GGCAATGGTCCACAGTGCCAGAATAACAGTGCGCGTCCTTAACCGTTTAGTGGCACCGGAT
ACAGTTGCGAACTATGTCCAAATGTTAGTTTGGATGAAAGGAGGAGAGGATTTCGTCTTGA
GTTTTCCTAGGCCATATAACACCACACTCGCGTATCCCACTGGCAATGGATTTTATCCCCCAC
AAATTATTCCATATAGTGTTGGCGCCACAGTACCAGAATAACAGTGCGCGTCCTTAACCGTT
TAGTGGCACCGGATACAGTTGCGAA

>Aedes_Iflavi-like_virus_1_helicase_MT808058
AGAAGTTAAGTCTATTGCAGTGCAAGAGATACATGATATGGTTGGGCGACTATTCCCCAAAA
TGACCCAAGTTAAGGAAGTATCATCATCAGTCTGGCAAATAGCGCAACACTTGGTGCATGT
GGCACTGGCAAAACAATGGAAAACGGCGGCGTGGGCTAGCTTTTGTATTATGCAAGAGCTA

GACCTAATAAAGTTGGCGCAATGGAATCGAGTAAAGGAACCCTTATTGGCTCTATTTCAGCA
GACAGAACAATTCCACACTCAGGGACCAGATATGGGTACTTGGACTGCGTTAATGCAAGTG
TTAACAGCCGTGGTTTGCGCTAGATTGCAGGTGAAGGCAGACCACGGAATAAGGGCCCATC
TATTGAAAATATTTTCCTCAAACGATTATAGGTCAGTGACTGGCATTAACTCTGTACTTCTGT
TGGTACGTAGTATAATGACATCTGTGTCTACTTTGGTGACGTGGGCAACTAAGGAGAGAGAT
CCCAATATAGAGCTCCTGCAATCGCTACAGAATCAAGGTGGAGAGTTGTATGAATTTGTTCA
GGATGCTAATGTGTTCCTTACACACTTCTCAGAGTGTGACTTTGCCAAGCGAGAGGGTCGTA
TTCGACATTTGGCAACTATATTGCGAGCGGTTAAAATAAGAGAGATGCTGTTACGAATTGGA
CAACCCAATGTAACTGGACCTGTTTTGTCTTTGTGTACAAAGGTAATAGACAAGGCTGCTAA
ATGTCAATATTTGATGAAATGTGACGTAGTTAAAATGGAACCATATGTGTTTTATTTGTCGGG
AGCACCTAAATGTGGGAAAAGTTTTCTAATGTATGATATAGTTCCCCATTTGTTTAAGAGTAT
TGGGATAAATCCTCCAACAGTTGACTTCATGTATACAGTTACTGCTGGCGTGGATTATTGTAA
TGGATTGAGGTCTGATCACTTGGCTTGGTTGTTTGATGACGCCTGGAATTTGCTTGATGAGG
AATCTTTCCGTAAATTGGCACAGTTCTTCTATGGCTTGGTAACTTCAGCACCATTTAATGCTC
CCCGAGCTGAATTGGAGCATAAAGAACAATTGGCCCAACCTGTAGTAGTGGCCGTGGCTAC
TAACAATGCATTTCCCCGCCATAACATGATGGCGTGCCATTTGGCCATATGGAGGCGGAGGA
ATTGCTTGTGGTATGTGCAAACTAAAGGGAATCGACCCATTAGTGAATTCTCTAAGGAACAG
TTGGCCGCATTTGACCACGTGGAATTCTACCGGTATGGGAATGTTTGTGATGAAAACTCTCG
GTTAGAACAAGTGTATACATACACAGAGTTTCTGCAGTGGTTAACAGAGGATTTTAAGAAA
CATCATGAACAGGAAAAGGGCAATCAACAAAGCAAATATCGTAGCTTGACAAAGTGCTTGA
GTGGGAGCGCATTAGATGATTTGAGTTTGGAGAACCCATT

>Aedes_Iflavi-like_virus_1_Rdrp_MT808059
AGGTATGAGTTTGTTGGAATTGGAAGGTGACCCTTATGTGGCGGAAGATTTCCAGGCCCAA
GATGACTGTGTGTATGATGTGGATATGCGAATTGAAGCAGTGATGAAGCACAGCACCGCATT
TGATGATGATAACGTGGAACATGAAAATGAGAATGGAGTGATGTATAATTATCAAGCACCAG
GAATGTGTGGAAGTTTGGTGTGCATTAATTCTAATCAACCCATTGTATCGATGCACATAAGTG
GTAGTGCTGCACAAAATAAAGGGATTGGATGTATATTATATCAGGATGACTTTGAAACTCAA
GGTATGAGTTTGTTGGAATTGGAAGGTGACCCTTATGTGGCGGAAGATTTCCAGGCGCAAG
TGATTGGATTTGTAGACCGAGATCAAAGCGTTTTCATACCAAATAAAACAACGATTAGACCT
TCATTAATAGATAAATTTTATACTGAACCTACAAAAACTATTCCAGCATTTTTATCTAAGGATA
ATCCAGGATACCCACATCAGTTTGGTCCACTACATTATGGGGTTTTAAAAAATGGCAAACCA
AGCGCAGATTTTCCCAGCCGATTGGTTGAGAAAGCATACAGTTTTGTTCGGTCTCGTGTGTT
ATCAGGGGAAAAAGGACCATTCCCCTACCGCATATTAACTATGGAAGAGAGTATAATGGGGT
ATGGTATTCGTTATGAAGGAATGGACCCAGAAGAGGATGCATACATGGGGATGATGGAACTA
GATACAAGTGCAGGGTGGCCCTATTCCACAGTGGCAATCAGAAAGAAATTTGGAATTACAG
AGCTCAATAAGAAGCAGTGGATTAAGGTTGAGTTTGGAGAAAACCAGTTACCACAGCAATG
TTGGGTGCATGGTTATGTTAGAGAACAACATGAAAGTAATATGGAGAAGCGTGAACGAGGT
TTATCTGTCTTTAATGTGTTCCAAGACTGTTTGAAGGATGAGCGCAGAAAGAGAGGAAAAG
CCATGCAACCAGGTGGTACTCGGCTTTTTTCTATTGCTAATATTGAAGGAACTATTGCTCTGC
GAAGGTACACTTTATCATTGGTAAGCCATTTGAGGTACAATAGGATTAAGAACTCTATTGGG
ATAGGGATTAATCCAGACTCCATGGAGTGGACGACATTGTATTGCGCCTTAATAAAGCAGGG
ACGTTCTAAATTCTTTACTACTGACTTTACAAATTTTGGGGCAGGGCTCAACTATTATGTGGG
TATGAAATTTGCAGATTTGATTTTGGATTTTATGAGCCAATTTGGTATGGAGAGACGGAGTGT
GGACTTGAATGTGGTAGAGGCGTTGATAACGGAATTGATGGGTAGTTATCATGTAGCTGGAG
ACATTGTATATCGGACTAGTAGTGGGAGCCCATCTGGAGCGGCTATAACTGGAGAAATAAAT
AGTTTTGTACATTTGATGTATGTAACTATTAGCTGGCTCATCATAGGCACAGTGGTGCACAGG
TTGAACTTTGATGGAGAGAGTACAGGATACCCAGAGTTGGAGGCATATTTGTGTAAAATTG
GTGTGCCGCAGCTGGAATTTACTCTTGAAGATTTGAGCGCCAACACTACTATGGTGGTGTAT

GGTGACGATGGGATATTTTCTGTGTCAGAYGAATATGCAGAGTATTTCAATTCACAAACCATT
AATCTAGTACTGAAGCAACACGGGATAGGTGTCACGGATGCTTCTAAACAGGAAGAGATTG
TGAAGTATACTTCTCTGGAAGGTGTTCAATTTTTGAAACGAGGATTTAGAGAGGACAGTAAT
TTTCCTAGTTGCATTCGACTCTCTGCAATGGATAAAGAAACTATAGAAGAAGTGCCACGGTG
GATTCATGTAAAACCTTTAACTCCTGAGGCGGCTACCCGGGAAAATTGTGAGGCGGCTTTG
AACTTGATATGGGGGCGAGGAGAGAATGAATATAATGCACTGGCGAACAAGTTGAACCGGT
TTTTGCGGGAGATAGGGTTGCAACCAATTTTGAT

>Aedes_Iflavi-like_virus_2_capsid_MT808060
TTGTCAAAAACCAAGGATGATGTTTAGAAAATGGGTAACAAATAGTGAAAGTGATATGGGA
AAGTTCTTGGGGATGATTAGTGATGTTGTGTACCGCAGGACATACCTTATGTTTGAATTTTTC
CTTTTTTTCCTCTCTTCCACACAAAGTGTAACAATGCAAACATGGTATTGGCCTGCGGTACA
CCAAATCGCTACTCCTCCACTAGTAGTTCGACTACCACACCTACCACACCGTAGCGTCTTAT
TTTCCAAAAACCGAGAAACATTCTTGATTAAATTTCCAATGCCCTGCGTTTTATTTTCTGGTG
CAGATCGTTTCAGTGCCAATATTTTCTCCCGATCGATTTCCAAAAGCAAACGCTGTACTTCC
CATCCCAATAATTGCGTGACGGGTCTTATATACTTCTTGCCTGAAGTACACTCTGTTGAGAAT
ACTCAAATGGGTTCTCCAAACTCAAATCATCTAATGCGCTCCCAGCGTCAAGCACTTTGCAA
GCTACGATATTTGCTTTGTTGGTTGCTCTTTTCCTGTTCATGGTGTTTCTGGTGCAGATCGTT
TCAGTGCCAATGGATTCTACCGATGGATTTTGTGATGAAAACTCTGTACTTCTTCAGCCAAC
AAGTGTATACATACACAGTAGCTTCTTCTGCAGTCACTCTGTTGAGAATATCAAATGGGTTCT
CCAAACTTAAATCATCTAATGCACTCCCACTCAAGCACTTTGTCAAGCTACGATATTTGCTTT
GTTGTAGTGTAGAGGTGAACCAGACTATTGCTACTAGAGCCCCAATTTGGCAAGCAAAAAT
AATAGAAGACATAGCAGCGTTGAAGAATATTAATGTACTTCCAATGCGTGGATTTATGCTTG
GAAAATTTGATGTGGAGTTTAAATTGGTTTTACAAGCCACTCCTTTCCAGGCTTGTAGTCTG
TTGCTATCATGGTGTCCCCATCCATATGGGTTGATGCAGTACAATGCCATGGTTGGAACAGA
GGCATTGGAGGCTACTAAATTGAATCAAAGACTTCCAAAGTATGTACCATTTCGAGATTATTT
AGATGTTACAACTGCCGTACAGCGACCAAACGTTATTTTGGATGTGGCTTCAGGAGGAGAA
GCTGTGTTGCGAGTGGGCCAAAAATTTCACCGTACATTCATTCGCAACTTTGATTTTGACCG
AGTCACTGGAGTGAATATGGGCATTCGAGGGGGATATCAAGGATTGTTGTTGTTACATCAGT
TGACTCCAGTGATGGTTGGTAACGGAACACAATCATCATTTACTGCACGCATTTTCTATCGTT
TTGTAGAAGCAAAATTGACTGCAATGACTCAACCAGTGGAAGCCCGGACTAAAGTGGAATT
ACCATACATGTATGTAATTCCAAAAAATGAAGACGCAAAATTTACCGAGATGAGTTCAGTAG
CTGCCGAAGAGCAAAAGAGAACGATACTACGTACTAAATATCGTACGCAAGGGCCGGCTAT
GAGTGCACTTTCCTTGATAAGGGGAGTAGGAATGACAGCTACAGGATTAATAGCTTCAGTA
GAAGCTGTCTCTATGATACGAGGCACGAGACCACGAAATCGAGATAAACCCAATGATATAGT
AGGACAAGTTGTTGTTTCTCCCCGACCTAGACCAAATTTTACCAATGGGGAAGGAATTGAT
GGAGCTGCTGTTATTGGATTGAGTTTTTCGGAACTGACAAATGTTTTGGAAAGTTTTAGTGA
TGAACCAACATCGTATAAGGATTTCGTTAACAGATGTACTTCCTTAACTTGGGTCATTTTGGG
GAATAGTCGCCCAACCATATCATGTATCTTTGTCAGTTCAAGCGATACATGTAGTGTGTGGCG
ACTATTCCCCAAAATGAACGAAGTTCAGGCAGTATCATCATCATGTCTCTTATACAAATCTTT
AGCGGACGACGACCTACGCGGTGTGAAGATTGGGTGGAG

>Aedes_Iflavi-like_virus_2_rdrp_MT808061
TTTTTTCTATTGCTAATATTGAAGGAACTATTGCTCTGCGAAGGTACACTTTATCATTGGTAA
GCCATTTGAGGTACAATAGGATTAAGAACTCTATTGGGATAGGGATTAATCCAGACTCCATG
GAGTGGACGACGTTGTATTGCGCCTTGATAAAGCAGGGACGTTCTAAATTCTTTACTACTGA
CTTTACAAATTTTGGGGCAGGGCTCAACTATTATGTGGGTATGAAATTTGCAGATTTGATTTT
GGATTTTATGAGCCAATTTGGTATGGAGAGACGGAGTGTGGACTTGAATGTGGTAGAGGCG
TTGATAACGGAATTGATGGGTAGTTATCATGTAGCTGGAGACATTGTATATCGGACTAGTAGT

GGGAGCCCATCTGGAGCGGCTATAACTGGAGAAATAAATAGTTTTGTACATTTGATGTATGT
AACTATTAGCTGGCTCATCATAGGCACAGTGGTGCACAGGTTGAACTTTGATGGAGAGAGT
ACAGGATACCCAGAGTTGGAGGCATATTTGTGTAAAATTGGTGTGCCGCAGCTGGAAACGA
ATATGCAGAGTTGGAGGCATATTTGTGTAAAATTGGTGTGCCACAGCTGGAATTTACTCTTG
AAGATTTGGGCGCCAACACTACTATGGTGGTGTATGGTGACGATGGGATATTTTCAGTGTCA
GACGAATATGCAGAGTATTTCAATTCACAAACCATTAATCTGTACTGAAGCAACATGGGATA
GGTGGCACGGATGCTTCTTAACAGGAACACTATTAATGGTTTGTGAATTGAAATACTCTGCA
TATTCGTCTGACACTGAAAATATCCCATCGTCACCATACACCACCATAGTAGTGTTGGCGCTC
AAATCTTCAAGAGTAAATTCCAGCTGCGGCACACCAATTTTACACAAATATGCCTCCAACTC
TGGGTATCCTGTACTCTCTCCATCAAAGTTCAACCTGTGCACCACTGTGCCTATGATGGAGC
CAGCTAATAGGTACATACACCAAATGTACAAAACTATTAATTTCCTCATCATAGGCACAGTGG
TGCACAGGTTGAACTTTGATGGAGAGAGTACAGGATACCCAGAGTTGGAGGCATATTTGTG
TAAAATTGGTGTGCCGCAGCTGGAATTTACTCTTGAAGATTTGAGCGCCAACACTACTATGG
TGGTGTATGGTGACGATGGGATATTTTCAGTGTCAGACGAACAGGCAGAGTATTTCAATTCA
CAAACCATTAATCTAGTACTGAAGCAACAAGGGAGAGGTGTCAC

>Aedes_permutotetra-like_virus_1_rdrp_MT808062
CCTTCACCTCATCCTCAGGGAGGATGGGGCCGACGGGAAACATCGTCCAGGGGCTGTGCG
GCCAACCCCCCCAAACACTTTTCCTTAAACTCTCTCTCAACATACGAATACAACATGGACGC
ATCAAACCCAGTAATCGGAGAACGCACCACAGTTGCAGACGTGCTTGAAAAAGCTTCAGG
CCTGAAGAGACAGAATTTGGAGTATTTGCGGGAGGTCGCCCGCACAGCCCCTATAGTTTCG
GAGTTCAAGGCCGCCATCCCCAAGGTGTCCCCCGTTAAGGAGGGATCCCTGGACCCGGAG
GTCTTTGAAGTGGTCATGGACGCGATGCCTGTCTGCGGTCTACTGGATCAAGAGGGAGTCC
CTCTCCATCCACCCGGAATCAACATGCTCTCATCTCACGTCAACGTCAACAGACAATTTGGC
TTAACAGCCCCGGCTCCCCTGGCTAGGTCCGCGGCCTATAATGTAATGGGACGACAGATACT
AGGCCCAAGGGAGCCGGTGGACTGTTTAGCCAAATTGTCTGTTGACGTTGAAGTGATGGGA
CGACAGATACTAGAGCGGAAACATGTGTACACCTCAGGCACCTGGGAAGGTTTTACCAACC
GTCTCAGGGAACAAATGTCCCGGAAGACGGTGCCCATAAGAACCGCCATGGCGCGGATCTC
AGGAGCTAAGAGTGTGTCCATGAAATACGCCCTAGACCGCCTAGACAAGCTGATGCCGCGA
TCCCCCAATAAGGAATGGCCCGGAGTGCATACTCCGCTATCCGACGCCATCCTTAACGGAAT
AGAAATCACCGCAAACTCCTCCGGTCTTTTCACAGCTCCGTGCCCCCAAACAAATGGACGC
ATCAAACCCAGTAATCGGAGAACGCACCACAGTTGCAGACGTGCTTGGAAAGCTTCAGGC
CTGAAGAGACAGAATTTGGAGTATTTGCGGGAGGTCGCCCGCACAGCCCCTAGGACGATGT
TTCCCGTCGGCCCCATCCTCCCTGAGGATGAGGTGAAGGCTATAGTTTCGGAGTTCAAGGC
CGCCATCCCCAAGGTGTCCCCCGTTAAGGAGGGATCCCTGGACCCGGAGGTCTTTGAAGTG
GTCATGGACGCGATGCCTGTCTGCGGTCTACTGGATCAAGAGGGAGTCCCTCTCCATCCAC
CCGGAATCAACATGCTCTCATCTCACGTCAACGTCAACAGACAATTTGGCTTAACAGCCCC
GGCTCCCCTGGGCCTGGTGTCTGCTGCTGAAGTGATGGGACGACAGATACTAGAGCGGAA
ACATGTGTACACCTCAGGCACCTGGGAAGGTTTTACCAACCGTCTCAGGGAACAAATGTCC
CGGAAGACGGTGCCCATAAGAACCGCCATGGCGCGGATCTCAGGAGCTAAGAGTGTGTCC
ATGAAATACGCCCTAGACCGCCTAGACAAGCTGATGCCGCGATCCCCCAATAAGGAATGGC
CCGGAGTGCATACTCCGCTATCCGACGCCATCCTTAACGGAATAGAAATCACCGCAAACTCC
TCAGCAGGAGCCCCATACTGGAGGAACAAAGGAGAATGCATGTCCCAGATCGTCGATGTGG
GTCTGCCTATCCTAATAAAAGCCCTCAAAGAGAACAGCATAGCAGACCTCTGGAGAACCAA
CCCAGAGATGCTGCTGTGTGAAGTGAAGAACAAACTGGACAGATACCTGGAAACTGAGCT
GAGCACCAAGACCCGCCCCTATGTGTGCGTACCTGCACACATAGCGTTTCTGTGCTCGGTTC
TTTCCCAGGGCTTCCAAGCAACTCTCCACACCTTTGACAGGGATCCAAACTCCGCCAATGC
ATATGGGTTCTCCTCCACTGACGGAGGACTTCAAAGGATGTACACCTGGATGACGCACAAG
CCTACAAGCAGAGGTAAATTCTGCAGCTACGGAGATGACACCCGCCTAGTGGTCCTGCACG

AAGGTCGGGTCTGGATTGTAGATCCTGACGCTAAGCAAATGGACGGATCAATAGATGAGGA
TGATGTGAAACTAACCATTGAGTGGATCCTGAGGCACCTTTGCCAGGACTCAGGAGACCCT
CTGTCGCACTTCTGGAGAGGAGTGGCCAAACTCTGGCACCACACGGCAACAAACCCCCAC
TTTCTAGTAGACGGAAAGAAGGTCTATCGGAAGAGGTCCAAAAACGGTCTCATGACTGGG
ACACCAGGGACAACCCTCTTTGACACGGTCAAATCTGTTCTCCTGTGGGACTCCTATTTGG
AGACCTGCCAGCTCCAAAACTGGAACCCCCTAGATGGAAACAGGGCCAAGAGCTACATGG
CCGAGAACGGATTGGTCATCAAGGAGGGAACATGGAACCCTGTACAACTACCAGAAATGA
GGCATGGACAGCTCATGACAGACCACAAGTTTCTAGGGGTCCAAATGCTGTGCCTGGAGTG
GCAGCACCAATACATATTTGTGCCCACCCTCCCGGAAGACGAAGCTCTTGAGATGATACTGG
TTCAGAAGGACAACCCCTTTACCAGCAAGAAGGAGTCGCAAACCATAAAAATGAGGCGGC
TCTTTGACAGGATGAGGGGTCTCTTCCTAACCTTTGGATTCTCAATTCCGAGCATCAGAAGG
GTCATATACAATATAGTAAACAAAATCCCCGCTGAGTACGTCCTGATGCAAACTCAGGTAGA
AGGAGGAGAAAAGCCCGAGCACATAACCCTACAGGAAGTAACCTACCCTGACTCTACAGG
TTTCCCATCGATAGAGTTCTGTTACAACCTGTACTCGGGGCTCTTTGAAGCCGACATTGGGT
GGGATCAGATATATCCAACCATGCTACCTACACTGAGGAACTTAAGGACAGAGAGACGCCA
ATTAGGAAAGGAGCTGCGACTAGCGACCACCGAGCTGGATGACAAAACCAGGCAGGTGGT
CGCTGAGGAGGCCCCAGACCCCCCCCCTCTGGACCCCATTATGGAGGTGGCCGAGGCCATG
AACCCGATCAACTTCCCACCCCTCGGAGAGGTGAATAAGAGAAGCTTTATTGGTAAGGTCA
CGGACTCATCCCACGTGGAACCCAGGAAGAGAATACCCACCCGAGCCGAGGTGGTTTGGA
AGGCTGTCAATGAGAGGACCGTCACCCAGGTAGGAACCCTGCTTGAGGAGTTCTCTCTTAA
CCCGACCAGCCTCTACACCATAGCCCGGGAACACGGTCTCTACCTAACCGGTCTAGAACCA
GGGGACCTCTGCTCGGTCTACCCCATTGCAACACCCTTCCCCACCATCCAGGAGGGTCTCA
CCCAGGAGTGGAAACAGAACCAGAACAAGGTGGATGCCGGAACAACCAAGAGAACCCAG
GCTCTGCAACGCCAGGCCCAGGAACAGGTTGTTAGAACGGCTCCGACCCTTGTCCGGATG
GACCACGATGTCCTGTCCGGCCTGGAGTTCGACACTCCCAGGGATGAAGCTCATGCCGTGG
AACTCCTGAACCCTGCCCTGAACAGAGTTTTTGGAGGAATGCGCTGGTCCACCAAGGAGG
TCCAGCCCCATGAGCCAAACCCAGTCACAGCCGTGCTCAGCGTCTGGACTGGTGACGGGT
GGGAACCCGTCGCGGAGGCCAGGAGTGCCAACAAGAGGCTTGCTCAGCAGTACATAGCCC
GGCAAATCCTCTTGCAGAACGGAGTGGAACCCCCCACTTCTGTGTTCACCCCCGCCATACC
GCCCCCCCCTGGAGACAAACTTGACTGGGCGACGGACTACGAAACCACGATGGGTCCCTAT
CGCTCCCCCATAGTCGTCGAGTTTGATAACGGCGAGACTCGGGAGGTACACCCCGAGTCTC
GCCGGTATCAAACTCGACGCCTATGGAGGAGCGATAGGGAGCCAGCATGGTTGGGTTTGAT
AACGTGGAGAAGCGGAGTGTATCTCCCGAGTATCCCCGTTCTCCAACTCGAGACCGAACGA
TACCTCGTGCGCCCCCATCGTAGTCACGTTTATAAAAGCGAGAAGCGGGAGGGACCCTTGT

>Aedes_permutotetra-like_virus_2_strainAP60_capsid_MT808063
GGGGATTCAGGTGGGGGTCTGGAGCTGGTTGTGGTGGGAAAAATCTTTGCCTCCTCATGTG
CCCTCCCCTCCCACTGCGGAGCGATCGTAGCCACCGTGGTGGAGGCGGGATGGTTCTCTGA
CTCAATCCTATGCGTAGGAGTGGGGCCGGAGAGACCCCCCACATTGGGGGGACAACCCGT
GCCAACCAACTACCTCCCCTACTACTCTGTCGAGTTTTCGGCAGGCCCTGTGGGTTTTCATA
ACCACTTTCTTGTGGATAGGGACCCTCAGGGTTGGGAGCTAGTTCCTCGCTACACAGTTGT
GGGGACCGGAGTTCTTCTCGATCTAGAGGGGAAACACAGTGTCCCCAAGGTATACGAAAC
GTCCGACGGACGCTTCTGCGTAAAGGGCTTGGTTGACGGGGAGGTCCTGTCAAGGGGATG
CCGAGCGTCCTCTTTGACTGACCTCCCCTGTGCTTGCAGACTTCCGGTCCTTCAAGGCCCG
GCTGTCGACCCTGAGGAGGCCCACAGATCTGCCGAGATAGGAAGCATCTGGACCTACAGG
AAGACACCTCAGGGTGTCCGAGCCTTGTTCGGAGATGTCGACTCTTCCCTTCAGGAGGAAT
GCCAGGCTTACCTCCAGACTGCCACCTATGTCTCACAGTACCACAAGTCCGCCGTGGTGCA
CCTCCTACAGCTGGTGTGGACCCTGGTCCTGAAACTCCTCTCCCTTCTATGGAGCTGGATTG
ACAATGGACTAGTGCTCCCCACCACCCCCGGGGTGTCATCCGCCACGGTGTGGACGGCTGT

GCTGAGTGGCGTGGCCCTGTCGAGAGGGCTCTGGCCCAGTAATTTGTATGTGCAGTTGGCT
GTCCTTACTTCTGGCTTTCTGTTTCTTCTT

>Aedes_permutotetra-like_virus_2_strainAP59_capsid_MT808064
CTAGTCTACGATGCCACCCAGGAGTACTGCGAGGGGACGACCTGCATGCGGTACTATCGCA
GCAGGCTCCTCCCCAACGACCCTGTGAGAGTCGAGGACACTTGGAAGCTGTTCCACCACC
ACCCACACGTCAGCCCCCACCTAATCCCCTGGGGGGCGTCCGATGCTTGCAAAGTCAATGT
GTCGGTCGCCCTAGGACCCCAATTGGGGTTCGTCTACAGGGGATTCAGAGTGGGGTGTCTG
GAGCTGGTTGTGGTGGGAAAAATCTTTGCCTCCTCATGTGCCCTCCCCTCCCACTGCGGAG
CGATCGTAGCCACCGTGGTGGAGGCGGGATGGTTCTCTGACTCAATCCTATGCGTAGGAGT
GGGGCCGGAGAGACCCCCCACATTGGGGGGACAACCCGTGCCAACCAACTACCTCCCCTA
CTACTCTGTCGAGTTTTCGGCAGGCCCTGTGGGTTTTCATAACCACTTTCTTGTGGTTAGGG
ACCCTCAGGGTTGGGAGCTAGTTCCTCGCTTCACAGTTGTGGGGACCGGAGTTCTTCTCGA
TCTAGAGGGGAAACTCAGTGTCCCCAAGGTATACGAAACGTCCGACGGACGCTTCTGCGTA
AAGGGCCTGGTTGACGGGGAGGTCCTGTCAAGGGGATGCCGAGCGTCCTCTTTGACTGAC
CTCCCCTGTGCTTGCAGACTTCCGGTCCTTCAAGGCCCGGCTGTCGACCCTGAGGAGGCCC
TCAGATCTGCCGAGATAGGAAGCATCTGGACCTACAGGAAGACACCTCAGGGTGTCCGAG
CCTTGTTCGGAGATGTCGACTCTTCCCTTCAGGAGGAATGCCAGGCTTACCTCCAGACCGC
CACCTATGTCTCACAGTACCTCAAGTCCGCCGTGGTGCACCTCCTACAGCTGGTGTGGACC
CTGGTCCTGAAACTCCTCTCCCTTCTATGGAGCTGGATTGACAATGGACTAGTGCTCCTCAC
CACCCCTGGGGTGTCATCAGCCACGGTATGGACGGCTGTGCTGAGTGGCGTGGCCCTGTCG
AGAGGGCTCTGGCCCAGCAATCTGTATGTGCAGCTGGCTGTCCTTACCTCCGGCTTTCTGTT
TCTTCTTTCGGCCCTTCACAGGGCTCCCACTCTTTCGAAGTGATCAGAGGGGATACCTCCCC
TCTCACCCCTTCGAAAGAGTGGGAGCCCTGTCGAGAGGGCTCTGGCCCAGCCATCTGTAT

>Guadeloupe_mosquito_viruses_strain_AP03_segment1_MT808014
CUUUCUCACUGACAUAAGUGCCAGUGAGAAAGCUAUGCCGGGAAGUUCUUUUGAGGUA
GCUAAAGUCCUGCCUACUUUCCAGGCUAUGGUAAUGGCAUCGUUAGAUGGGAUUACCA
UAGCCUGUAAAACGAGAUGGAAACUGUGAAUGUUAUUUACACCACUGUGAGUGACGCG
GUAGGAUAUAUUUCCAUACCACUGCAGGUAGUCGCCUUAAUGAUGUCAGCUAUAAUGA
UGUCAUUUGUGGUGAACCCGUUGAAUAAAGCCAUGUUGGCGGUAUCCUUCUACGUGCA
AACCGGAUUUCUUUUGAAAUACAUAUGGAGCCUUGCGCCAUCGAUUGAAGGGAUAAUC
CACGAGCGAUUGUCAGAGACGUAUUCUAAAGUGCAUAUGGAAACUUUGGAAACGUUUU
UAUUAGACUAUAAGGUCUAUUUAAUGGCGAUAGCAUGUGGUUUUGUUAUCCUUCUUUC
GAGCUACAUAAGUGCCAGCUCCAGCUUGGCCAUUAGGCACCUCAAAAGACCACCGUGC
GUUUGUGGUGCCUAAUGGCCAAGCUGGAGCUGGCACUUAUGUCAGUGAGAAAGCUAUG
CCGGGAAGUUCUUUUGAGGUAGCUAAAGUCCUGCCUACUUUCCAGGCUAUGGUAAUGG
CAUCGUUAGAUGGGAUUACUUAUCACGUUAUGGGCCAGUGUUUCUGGGUCGAUGAAGG
CUUAGUGACAGCAGCCCACGUGAUUGAGGGUUUUGAAUAUUUGUGUAUCUACUUCGAU
GCGUUUCUCUUCGUCCUUGUAGAACCAAAAACAUUCGAGAUCGGCAGAGUGUUGGUUU
GCGUCAUACUUCCUAAUCCCCUUUGGAUAACGCAAAAAUUGGGUUUGAGCAAAGCAAA
GUUUGCGAGGCUGGCGGUCGAAAAAGAUGCCGGCUUGAGUGUUAACAUCACAGCCAUG
GGAAAGAGAACGAUAGGAUUCCUAGAUAGACAUCCACAAUUUGGGUAUGUUCGAUAUA
GUGGAAGCACCAUUAAAGGAUUUAGUGGAGCUCCUUACUAUUUGGCAGGACCAUAUU
UGGAAUGCAUCUGGGGUCGGACUCUAAGAACCUUGGUUAUGAUGGAGCAUUCUUGAAA
ACUGAACUCAAGCCCAGUCGGGUUAUAAGAAUUCCGUUAUAUUUAAGAACUGAAGAUA
GUGCAAGUUUUCUAGAGCAGCAGUUAGAGCGUUAUGAAGAAGUAGAGUAUUCCAGAUC
CCCGUAUAAUCCCGAUGAAUAUAAGGUUAGGAUUGGAGGGAUGUAUCAUAUUGUUGAU

GAGGAAGUGUUGAGUACCGUCUUAAAGAGAAGAUCCGCUAAGCGAGCACAGGGGGAUU
UACAAUAUUUGGCUGAACAAGCUGAAGUUGGGCCCCUCAUUGAUCUCGAAGCUGUAAC
CACUAAGGGAGACCUUACUAGGUCUCAACAGAGUGGACAAAAACAGCACCAGGAGCUU
UCUACUGAGACGGAUCCGAUAAUAGCCAUGACUAAAGGGCUAUUGUCAGAAUUUGGCA
AAACAUCAGUGUACGUUCCUCCAGAACCUAAGAGUAAAGACCAAGGGACAUGCACUAG
UUGUGAGUCUUGUGGCGCCUAUAUAUAUUGGGGGGAGUCUAUGCCACGGGCCCCAACG
CCUACUGCGGAUGCCAAGAAAGCGGAAGAUUUACCUUUAGCGCCUAGGAAUGCCAUGA
CGUUUAAUGAUUCGGAAAACCUGAUCAGGGCUCCAGCUGUAGUUGUUGGGGCCCGUGG
CAUGGAAUAUCAGAGGGAAAGUGUACAGAGCCACGACUUGCCAAGAUGCAAGAAAAUG
GAUUGCAUGUGCCAGAAACCUUUGGAGACCUGCCUUAUGGAAUCCCGUACAUUGACGC
GUGCUCCGCUAAAAGAAGCUUCCGCACGCACUGUCAGAAACAAAAACAGACGGAUGCG
CCGCCAACAGCAGAAGAAAGAACUAGAGCAGUUCAAGCAGCUCUACGGACCUAUUCUG
CAUGGUGGUGUGACCUCGCAUCCGCAGCAGAUCCCCACAAAUGGUUUGAUAGGGAAUU
CAAUAAAGCACUAGAGGAGAUAGAUAUGGAUAGCACUCCUGGCUACUGUAUUUUGACU
CUUUUAGGCUCUACGAAUGCGCAAAUCUUCGGUUGGAAUGGUAUAUGCUGUGAUCCGG
AUAGAGUUAGUAUGGUACGAGAGCAUGUCAAAUUAAGGUUCAAUGAGUUGUUGGCAG
GUAAGGCUGUGAGCGAUUGUAUUAAGGUUUUUGUGAAGCAAGAGCCGCAUAAAAUGA
AGAAAAUUCAGGAAGGUACUUAUCGCUUGAUAAGCGCUAUAACCUUAAUUGAUACCUU
UAUUGACAGAAUUUUGCUUACUUUCAUAGCCAGCCUCCAACUGGACUCCACCGGUGAG
ACGCCAUGUCUCCUCGGGUGGAGUCCAGUUAGAGGCGGAUGGCGUAAGAUAGUGAACA
GGUUUGGCAACCAAGCAGUAAAUUGUUUAGAUAGAUCGGCUUGGGAUUGGACUGUACA
AGGCUAUUUAGUUGAUCUUUGGAUAUUGUUUUUGGAAAAUUUACCAGUGAAUCCUCCA
GACUGGUGGCUUAAGAUGAUGAAACUACGUUUUAAGCUACUGUUUGAGGAGGUUUGG
UUUCGGUUCGAAGACGGAACAAGAGUAAAGCAGUCUACUGACUACGUAAUGAAGAGUG
GCUGCGACUUGACUAUUUUACAAAAUAGUCUUAGCCAAAGUCUCUUACAUUACGUAGU
CAACAGUAGAUGUGGGAAGCCCAUGACCAUGAAACAACCAUACUCGAUAGGUGAUGAC
ACGGUACAGGAAGCUAUGGAUUGGCUUCUAGACUAUGUUAGACAACUCGAGAUAUUGG
GAAUCACGGUCAAAGGAGCUAAAGUCCAGCAUUGGGUUGAAUUUGCGGGGAUCUAUUC
GUACAGGAAAACUUGUUAUCCGGCAUACUGGCAAAAACAUCUUUUUAACCUUAAGUAU
ACGCAAAAUCUUGGGGAGACUUUGCGUAGUUAUCAGUAUUUGUACGUCAAUGAGCCUG
UGAUGUACGAAUUUGUGUGUAGAGUGGCUCGUGAGUUAGGUCCUCGUUUCGUCUUGCC
UAAGAUAGAAGCACUAGACAUAAUGAAUCAACCAAAUUGAUCCUAUGUCUAGUGCUUC
UAUCUUAGGCAAGACGAAACGAGGACCUAACUCACGAGCCACUCUACACACAAAUUCG
UACGUCAAUGAGCCUGUGAUGUACGAAUUUGUGUGUAGAGUGGCUCGUGAGUUAGGUC

>Guadeloupe_mosquito_viruses_strain_AP03_segment2_MT808015
UUGGUGCUUCCUACUUUCGUAUGUCUUCUUCGUCACUUUAAUUUUUAUAAUUGUAACA
CAAUGAGUAAAAAGUUUAAGAACCAUCUUCGCAUCUUUAACUGCUCAAUUAGCGUCGA
UGAGACGCUUGCCAAACCGCAACCUCACGCUUCUGCUAAGAAACGGCGCAAUAGUCGC
AAUAAGAAACGUGGUGGAGCCGACAGGUCCGAGCGGAAUUUCUACUUAUGGACGUAUU
ACCGUUCAGAGAGCCGAGCUCCUGUUCGAGAUAAAGGUGGCCAAAGGUGGCUCUUCCA
CUGGUCAUUAUUACGAAGCCUUGCAGCCGACCUCGACCCGAAUGACGUGGCUAAAGAC
UCUGAGAGAAACGUUUCUCUCAGAUCAUUUGGCAUUCCGCGAGACUUGAGUAUCGCCC
UGCUGUGGGGGCGAUCACAGAUGGCUCUCUGGUCGUCGGAGUCGACUGGGACCCUACG
ACGACCGUGCCUAGUAAAAGCAAGGUCCAAAGUCUGUACUCCUAACUUUCAGGUCCCG
GUAUGGCAGAAGAAGGAGUUGACUCUCCCAGCGUCUCGGUUGCAGAGUCGUAAGUAUU
AUGCUCUCUCAGAUUCAGGGUAUAUACCCCUGAUAGGCGAUACCUUUUGUGCUCCAGA
UGUCUCCAAAGUGACUUGUUCCUCGAAUCCUUAUAGGGAACAGUACUUUGGAGACAUC
UGGAUCCACUAUAAGGUAACCCUGCAGGGGCCUAAAUAGGGAGACGAGACGCCCCCC

CAUUACCUCCAGGACCUAGUGUGUUUGAUUACCACAUGGUUCGGGAGGUGACCCUAUA
UGAAGUGCGGGCCAUUGUAAGACCUAAAUAUGAGCCCCAACAUAAACAUACUUCUGGA
UCUCAUAAAGGUUUAUGGUACCCAGAAGUGCUGUGGUCGCUAUGGGGGCUCAUAAUUU
AUUCUAGCGGCGGCUGUGGUUUUAGUCAAUGGAACCGAUUUAACUAUCGAGGAUCAUU
AUGGCGUUUCUCACGACGAUUCUACGGUUCAUUUCGUGCUUCAAAUUCUAGGAACAUC
UUUGUUCCUGACAUGGAAUGUCAGACCUGUAGGAUUGGUGCACAUUGGUCUAUCUUUG
AAAAGUCCGUAUUUUCCGAAGAUAGCUAUGUUUGCUUUUACGGUUCAUUUCGUGUCAA
AUACGAACAUCUUUGUUCCUCAGGCUCUUCAAAGAAGUGCACGUUGCUAUUUAUGAUA
CCGUCUGUUGGAUCAAGAUUUCUUAUCAACUAUUAUAACUUUAUCAUGAACCAAUGCU
CCUACGAGCGAUACUUGGUUCAUGAUAAAGUUUUGUCAGUUGAUAGAAAUCUUAGAUC
CAACAGCGGUAAUAAAUGUUACCAUCGUCAUCAACCAAACCUAUGUGGAUUGGUUGAU
GACGAUGUUAACAUGUUAUUACCGCUGUUAGGAUCAUUAAUGACUUUCUAU

>Guadeloupe_mosquito_viruses_strain_AP08_segment1_MT808016
CACGUGAUUGAGGGUUUUGAAUAUUUGUGUAUCUACAAGGACGAAGAGAAACGCAUCG
AAGUAGGACCAAAAACAUUCAAAACCCUCAAUGUGGGCUGCGCACUAAGCCGGCAUUC
GACCGCCACCUCGCAAAAAUUGGGUUUGAGCAAAGCAAAGUUUGCGAGGCUGGCGGUC
GAAAAAGAUCCCGGCUUGAGUGUUAACAUCACAGCCAUGGGAAAGAGAACGAUAGGAU
UCCUAGAUAGACAUCCACAAUUUGGGUAUGUUCGUUAUAGUGGAAGCACCAUUAAAGG
CUUUAGUGGUGCUUCUCACUAUAACGGCAGGACCAUAUUUGAAAUGCAUCAGGGGUCU
GAAUCUCUACUUCUUCAUAACGUCUGGCUGCUCUUGAAAACUGAACUCAAGCCCAGUC
GGGUUAUACGAAACCAGUUAAAUUUAAGAACUGAAGAUAGUGCAAGUUUUCUAGAGCC
GCAGUUAGAGCGUGAUGAAGAAGUACAUCCCUCCAGAUCCCCGUAUAAUCCCGAUGAA
UAUAAGGUUAGGAUUGGAGGGAUGUAUCAUAUUGUUGAUGAGGAAGUGUUGAGUACC
GUCUUAAAGAGAAGAUCCGCUAAGCGAGCACAGGGGGAUUUACAAUAUUCGGCUGAAC
AAGCUGAAGUUGGGCCCCUCAUUGAUCUCGAAGCUGUAACCACUCUGGGAGACCUUAG
UAGGUCUCACCUUAGUGGACAAAAACAGCACCAGGAGCUUUCUACUGAGACGGAUCCG
AUAAUAGCUAUGACUAAAGGGCUAUUGUCAGAAUUUGGCAAAACCUCAGUGUACGUUC
CUCCAGAACCUAAGAGUAAAGACCAAGGGACAUGCACUAGUUGUGAGUCUUGUGGCGC
CUAUAUAUAUUGCGGGGAGUCCAGGGCUAUUCCUACCACGGGCCCCAACGCCUACUGC
GGAUGCCAAGAAAGCGGCAGAUUUAUCUUUAGCGACUGGCAUGCCAUGACGUUUAAUG
AUUCGGAAUAUCUCAUCACGGCUCCAGCUGUAGUUGUUGGGUCCCGCGGCAUCGAAUA
CCAGAAGGAAAGUGAUCAGAACCAAAGAAUGCCAAUAUUCAAGAACAUGGAUUUCAUU
UGCCAGGAACCAUGGGAGACCUGCCAUUAGGAAACCCGUACAUUGACGCGUGCUCCGC
CAAAAGAAGCUUCCGCACGCACUGUCAGAAACAAAAACAGACGGAUGCGCCGCCAACA
GCAGAAGAAAGAACUAGAGCAGUUCAAGCAGCUCUACGGACCUAUUCCGCAUGGAGGU
GUGACCUUACAUCCGCAGCAGAUCCCCACAAAUGGUUUGACAGGGAGUUCAAUAAAGC
ACUAGAGGUCACAGCUACCGCCAACACUCAUUGCAACCUUAAUUUGACUCUUUUAGGC
UCUACGAAUGCGCAAAUCUUUGGUUGGAAUGGUAUAUGCUGUGAUCCGGAUAGAGUUA
GUAUGGUACGAGAGCAUGUCAAAUUAAGGUUCAAUGAGUUGUUGGCAGGUAAGGCUG
UGAGCGAUUGUAUUAAGGUUUUUGUGAAGCAAGAGCCUUCAACAAUGAAGAAAAUUCA
GGACGGUACUUAUCGCUUGAUCAGCGCUGUUAGCUUAAUUGAUACCUUUAUUGACAGA
AUUUUGUUCGCUUGGUUAGCCAGAGCUCAGAUGGAUUCCGUCGGGGAGACGCCAUGUC
UCAUCGGGUGGAGUCCAGUUAGAGGCGGAUGGCGUAAGAUAGUGAACAGGUUUGGCAA
CCAAGCAGUAAAUUGUUUAGAUAGAUCGGCUUGGGAUUGGACUGUACAAGGCUAUUUA
AUUGAUCUUUGGAUAUUGUUUUUGGAAAAUUUACCAGUGAAUCCUCCAGACUGGUGGC
UUAAGAUGAUGAACUUACAUCUUAAGCUACUGUUUGAAGAGGUUUGGUUUCAGUUCGA
AGACGGGACAAGAGUAAAGCAGGCAACGCGAGGAGUAAUGAAGAGUGGUUGCUAUUUA
ACUAUUUUGUUAAAUAGAACCAUCUUCAUUCUCUUACAUUACGUAGUCAACAGUAGAU

GUGGGAAGCCCAUGACUAUGAAACAACCAUACUCGAUAGAUGAUGACACGGUACAGGA
AGCUAUGGAGUGGCUUGUAUACUACGAUGGGCUUCUCCACAUAUUGGGAAUCACGGUC
AAAGGAGCUAAAGUCCAGCAUUGGGUUGAAUUCGCGGGGUUCUGUUUUGACGGGAAAA
CUUGUUAUCCGGCAUACUGGCAAAAACAUCUUUUUAACCUUAAGUAUACGCAAUAUCU
UAAGGUUAACAAAGAGUACUCUACACACUCGUACAUCACAGAGCUCAUUACGUACGAA
UUUGUGUGUAGAGUGGCUCGUGAGUUAGGUCCUCGUUUCGUCUUGCCUAAGAUAGAAG
CACUAGACAUAAGGAAUCAAUUUGAUUGAUUCAUUAUGUCUAGUUCGCUUCUAUCUUA
UGGCAAGACGAAACGAGGACCUAACUCACGAGC

>Guadeloupe_mosquito_viruses_strain_AP08_segment2_MT808017
AUCUUUAACUGCUCAAGUAGCUGCGAUGAAGGUUGCGGUUUGGCAACCUUCAUCGCAG
CUACUUGAGCAGUUAAAGAUGCAAGCUGUUUCUUCAACUGUUUACUCAUUGUGUUAAA
AUAAAGCAAUGAGUAAACAGUUGAAGAAACAGCUUGCAUCUUUAACUGUUUACUCAUU
GUGUUAGAAUAAAGAAAUUAGUAAGUGAUUUUAUGCUUCCUUGGUUAGCCCUCAUAUA
ACUGAGUUUAAUUGCUUUAUUUUAACACAAUGAGUAAACAGUUGAAGAAACAGCUUGC
AUCUUUAACUGCCCAAAUAGCAGCAGACCCCCCCUCCGGCCACCGGCUUCCUCACGCUU
CUGCUGAUAUGCGGCGCUAUUGGCGCAAUAAGAAACGUGGAGGAGCCGCUGGCCCGAG
CGGGGUUUCUGCUGAUGGACGUAUUACCGUUCAGAGAGCUGAGCUCCUGUUCGAGAUU
AAGGUGGCCAAAGGUAGUUCUUCCACGUCUUAUUAUGACCCCCAGCAGGCGAACUCUA
GUCUCACGGAAUGGCCAAAUUAUCUGAGAGAAACUACCACUCAGAUAAUUUGGCAUUC
CGUGAGACUAGAGUAUCGCCCUGCUGUGGGGGCGAUGAAAGAUGGCUCUCUAGUCGUC
GGAGUCGACUGGGACCCUAUGACGACCGUGCCUAGUAAAAGCAAGGUCCAAAGUUGUA
CUCCUAACUUUCAGGUCCCGGUAUGGCAGAAGAAGGAGUUGACUCUCCCAGCGUCUCG
GUUGCAGAGUCGUAAGUAUUAUGCUCUCUCAGAUUCAGGGGAUAUACCCAUGAUAGAC
AAAGCCCCUUGUGAGGUUUUGGCCUAUUUGGCUUGUUCCUCGAAUAAGGAUGAGGAAC
AGUACUUUGGAGACAUCUGGAUCCACUAUAAGGUAACCCUGGAGGGGCCUAAAUAGGG
AGACGAGACGCCCCCCCCAUUACUGUGUGUAGCUAAUUUGUUUGAUUAUCACAUGGUU
CGGGAGGUAACCCUAUAUGAAGUGCGGGCCAUUGUAAGAACUAAAUAUGAGCCCCAAC
AUAAACAGACUUCUGGAUAUUAUAAACCCUUUGAGAUCCAGAUGGCGAACAGGGAUAU
GAUAACGUAUGCAGCGGCGGCUGAGGUUUUAGUCAAUGGAACCGAUUUAACUAUUGAG
GAUAAGUAUAGCGUGUCUCACGAAGGUUCGAAAGGGGACAAAGUGAGUGAAAUUCAAU
GCGAUUCACGCCCCCCGACAUGGAAUCUUGAACCUGUAGGAUUGAUGCACAUUGGUUU
AUCUUUGAAAAGUCCGUAUUUUCCGAAGAUAGCGAUGUUUGAUUUUACGGUUCAUUUC
GUGUCAAAUAGGAACGUUCACUUCUUUAAGUUCCUUAGGAACAAAGAUGUUCCUACUC
AAGAAGUGAACGUUGCUAUUUCUGAGAGCGCGAAGAUCAAGGUUAUUGGUCCUGUAAU
GAAUCUUUUCAAUAGUCAAGCUCCUAGGAGCGAUAAUUGGUUCAUGAUAAAGUUUUGU
CAGUUGAUAGAAAUCUUAGAUCCAACAGCGGUAAUAAAUGUUAACAUCGUCAUCAACC
AAACCUAUGUGGAUUGGAUGAUGACGAUGUUAACAUGUUAUUACCGCUGAUAUGGAUG
CUAAUG

>Guadeloupe_mosquito_viruses_strain_AP09_segment2_MT808018
CUAUCAGAAGCGUGGAGGAGCCGCUGGCCCGAGCGGGGUUUCUGCUGAUGGACGUAUU
ACCGUUCAGAGAGCUGAGCUCCUGUUCGAGAUUAAGGUGGCCAAAGGUAGUUCUUCCA
CGUCUUAUUAUGAAGCCUUGCAGCCGACCUGGACCCGAAUGACGGGGCUAAAGCCUCU
GAGGGGUAGAUUCUCGUCAGAUAAUCCCAGCCGCCUCCGACGACUAGAGAGCCAUCUU
UCAUCGCCCCCACAGCAGGGCGAUACUCUAGUCGUCGGAGUCGACUGGGACCCUACGAC
GACCGUGCCUAGUAAAAGCAAGGUCCAAAGUUGUACUCCUAACUUUCAGGUCCCGGUA
UGGCAGAAGAAGGAGUUGACUCUCCCAGCGUCUCGGUUGCAGAGUCGUAAGUAUUAUG
CUCUCUCAGAUUCAGGGGAUAUACCCAUGAUAGACAAAGCCCCUUGUGAGGUUUUGGC

CUAUUUGGCUUGUUCCUCGAAUAAGGAUGAGGAACAGUACUUUGGAGACAUCUGGAUC
CACUAUAAGGUAACCCUGCAGGGGCCUAAAUAGGGAGACGAGACGCCCCCCCCAUUACC
UCCAGGACCUAGUGUGUUUGAUUAUCACAUGGUUCGGGAGGUAACCCUAUAUGAAGUG
CGGGCCAUUGUAAGAACUAAAUAUGAGCCCCAACAUAAACAGACUUCUGGAUAUUAUA
AACCCUUUGAGAUCCAGAUGGCGAACAGGGAUAUGAUAACGUAUGCAGCGGCGGCUGA
GGUUUUAGUCAAUGGAACCGAUUUAACUAUUGAGGAUAAUUAUAGCGUUUCUCACGAU
GAUUCGAAAGUGGACAACGUGAGCGAAAUUCAAUGCGCUUCGAGACCCCCGACAUGGA
AUGUUGAACCUGUAGGAUUGGUGCACAUUGGUUUAUCUUUGAAAAGUCCGUAUUUUCC
AAUCUUCGCGCUCUCAGAAAUAGCAGUUCACUUCGUGUAUGAUAGGAACAAAAAUGUU
CCUAAGGAUAUAAAAGAAGUGAACGUUGCUAUUUCUGAGAGCGCGAAGACCAAGGUUA
UUGGUCCUGUAAUGAAUCUUUUCAAUAGUCAAGCUCCUAGGAGCGAUAAUUGGUUCGU
GAUAAAGUUUUGUCAGUUGAUAGAAAUCUUAGAUCCACCAGCGGUAAUAAAUGUUAAC
AUCGUCAUCAACCAAACCUAUGUG

>Guadeloupe_mosquito_viruses_strain_AP10_segment2_MT808019
GUUGGUUUUUGUAUUCUUUUAGCACUAUAAUUUAGUAGUUGAAGAAAGAGCAAGCUG
UUUCUUCAACUGUUUACUCAUUGUGUUAAAAUAAAGCAAUUAAACUCAGUUAUUAAAU
CAGUUAUAUGGGCUAACCAGGAAGCAUAAAUCACUUACUAAUUUCUUUAUUUUAACAC
AAUGAGUAAACAGUUGAAGAAACAGCUUGCAUCUUUAACUGCUCAAGUAGCUGCGAUG
AAGGUUGCCAAACCGCAACCUAACGCUUCUGCUAAGAAACGGCGCAAUAGGCGCAAUA
AGAAACGUGGAGGAGCCGCUGGCCCGAGCGGGGUUUCUGCUGAUGGACGUAUUACCGU
UCAGAGAGCUGAGCUCCUGUUCGAGAUUAAGGUGGCCAAAGGUAGUUCUUCCACGUCU
UAUUAUGAAGCCUUGCAGCCGACCUCGACCCGAAUGACGUGGCUAAAGACUCUGAGUG
GUAGUUUCUCUCAGAUAAUUUGGCAUUCCGCGAGACUAGAGUAUCGCCGUGCUGUGGG
GGCGAUGAAAGAUGGCUCUCUAGUCGUCGGAGUCGACUGGGACCCUACGACGGCCGUG
CCUAGUAUAAGUCAGGUCCAAAGUUGUACUCCUAACUUUCAGGUCCCGGUAUGGCAGA
AGAAGGAGUUGACUCUCCCAGCGUCUCGGUUGCAGAGUCGUAAGUAUUAUGCUCUCUC
AGAUUCAGGGGAUAUACCCAUGAUAGACAAAGCCCCUUGUGAGGUUUUGGCCUAUUUG
GCUUGUUCCUCGAAUAAGGAUGAGGAACAGUACUUUGGAGACAUCUGGAUCCACUAUA
AGGUAACCCUGCAGGGGCCUAAAUAGGGAGACGAGACGCCCCCCCCAUUACCUCCAGGA
CCUAGUGUGUGUAAUAAACACAUGGUCCUGGAGGUAAUGCUAUAUGAAGUGCGGGCCA
UUGUAAGAACUAAAUAUGAGCCCCAACAUAAACAGACUUCUGGAUAUUAUAAACCCUU
UGAGAUCCAGAUGGCGAACAGGGAUAUGAUAACGUAUGCAGCGGCGGCUGAGGUUUCA
GUCAAUGGAACCGAUUUAACUAUUGAGGAUAAUUAUAGCGUUUCUCACGAUGAUUCGA
AAGUGGACAACGUGAGUGAAAUUCAAUGCGCUUCAAGACCCCCGACAUGGAAUGUUGA
ACCUGUAGGAUUGGUGCACAUUGGUUUAUCUUUGAAAAGUCCGUAUUUUCCGAAGAUA
GCUAUGUUUGAUUUUACGGUUCAUUUCGUGUCAAAUAGGAACAUCUUUGUUCCUAAGG
CUCUUAAAGAAGUGAACGUUGCUAUUUCUGAGAGCGCGAAGAUCAAGGUUAUUGGUCC
UGUAAUGAAUCUUUUCAAUAGUCAAGCUCCUAGGAGCGAUAAUUGGUUCAUGAUAAAG
UUCUGUCAGUUGAUAGAAAUCUUAGAUCCAGCUUUGCUAAUUCAUGGGGAUAUACCCA
UGAACCAGACCUCCGUAGAUUGGAGAU

>Guadeloupe_mosquito_viruses_strain_AP15_segment1_MT808020
CGACAUAUGGGAUUACUUAUCACGUUAUGGGCCAGUGUUUCUGGGUCGAUGAAGGCUU
AGUGACAGCAGCCCACGUGAUUGAGGGUUUUGAAUAUUUGUGUAUCUACAAGGACGAA
GAGAAACGCAUCGAAGUAGGACCAAAAACAUUCAAAACCCUCAAUGGGGACUGCGCAG
UAUGGGCCAUUGCAACACUGAUAACGCAAAAAUUGGGUUUGAGCAAAGCAAAGUUUGC
GAGGCUGGCACUCGAAAAAGAUGCCGGCUUGAAUGUUAACAUCACAGCCAUGGGAAAG
AGAACGAUAGGAUUCCUAGAUAGACAUCCACAAUUUGGGUAUGUUCGUUAUAGUGGAA

GCACCACUAAAGCCUUUAGUGGUGCUUCUCACUAUAACGACAGGACCAUAUUUGGAAU
GCAUCUGGGGGCGGACUCUAAGAACCUUGGUGUUUCUGAGACAUUAGCGAGAGGUGGG
CGAAGGUGCUUUCGUCUUAAAUUUAACUGGUUUCUUAUAACGACUGGGCUUAGUUCAA
GUUUUCAAGAGCAGCAGUUAGAGCGUUAUGAAGAAGUAGAGUAUUCUAGAUCUCCGUA
UAAUCCCGAUGAAUAUAAGGUUAGGAUUGGAGGGAUGUAUCAUAUUGUUGAUGAGGA
AGUGUUGAGUACCGUCUUAAAGAGAAGAUCCGCUAAGCGAGCACAGGGGGAUUUACAA
UAUUUGGCUGAACAAGCUGAAGUUGGGCCCCUCAUUGAUCUCGAAGCUGUAACCACUA
AGGGAGACCUUACUAGGUCUCACCUUAGUGGUCAAAAACAGCACCAGGAGCUUUCUAC
UGAGACGGAUCCGAUAAUAGCUAUGACUAAAGGGCUAUUGUCAGAAUUUCGUACCGUC
UCAGUGUACGUUCCUCCAGGACGUAAGAGUAAAGACCAAGGGACAUGCACUAGUUGUG
AGUCUUGUGGCGCCUAUAUACAUUGGGGGGAGGCUCGGGAUAUUCCUGCAGCGCCAAC
UGCGGUUGCCAAGAAAUCGGAAGAUCUACUUUUAGCGCCUAGGGAUGCCAUGACGUUU
AAUGAUUCGGAAAACCUGAUCAGGGCUCCAGCUGUAGUUGUUGGGGCCCGUGGCAUGG
AAUAUCAGAGGGAAAGUGUACAGGGCCACGACUUGCCAAGAUGCAAUACCAUGGAUUG
CAUGUGCCAGAAAUCUCUCGAGACCUGCCUUAUGGAAUCCCGUACAUUGACGCGUGCU
CCGCUAAAAGAAGCUUCCGCACGCACUGUCAGAAACAAAAACAGACGGAUGCGCCGCC
AACAGCAGAAGAAAGAACUAGAGCAGUUCAAGCAGCUCUACGUUCUUCUUCUGCAUGU
UGGCGGCGCAUCACGUCUGUUUCAGACCCCCACAAAUGGUUUGAUAGGGAAUUCAAUA
AAGCACUAGAGGAGAUAGAUAUGGAUAGCACUCCUGGCUACUGUAUUUUGACUCUUUU
AGGCUCUACGAAUGCGCAAAUCUUUGGUUGGAAUGGCGUCUCCCGAGAUCCGGAUAGA
GUUGGUAUCCAACGAAAACUUCAUGUCAAAUUAAGGUUCAAUGAGUUGUUGGCAGGUA
AGGCUGUGAGCGAUUGUAUUAAGGUUUUUGUGAAGCAAGAGCCGCAUAAAAUGAAGA
AAAUUCAGGAAGGUACUUAUCGCUUGAUAAGCGCUGUUAGCUUAAUUGAUACCUUUAU
UGACAGAAUUUUGUUCGCUUGGAUAGCCAGAGCUCACAUGGAUUCCGUCGAUGAGACG
UGGCGUCUCCCCGGGUGGAGUCCAGUUAGAGGCGGAUGGCGUAAGAUAGUGAACAGGU
UUGCCAACCAAGCCGUAAAUUCUAAAAAUUUAUCUGCUUGGGAUUGGACUGUACAAGG
CUAUUUAGUUGAUCUUUGGAUAUUGUUUUUGGAAAAUUUACCAGUGAAUCCUCCAGAC
UGGUGGCUUAAGAUGAUGAAACUACGUUUUAAGCUACUGUUUGAAGAGGUUUGGUUU
CAGUUCGAAGACGGGACAAGAGUAAAGCAGGCAACGCGAGGAGUAAUGAAGAGUGGUU
GCUAUUUAACUAUUUUGUUAAAUAGUCUCAGCCAAAGUCUCUUACAUUACGUAGUCAA
CAGUAGAUGUGGGAAGCCCAUGACUAUGAAACAACCAUACUCGAUAGGUGAUGACACG
GUACAGGAAGCUAUGGAUUGGCUUCUAGACUACGUUAGACAACUCGAGAUAUUGGGAA
UCACGGUCAAAGGAGCUAAAGUCCAGCAAUGGGUUGAAAUUGCGGGGCACAAAUUCGA
CAUCAAGGCUCAUUAUACGAAAUACUGGCAAAAACAUCUUUUUAACCUUAAGUAUACG
CAAAAUCUUGACCUAACUACGAGCACUUAYCAAAAUUUGUACGUCAAUGAGCCUGUGA
UGUACGAAUUUGUGUGUAGAGUGGCUCGUGAGUUAGGUCCUCGUUUCGUCUUGCCUAA
GAUAGAAGCACUAGACAUAAUGAAUCAACCAAAUUGAUCCUAUGUCUAGUUCGCUUCU
AUCUUAUGGCAAGCGAAACGGGACUAACUCACGAGCACUGCUACACAAAAUUCGUACA
AUCACAGGCUCAUUGACGUACGAAUUUGUGUGUAGAGUGGCUCGUGAGUUAGGU

>Guadeloupe_mosquito_viruses_strain_AP15_segment2_MT808021
CGCCGGGCCGCGGCUGCCUCACGCUUCUGCUAAGAAACGGCGCAAUAGGCGCAAUAAG
AAACGUGGAGGAGCCGCUGGCCCGAGCGGGGUUUCUGCUGAUGGACGUAUUACCGUUC
AGAGAGCUGAGCUCCUGUUCGAGAUUAAGGUGGCCAAAGGUAGUUCUUCCACGUCUUA
UUAUGAAGCCUUGCAGCCGACCUCGACCCGAAUGACGUGGCUAAGGACUCUGUGUGGU
AGUUUCUCUCAGAUAAUUUGGCAUCCCGCGAUCCGAGACUAUAGGCCAUCUUUCAUCG
CCCCCACAGAGGGCUCUCUAGUCGUCGGAGUCGACUGGGACCCUACUACGACCUUGCCU
AGUACUAGCAAGGUCCAAAGUUGUACUCCUAACUUUCAGGUCCCGGUAUGGCAGAAGA
AGGAGUUGACUCUCCCAGCGUCUCGGUUGCAGAGUCGUAAGUAUUAUGCUCUCUCAGA

UUCAGGGGAUAUACCCAGGAUAGACAAAGCCCCUUGUGAGGUUUUGGCCUAUUUGGCU
UGUUCCUCGAAUAAGGAUGAGGAACAGUACUUUGGAGACAUCUGGAUCCACUAUAAGG
UAACCCUGCAGGGGCCUAAAUAGGGAGACGAGACGCCCCCCCCAUUACCUCCAGGACCU
AGUGUGUUUGAUUACCACAUGGUUCGGGAGGUAACCUUGUAUGAGGGGGGGGCGAUGG
GAGGAACUGGGUAUGAGCCCCAACAUAAACAGACUUCUGGAUAUUAUAAACCCUUUGA
GAUCCAGAUGGCGAACAGGGAUAUGAUAACGUAUGCAGCCGUUGUCCACUUUCGAAUC
AGUGGAAACGCUAUAACUAUCCAUAAUAGUUAUAUCGUUUCUCACGAUGAUUCGAAAG
UGGACAACGUGAGUGAAAUUCAAUGCGCUUCGAGACCCCCGACAUGGAAUGUUGAACC
UGUAGGAUUGGUGCACAUUGGUUUAUCUUUGAAAAGUCCGUAUUUUCCGAAGAUAGCG
AUGUUUGAUUUUACGGUUCAUUUCGUGUCAAAUAGGAACAAUUUUGUUCCUAAGGCUC
UUAAAGAAGUGAACGUUGCUAUUUCUGAGAGCGCGAAGAUCAAGGUUAUUGGUCCUGU
AAUGAAUCUUUUCAAUAGUCAAGCUCCUAGGAGCGAUAAUUGGUUCAUGAUAAAGUUU
UGUCAGUUGAUAGAAAUCUUAGAUCCAACAGCGGUAAUAAAUGUUAACAUCGUCAUCA
ACCAAACCUAUGU

>Guadeloupe_mosquito_viruses_strain_AP17_segment1_MT808022
UUUGUGUAUCUACAAGGACGAAGAGAAACGCAUCGAAGUACGACCAAAAACAUUCGAG
AUCGGCAAUGGGGAUUACGCAGUAUGUCGCAAUCCAAUUACCGAUCUCGCAAAAUUUG
GGUUUGAGCAAAGCAAAGUUUGCGAGGCUGGCGGUCGAAAAAGAUGCCGGCUUGAGUG
UUAACAUCACAGCCAUGGGAAAGAGAACGAUAGGAUUCCUAGAUAGACAUCCACAAUU
UGGGUAUGUUCGUUAUAGUGGAAGCACCAUUAAAGGCUUUAGUGGUGCUCCUUACUAU
AACGACAGGAGCAUAUUUGGAAUGCAUCUGGGGUCGGACUCUAAGAACCUUGGUUAUG
AUGGGGCAUUCUUGAAAACUGAACUCAAGCCCAGUCGGGUUAUAAGAAACCAGUUAAA
UUUAAGAACUGAAGAUAGUGCAAGUUUUCUAGAGCAGCAGUUAGAGCGUUAUGAAGA
AGUAGAGUAUUCUAGAUCUCCGUAUAUUCUCGAGGAUUAUACGGUUAGGAUUGGAGGG
AUGUAUCAUAUUGUUGAUGAGGAAGAGUUGAGUACCGUCUUAAAGAGAAGAUCCGCUA
AGCGAGCACAGGGGGAUUUACAAUAUGAUACAUCCCUCCAAUCUGAAGUUGGGCCCCU
CAUUGAUCUCGAAGCUGUAACCACUAAGGGAGACCUUACUAGGUCUCAACAGAGUGGA
CAAAAACAGCACCAGGAGCUUUCUACUGAGACGGAUCCGAUAAUAGCUAUGACUAAAG
GGCUAUUGUCAGAAUUUGGCAAAACCUCAGUGUACGUUCCUCCAGAACCUAAGAGUAA
AGACCAAGGGACAUGCACUAGUUGUGAGUCUUGUGGCGCCUAUAUAUAUUGGGGGGAG
UCUAGGGAUAUUCCUGAAACGCCUACUGCGGAUGCCAAGAAAGCGGAAGAUUUACCUU
UAGCGCCGAGGGAUGCUAUGACGUUUAAUGAUUCGGAAAACCUGAUCAGGGCUCCAGC
UGUAGUUGUUGGGGCCCGUGGCAUGGAAUAUCAGAGGGAAAGUGUACAGAGCCACGAC
UUGCCAAGAUGCAAGAAAAUGGAUUGCAUGUGCCAGAAACCUUUGGAGACCUGCCUUA
UGGAAUCCCGUACAUUGACGCGUGCUCCGCCAAAAGAAGCUUCCGCACGCACUGUCAG
AAACAAAAACAGACGGAUGCGCCGCCAACAGCAGAAGAAAGAACUAGAGCAGUUCAAG
CAGCUCUACGGACCUAUUCCGCAUGGAGGUGUGACCUUACAUCCGCAGCAGAUCCCCAC
AAAUGGUUUGACAGGGAGUUCAAUAAAACAGUAGAGGAGAUAGAUAUCCAUAUCACUC
CUUGCUACUGUAUUUUGACUCUUUUAGGCUCUACGAAUGCGCAAAUCUUUGGUUGGAA
UGGUAUAUGCUGUGAUCCGGAUAGAGUUAGUAUGGUACGAGAGCAUGUCAAAUUAAG
GUUCAAUGAGUUGUUGGCAGGUAAGGCUGUGAGCGAUUGUAUUAAGGUUUUUGUGAA
GCAAGAGCCGCAUAAAAUGAAGAAAAUUCAGGAAGGUACUUAUCGCUUGAUAAGCGUU
GUAAGCCUAAUCCCAACCUCUAUCUACACAAUUUUACUCGCUUGGAUAGCCAGAGCUC
AGAUGGAUUCCGUCGGGGAGACGCCAUGUCUCAUCGGGUGGAGUCCAGUUAGAGGCGG
AUGGCGUAAGAUAGUGAACAGGUUUGGCAACCAAGCAGUAAAUUGUUUAGAUAGAUCG
GCUUGGGAUUGGACUGUACAAGGCCAUUUAAUGGAUCUUUGGAUAUUGUUUUUGGAA
AAUUUACCUCUGAAUCCGCCAAACUGGUUGCUUAAGAUGAUGAAAUUACGUUUUAAGC
UACUGUUUGAAGAGGUUUGGUUUCAGUUCGAAGACGGGACAAGAGUAAAGCAGGCAAC

GCGAGGAGUAAUGAAGAGUGGUUGCUAUUUAACUAUUUUGUUAAAUAGUCUCAGCCAA
AGUCUCUUACAUUACGUAGUCAACAGUAGAUGUGGGAAGCCCAUGACUAUGAAACAAC
CAUACUCGAUAGGUGAUGACACGGUACAGGAAGCUAUGGAGUGGCUUGUUUCAGAGUA
CGAUGGGCUUCUCCACAUAUUGGGAAUCACGGUCAAAGGGGCUAAGGUCCAGCAUGGG
GUUGAAUUCGCGGGGUUCUGUUUUGACGGAAAACUUGUUAUCCGGCAUACUGGCAAA
AACAUCUUUUUAACCUUAAGUAUACGCAAAAUCUUGGGGACCUAACUACGAGCACUCU
ACACACAAAUUCGUACGUCAAUGAGCCUGUGAUGUACGAAUUUGUGUGUAGAGUGGCU
CGUGAGUUAGGUCCUCGUUUCGUCUUGCCUAAGAUAGAAGCACUAGACAUAAUGAAUC
AACCAAAUUGAUUCACUAUGUCUAGUUCGCUUCUAUCUUAUGGCAAGACGAAACGAGG
ACCUAACUCACGAG

>Guadeloupe_mosquito_viruses_strain_AP17_segment2_MT808023
UCUCUAGCUGCGGAAUCCAAAUUAUCUGAAGACGAGAACUCCACUCAGUCUUUAGCCA
CGUCUUCGGGUGAGAGUCGGCGCAAUAGGCGCAAUAAGAAACGUGGAGGAGCCGCUGG
CCCGAGCGGGGUUUCUGCUGAUGGACGUAUUACCGUUCAGAGAGCUGAGCUCCUGUUC
GAGAUUAAGGUGGCCAAAGGUAGUUCUUCCACGUCUUAUUAUGAAGCCUUGCAGCCGA
CCUCGACCCGAAUGACGUGGCUCUGCUCUCCGACCGGAAAUGCGUCUCAGAGAAACCGC
CGGGCCGCGAGACUAGAGUAUCGCCCUGCUGUGGGGGCGAUGAAGGAUGGCUCUCUAG
UCGUCGGAGUCAACUCCUUCUCUGCCACUACCGGGCCUAGUAAAGUUAGGGUACAACU
UUGGACUCUUGCUUUUCAGGUCACGGUAUGGCAGAAGAAGGAGUUGACUCUCCCAGCG
UCUCGGUUGCAGAGUCGUAAGUAUUAUGCUCUCUCAGAUUCAGGGGAUAUACCCAUGA
UAGACAAAGCCCCUUGUGAGGUUUUGGCCUAUUUGGCUUGUUCCUCGAAUAAGGAUGA
GGAACAGUACUUUGGAGACAUCUGGAUCCACUAUAAGGUAAAGCUGCAGGGGGCUAAA
UAGGGAGACGAGACGCCCCCCCAUUACCUCCAGGACCUAGUGUGUUUGAUUAUCACA
UGGUUCGGGAGGUAACCCUAUAUGAAGUGCGGGCCAUUGUAAGAACUAAAUAUGAGCC
CCAACAUAAACAGACUUCUGGAUAUUAUAAACCCUUUGAGAUCCAGAUGGCGAACAGG
GAUAUGAUAACGUAUGCAGCGGCGGCUGAGGUUUUAGUCAAUGGAACCGAUUUAACUA
UUGAGGAUAAUUAUAGCGUUUCUCACGAUGAUUCGAAAGUGGACAACGUGAGCGAAAU
UCAAUGCGCUUCGAGACCCCUGACAUGCAAGCUUUAUCAUGAACCAAUUAUGCUCCUG
GGUUGCUCUUUGAAAAGUCCGUAUUUUACAAGGACCAAUAACCUUGAUCUUACGGCGC
AUCUCGAGUCAAAUAGCAACGUUCACUUCUUUAAGAGCCUUAGGAACCACGAAAUGAA
CGUUGCUAUUUCUGAGAGCGCGAAGAUCAAGGUUAUUGGUCCUGUAAUGAAUCUUUUC
AAUAGUCAAGCUCCUAGGAGCGAUAAUUGGUUCAUGAUAAAGUUUUGUCAGUUGAUAG
AAAUCUUAGAUCCAACAGCGGUAAUAAAUGUUAACAUCGUCAUCAACCAAACCUAUGU
GGAUUGGUUGAUGACGAUGUUAACAUGUUAUUGGCUGUUAUGGAUUCUAAUGACUUU
CUAUAACUACAAAACUUAUCUGAACCAAUUU

>Guadeloupe_mosquito_viruses_strain_AP21_segment2_MT808024
CUGUGAUUUCCUUGCUCUUCUUUUAUGUUGUCGUCUGCAUCUUCAGAUGUGUUUAAUA
UAUAGUUAUUGCGCCUAUUGCUCCUUUUCGUAGCAGAAUCGUUAGGUUGCGGUUUGUC
AACCUUCAUCGCAGCUACUUGAGCAGUUAAAGAUGCAAGCUGUUUCUUCAACUUUUUA
CUCAUUGUGUUAAAAUAAAGAAAUUAGUAAAUGAUUUUAUGCUUCCUUGGUUAGCCUU
UAUAUAACUUAGUUUAAUUGCUUUAUUUUAACACAAUGAGUAAACAGUUGAAGAAUC
UUUUUGCUGUUCUAGCCGUCUUAGUAGAAGCGUUGAAGGUUGCCAAUCGGCAACCUAA
CGCUUCUGCUAAGAAACGGCGCAAUAGGCGCAAUAAGAAACGUGGAGGAGCCGCUGGC
CCGAGCGGGGUUUCUGCUGAUGGACGUAUUACCGUUCAGAGAGCUGAGCUCCUGUUCG
AGAUUAAGGUGGCCAAAGGUAGUUCUUCCACGUCUUAUUAUGAAGCCUUGCAGCCGAC
CUCGACCCGAAUGACGUGGCUGAAACUUCUGGACAGUAGUUUCGCUCAGAUAAUUUGG
CAUUGCGCGAGACUAGAGUAUCGCCCUGCUGUGGGGGCUAUGAAAGAUGGCUCUCGAG

UCGUCGGAGUCGACUGGGACCCUACGACGACCGUGCCUAGUAAAAGCAAGGUCCAAAG
UUGUACUCCUAACUUUCAGGUCCCGGUAUGGCAGAAGAAGGAGUUGACUCUCCCAGCG
UCUCGGUUGCAGAGUCGUAAGUAUUAUGCUCUCUCAGAUUCAGGGGAUAUACCCAUGA
UAGACAAAGCCCCUUGUGAGGUUUUGGCCUAUUUGGCUUGUUCCUCGAAUAAGGAUGA
GGAACAGUACUUUGGAGACAUCUGGAUCCACUAUAAGGUAACCCUGCAGGGGCCUAAA
UAGGGAGACGAGACGCCCCCCCAUUACCUCCAGGACCUAGUGUGUUUGAUUACCACA
UGGUUCGGGAGGUAACCCUAUAUGAAGUGCGGGCCAUUGUAAGACCUGCAUACGUAUC
AUAUCCCUGUUCGCCAUCUGGAUCUCAAAAGGUUUAUAAUAUCCAGAUGGCGAACAGG
GAUAUGAUAACGUAUGCAGCGGCGGCUGAGGUUUCAGUCAAUGGAACCGAUUUAACUA
UUGAGGAUAAUUAUAGCGUUUCUCACGAUGAUUCGAAAGUGGACAACGUGAGUGAAAU
UCAAUGCGCUUCAAGACCCCCGACAUGGAAUGUUGAACCUGUAGGAUUGGUGCACAUU
GGUUUAUCUUUGAAAAGUCCGUAUUUUCCGAAGAUAGCGAUGUUUGAUUUUACGGUUC
AUUUCGUGUCAAUUAGGAACAUUUUUGUUCCUAAGCCUCUUAAAGAAGUGAACGUUGC
UAUUUCUGAGAGCGCGAAGAUCAAGGUUAUUGGUCCUGUAAUGAAUCUUUAUCAAUAG
UCAAGCUCCUAGGACGCGAUAAUUGGUUCAUGAUAAAGUUUUGUCAGUUGAUAGAAAU
CUUAGAUCCAACAGCGGUAAUAAAUGUUAACAUCGUCAUCAACCAAACCUAUGUGGAU
UGGAGAUGGGAUGGUGACAUGGUGUUGGAUAUUACGCCAAUAAUCACGUUCUAUAACU
CUGUUACGUCUCUGACGACUCCUUACGGUUU

>Guadeloupe_mosquito_viruses_strain_AP22_segment1_MT808025
GUAGAUACACAAAUAUUCAAAACCCUCAAUCACGUGGGCUUAGUGACAGCAGCCCACG
UGAUUGAGGGUUUUGAAUAUUUGUGUAUCUACAAGGACGAAGAGAAACGCAUCGAAG
UAGGACCAAAAACAUUCGAGAUCGGCAAUGGGGAUUGCGACAUACUGCGUAAUCCAAC
ACUGAUAACGCAAAAAUUGGGUUUGAGCAAAGCAAAGUUUGCGAGGCUGGCGGUCGAA
AAAGAUGCCGGCUUGAGUGUUAACAUCACAGCCAUGGGAAAGAGAACGAUAGGAUUCC
UAGAUAGACAUCCACAAUUUGGGUAUGUUCGUUAUAGUGGAUGCACCAUUAAAGGAUU
UAGUGGAGCUCCUUACUAUUUUGGCAGGACCAUAGUUGGAAUGCAUCUGGGGUCGGAC
UCUAAGAACCUUGGUUAUGAUGGGGCAUUCUUGAAAACUGAACUCAAGCCCAGUCGGG
UUAUAAGAAACCAGUUAAAUUUAAGAACUGAAGAUAGUGCAAGUUUUCUAGAGCAGCA
GUUAGAGCGUUAUGAAGAAGUAGAGUAUUCUAGAUCUCCGUAUAAUCCCGAUGAAUAU
AAGGUUAGGAUUGGAGGGAUGUAUCAUAUUGUUGAUGAGGAAGUGUUGAGUACCGUC
UUAAAGAGAAGAUCCGCUAAGCGAGCACAGGGGGAUUUACAAUAUUUGGCUGAACAAG
CUGAAGUUGGGCCCCUCAUUGAUCUCGAAGCUGUAACCACUAAGGGAGACCUUACUAG
GUCUCAACAGAGUGGACAAAAACAGCACCAGGAGCUUUCUACUGAGACGGAUCCGAUA
AUAGCUAUGACUAAAGGGCUAUUGUCAGAAUUUGGCAAAACCUCAGUGUACGUUCCUC
CAGAACCUAAGAGUAAAGACCAAGGGACAUGCACUAGUUGUGAGUCUUGUGGCGCCUA
UAUAUAUUGGGGGGAGUCUAGGGAUAUUCCUGAAACGCCUACUGCGGAUGCCAAGAAA
GCGGAAGAUUUACCUUUAGCGCCGAGGAAUGCUAUGACGUUUAAUGAUUCGGAAAACC
UGAUCAGGGCUCCAGCUGUAGUUGUUGGGCCCGUGGCAUGGAAUAUCAGAGGGAAAG
UGUACAGAGCCACGACUUGCCAAGAUGCAAGAAAAUGGAUUGCAUGUGCCAGAAACCU
UUGGAGACCUGCCUUAUGGAAUCCCGUACAUUGACGCGUGCUCCGCCAAAAGAAGCUU
CCGCACGCACUGUCAGAAACAAAAACAGACGGAUGCGCCGCCAACAGCAGAAGAAAGA
ACUAGAGCAGUUCAAGCAGCUCUACGGACCUAUUCCGCAUGGAGGUGUGACCUCACAU
CCGCAGCAGAUCCCCACAAAUGGUUUGAUAGGGAAUUCAAUAAAGCACUAGAGGAGAU
AGAUAUGUAUAACUCUUCCGGAUACAGCAUUAUGACCAUACUAGACUCUACGAAUGCG
CAAAUCUUUGGUUGGAAUGGUAUAUGCUGUGAUCCGGAUAGAGUUAGUAUGGUACGA
GAGCAUGUCAAAUUAAGGUUCAAUGAGUUGUUGGCAUUGCAAGGCUCUCAUGAAGUUC
UCCAAAGGUUUUUGGGAAGCAUCGGAGCUCUUAUCCAAGCGAACAAAAUUCUGAAAGG
UACUUAUCGCUUGAUAAGCGCUGUUAGCUUAAUUGAUACCUUUAUUGACAGAAUUUUG

UUCGCUUGGAUAGCCAGAGCUCAGAUGGAUUCCGUCGGGGAGACGCCAUGUCUCAUCG
GGUGGAGUCCAGUUAGAGGCGGAUGGCGUAAGAUAGUGAACAGGUUUGGCAACCAAGC
AGUAAAUUGUUUAGAUAGAUCGGCUUGGGUUGCGACUGUAGAAGGCUUCUUAGUCAUC
GAUCUUAAAACGUAUUUUCAUCUUAAGCCACCAGUGAAUCCUCCAGACUGUUGGCUUA
AGAUGAUGAAAUUGCGUUUUAAGCUACUGUUUGAAGAGGUUUGGUUUCAGUUCGAAG
ACGGGACAAGAGUAAAGCAGGCAACGCGAGGAGUAAUGAAGAGUGGUUGCUAUUUAAC
UAUUUUGUUAAAUAGUCUCAGCCAAAGUCUCUUACAUUACGUAGUCAACAGUAGAUGU
GGGAAGCCCAUGACUAUGAAACAACCAUACUCGAUAGGUGAUGACACGGUACAGGAAG
CUAUGGAUUGGCUUCCUGACUACGUUAGACAACUCGAGAUAUUGGGAAUCACGGUCAA
AGGAGCUAAAGUCCAGCAUUGGGUUGAAUUCGCGGGGUUCUGUUUUGACGGGAAAACU
UGUUAUCCGGCAUACUGGCAAAAACAUCUUUUUAACCUUAAGUAUACGCAAAAUACUG
ACAUAACUACGAGCACUGAACAAAAUUUGUACGUCAAUGAGCCUGUGAUGUACGAAUU
UGUGUGUAGAGUGGCUCGUGAGUUAGGUCCUCGUUUCGUCUUGCCUAAGAUAGAAGCA
CUAGACAUAGUGACUCAAUUUGGUUGAUUCAUUAUGUCUAGUGCUUCUAUCUUAGGCU
GAGUGAAACGGGAUGAGUUGGCAAACAAUA

>Guadeloupe_mosquito_viruses_strain_AP22_segment2_MT808026
CGUAAUACGCAUCGCAGAAACCCCGCUGGCCCGAGCGGGGUUUCUGCUGAUGGACGUA
UUACCGUUCAGAGAGCUGAGCUCCUGUUCGAGAUUAAGGUGGCCAAAGGUAGUUCUUC
CACGUCUUAUUAUGAAGCCUUGCAGCCGACCUUAACUCGAAUGAGGUUGCCAAAUUAU
CUGAGUGGUAGUUUCUCUCAGAUAAUUUGGCAUUCCGCGAGACUAGAGUAUCGCCCUG
CUGUGGGGGCGAUGAAAGAUGGCUCUCUAGUCGUCGGAGUCGACUGGGACCCUACGAC
UACCGUGCCUAGUAAAUGCAAGGUCCAAAGUUGUACUCCUAACUUUCAGGUCCCGGUA
UGGCAGAAGAAGGAGUUGACUCUCCCAGCGUCUCGGUUGCAGAGUCGUAAGUAUUAUG
CUCUCUCAGAUUCAGGGGAUAUACCCAUGAUAGACAAAGCCCCUUGUGAGGUUUUGGC
CUAUUUGGCUUGUUCCUCGAAUAAGGAUGAGGAACAGUACUUUGGAGACAUCUGGAUC
CACUAUAAGGUAACCCUGCAGGGGCCUAAAUAGGGAGACGAGACGCCCCCCCAUUACC
UCCAGGACCUAGUGUGUUUGAUUACCACAUGGUUCGGGAGGUGACCCUAUAUGAAGUG
CGGGCCAUUGGAAGAACUAAAUAUGAGCCCCAACAUACCCCGCCAUCUGGAUCUCAUA
AAGGUUUUAAUAUCCAGAUGGCGAACAGGGAUAUGACAACGUAUGCAGCGGCGGCUGA
GGUUUCAGUCAAUGGAACCGAUUUAACUAUUGAGGAUAAUUAUAGCGUUUCUCACGAU
GAUUCGAAAGUGGACAACGUGAGUGAAAUUCAAUGCGCUUCGAGACCCCCGACAUGGA
AUGUUGAACCUGUAGGAUUGGUGCACAUUGGGUUAUCUUUGAAAAGUCCGUAUUUUCC
GAUCUUCGCGCUCUCAGAAUACAACGUUCACUUCGUUUCAAGCCUUAGGAACAUCUUU
GUUCCUAAGGCUCUUAAAGAAGUGAACGUUGCUAUUUCUGAGAGCGCGAAGAUCAAGG
UUAUUGGUCCUGUAAUGAAUCUUUUCAAUAGUCAAGCUCCUAGGAGCGAUAAUUGGUU
CAUGAUAAAGUUUUGUCAGUUGAUAGAAAUCUUAGAUCCAACAGCGGUAAUAAAUGUU
AACAUCGUCAUCAACCAAUCCCAUGAGGUUAAUGGUGAUGCGAUGUUAACAUGUUAUU
A

>Guadeloupe_mosquito_viruses_strain_AP24_segment1_MT808027
CAAAAGACCCCGGCAUGUUUCUACUGCAUAAUGGCCAAGCUGGAGCUGGCACUUAUGU
CAGUGAGAAAGCUAUGCCGGGAAGUUCUUUUGAGGUAGCUAAAGUCCUGCCUACUUUC
CAGGCUAUGGUAAUCGCAUCGUUAGAUGCCAUUACUUAUCACGGUAUGGGCCAGUGUU
ACUGGGUCGAUGAAGGCUUAGUGACAGCAGCCCACGUGAUUGAGGGUUUUGAAUAUUU
GUGUAUCUACAAGGACGAAGAGAAACGCAUCGAAGUAGGACCAAAAACAUUCGAGACC
GGAAAUGUGGAUUGCGCAGUAUGUCGUAAUCCCACACUGAUAACGCAAAAAUUGGGUU
UGAGCAAAGCAAAGUUUGCGAGGCUGGCGGUCGAAAAAGAUGCCGGCUUGAGUGUUAA
CAUCACAGCCAUGGGAAAGAGAACGAUAGGAUUCCUAGAUAGACAUCCACAAUUUGGG

UAUGUUCGUUAUAGUGGAAGCACCAUUAAAGGAUUUAGUGGAGCUCCUUACUAUUUUG
GCAGGACCAUAUUUGGAAUGCAUCUGGGGUCGGACUCUAAGAACCUUUCUUAUAAUGC
UGCAUUCUUGAAAACUGAACUCAAGCCCAGUCGGGUUAUAAGAAACCAGUUAAAUUUA
AGAACUGAAGAUAGUGCAAGUUUUCUAGAACUCCAGUCAGAGCGUCAAGAGUUCUUAG
AGAAGUCACGAUCUCCGUAUAAUCCCGAUGAAUAUAAGGUUAGGAUUGGAGGGAUGUA
UCAUAUUGUUGAUGAGGAAGUGUUGAGUACCGUCUUAAAGAGAAGAUCCGCUAAGCGA
GCACAGGGGGAUCUUCUCUUUAAGACUGAACAAGCUGAAGUUGGGCCCCUCAUUGAUC
UCGAAGCUGUAACCACUAAGGGAGACCUUACUAGGUCUCAACAGAGUGGACAAAAACA
GCACCAGGAGCUCUCUACUGAGACGGAUCCGAUAAUAGCUAUGACUAAAGGGCUAUUG
UCAGAAUUUGGCAAAACCUCAGUGUACGGGAUUCCAUACGGCAGGUCUAAAGUUUCUG
GGACAUGCACUAGUUGUGAGUCUUGUGGCGCCUAUAUAUAUUGGGGGGAGUCUAGGGA
UAUUCCUGAAACGCCUACUGCGGAUGCCAAGAAAGCGGAAGAUUUACCUUUAGCGCCU
AGGAAUGCCAUGACGUUUAAUGAUUCGGAAAACCUGAUCAGGGCUCCAGCUGUAGUUG
UCGGGGCCCGUGGCAUGGAAUAUCAGAGGGAAAGUGUACAGAACCACUACUUGCCAAA
AUGCAAGAAAAUGGCUUUCUUCUGCCAGAAGGCUUUGGCGAUCCGUCUUUUUGGAUCC
CGUACAUUGACGCGUGCUCCGCUAAAAGAAGCUUCCGCACGCACUGUCAGAAACAAAA
ACAGACGGAUGCGCCGCCAACAGCAGAAGAAAGAACUAGAGCAGUUCAAGCAGCUCUU
GGGACCUGCUCCGGAUGGAGGUGUGACCUCACAUCCGCAGCAGAUCCCCACAAAUGGU
UUGAUAGGGAAUUCAAUAAAGCACUAGAGGAGUGCUAUCUUGAUUUGGCUCCUCGCUA
CUGUAUCUUGACUCUUUUAGGCUCUACGAAUGCGCAAAUCUUUGGUUGGAAUGGUAUA
UGCUGUGAUCCGGAUAGAGUUAGUAUGGUACGAGAGCAUGUCAAAUUAAGGUUCAAUG
AGUUGUUGGCAGGUAAGGCUGUGAGCGAUUGUAUUAAGGUUUUUGUGAAGCAAGAGC
CGCAUAAAAUGAAGAAGCCUCAGGAAGGUACUUAUCGCUCGAUAAGCGCUGUUAGCUU
AAUUGAUACCUUUAUUGACAGAAUUUUGUUCGCUUGGAUAGCCAGAGCUCAGAUGGAU
UCCGUCGGGGAGACGCCAUGUCUCAUCGGGUGGAGUCCAGCUAGAGGCGGAUGGCGUA
AGAUAGUAAAUAGCUUUGCCAAACCAAGCAGUAAAUUGUUUAGAUCGAUCGGCUUGGG
AUUGGACUGUACAAGGCUAUUUAAUGGAUCUUUGGAUAUUGUUUUUGGAAAAUUUAC
CAGUGAAUCCUCCAGACUGGUGGCUAAAGAUGAUGAAACUACGUUUCAAGCUACUGUU
UUAAGAGGUUUGGUUUCAGUUCGAAGACGGAACAAGAGUAAAGCAGGCAACGCGAGGA
GUAAUGAAGAGUGGUUGCUAUUUAACUAUUUUGUUAAAUCGUCUCAGCCAAAGUCUCU
UACAUUACGUAGUCAACAGUCGAUGUGGGAAGCCCAUGACUAUGAAACAACCAUACUC
GAUAGGUCAUCACACGGUACAGGAAGCUAUGGAUUGGUUCCUGUACCGUGUCAGUCAC
CUCGAGAUAUUGGGAAUCACGGUCAAAGGAGCCAAAGUCCAGCAUUGGGUUCAAUUCG
CGGGGUUCUGUUUCGACGGGAAAACUUGUUAUCCGACAUACAGGCUCAUUGAUCGUAC
AAAACUGAUAACUACGCAAAGUCUCGACCUAACUACGAGCACUUAACAAAAUUCGUAC
GUCAAUGAGCCUGUGAUGUACGAAUUUGUGUGUAGAGUGGCUCGUGAGUUAGGUCCUC
GUUUCGUCUUGCCUAAGAUAGAAGCACUAGACAUAAUGAAUCAACCAAAUUGAUCCUA
UGUCAAGUUCGCUUCUAUCUUAUGGCAAGCGAAACGGGAUAACUCGGCACUC

>Guadeloupe_mosquito_viruses_strain_AP24_segment2_MT808028
UUCUUACGCCUUUGGCCGUUCUAGCAGAAGCGAUGAAGGUUGCGGUUUGGCAACCUAA
CGCUUCUGCUAAGAAACGGCGCAAUAGGCGCAAUAAGAAACGUGGAGGAGCCGCUGGC
CCGAGCGGGGUUUCUGCUGAUGGACGUAUUACCGUUCAGAGAGCUGAGCUCCUGUUCG
AGAUUAAGGUGGCCAAAGGUAGUUCUUCCACGUCUUAUUAUGAAGCCUUGCAGCCGAC
CUUAACUCGAAUGACGUGGCUAAACUCUCUGAGUGGUAGGUCCUCUCAGAUAAUUUGG
CAUUCCGCGAGACUAGAGUAUCGCCCUGCUGUGGGGGCGAUGAAAGAUGGCUCUCUAG
UCGUCGGAGUCGACUGGGACCCUACGACGACCGUGCCUAGUAAAAGCAAGGUCCAAAG
UUGUACUCCUAACUUUCAGGUCCCGGUAUGGCAGAAGAAGGAGUUGACUCUCCCAGCG
UCUCGGUUGCAGAGUCGUAAGUAUUAUGCUCUCUCAGAUUCAGGGGAUAUACCCAUGA

UAGACAAAGCCCCUUAUGGGGUUCAGAUGUCUCAAAGUUGUUCCUCGAAUCCUUAUGA
GGAACAGUACUUUGGAGACAUCUGGAUCCACUAUAAGGUAACCCUGCAGGGGCCUAAA
UAGGGAGACGAGACGCCCCCCCCAUUACCUCCAGGACCUAGUGUGUUUGAUUACCACA
UGGUUCGGGAGGUAACCCUAUAUGAAGUGCGGGCCAUUGUAAGAACUAAAUAUGAGCC
CCAACAUAAACAGACUUCUGGAUAUUAUAAACCCUUUGAGAUCCAGAUGGCGAACAGG
GAUAUGAUAACGUAUGCAGCGGCGGCUGAGGUUUCAGUCAAUGGAACCGAUUUGACUA
UUGAGGAUAAUUAUAGCGUUUCUCACGAUGAUUCGAAAGUGGACAACGUGAGUGAAAU
UCAAUGCGCUUCAAGACCCCCGACAUGGAAUGUUGAACCUGUAGGAUUGGUGCACAUU
GGUUUAUCUUUGAAAAGUCCGUAUUUCCGAAGAUAGCGAUGUUUGAUUUUACGGUUC
AUUUCGUGUCAAAUAGGAACAUUUUUGUUCCUAAGGCUCUUAAAGAAGUGAACGUUGC
UAUUUCUGAGAGCGCGAAGAUCAAGGUUAUUGGUCCUGUAAUGAAUCUUUUCAACAAU
UCAGCUCCUAGGAGCGAUAAUUGGUUCAUGAUAAAGUUUUGUCAGUUGAUAGAAAUCU
UAGAUCCAACAGCGGUAAUAAAUGUUAACAUCGUCAUCAACCAAACCUAUGUGGAUUG
GUUGAUGAGGAUGGUGACAUGGUGUUGGAUAUUACGCCAAUAA

>Guadeloupe_mosquito_viruses_strain_AP27_segment1_MT808029
AUAGCUUUCUCACUGACAUAAGUGCCAGUGAGAAAGCUAUGCCGGGAAGUUCUUUUGA
GGUAGCUAAAGUCCUGCCUACUUUCCAUGGCGUGGUAAUGGCAUCGUUAGAUGGGAUU
ACUUAUCACGUUAUGGGCCAGUGUUUCUGGGUCGAUGAAGGCUUAGUGACAGCAGCCC
ACGUGAUUGAGGGUUUUGAAUAUUUGUGUAUCUACAAGGACGAAGAGAAACGCAUCGA
AGUAGGACCAAAAACAUUCAAAACCCUCAAUCACGUUUGCGCAGUAUUUCGCAAUCCA
ACACUGAUAUCGCAAAACUUUGCUUUGCUCAAACCCAAUUUUGCGAGGCUGGCGGCCG
CAAAAGAUGCUGGCUUGAGUGUUAACAUCACAGCCAUGGGAAAGAGAACGAUAGGAUU
CCUAGAUAGACAUCCACAAUUUGGGUAUGUUCGUUAUAGUGGAAGCACCAUUAAAGGA
UUUAGUGGAGCUCCUUACUAUUUUGGCAGGACCAUAUUUGGAAUGCAUCUGGGGUCGG
ACUCUAAGAACCUUGGUUAUGAUGGAGCAUUCUUGAAAACUGAUCUCCAAAAUAGUAA
GGCACUAAUGCUCCAAUUAAAUUUUAGAACUGAAGAUAGUGCAAGUUUUCUAGAGCUC
CAAUUAGACCUUCAUCAACAAAUACAUCCCUCCAAUCCUCCGUAUAUUCCUCGGGAAU
AUACGGUUAGGAUUGGAGGGAUGUAUCAUAUUGUUGAUGAGGAAGUGUUGAGUACCG
UCUUAAAGAGAAGAUCCGCUAAGCGAGCACAGGGGGAUUUACAAUAUUUGGCUGAACA
AGCUGAAGUUGGGCCCCUCAUUGAUCUCGAAGCUGUAACCACUAAGGGAGACCUUACU
AGGUCUCAACUUAGUGGUUACAGCUUCGAGAUCAUGAGGGGCCCAACUGAGACGGAUC
CGAUAAUAGCUAUGACUAAAGGGCUAUUGUCAGAAUUUGGCAAAACCUCAGUGUACGU
UCCUCCAGAACCUAAGAGUAAAGACCAAGGGACAUGCACUAGUUGUGAGUCUUGUGGC
GCCUAUAUAUAUUGGGGGGAGUCUAGGGAUAUUCCUGAAACGCCUACUGCGGAUGCCA
AGAAAGCGGUACAUUUACGCGUAGCUUCUUUUAGCGGAGCACGCGUCAAUGUACGGGA
AAACCUGAUCAGGGCUCCAGCUGUAGUUGUUGGGGCCCGUGGCAUGGAAUAUCAGAGG
GAAAGUGUACAGAGCCACGACUUGCCAAGAUGCAAGAAAAUGGAUUGCAUGUGCCAGA
AACCUUUGGAGACCUGCCUUAUGGAAUCCCGCACAUUGACGCGUGCUCCGCCAAAAGA
AGCUUCCGCGCGCACUGUCAGAAACAAAAACAGACGGAUGCUCCACCUCCAGCGGAAG
AGGUCCGUAGAGCUGCUCAAACUGCUCUACGGUGCUAUUCCAUAUCUAUCUCCUCUAG
UGCAUUUAUUGAAUUCCCUAUCAAACCAUUUGAUGGGGAAUUCAAUAAAGCACUAGAG
GAGAUAGAUAUGGAUAGCACUCCUGGCUACUGUUUUUUGACUCUUUUAGGCUCUACGA
AUGCGCAAAUCUUUGGUUGGAAUGGUAUAUGCUGUGAUCCGGAUAGAGUUAGUAUGG
UACGAGAGCAUGUCAAAUUAAGGUUCAAUGAGUUGUUGGCAGGUAAGGCUGUGAGCGA
UUGUAUUAAGGUUUUUGUGAAGCAAGAGCCGCAUAAAAUGAAGAAAAUUCAGGAAGG
UACUUAUCAAGCGAUAAGUACCUUCCUGAAUUUUCUUCAAACCUCUUCAAACAGUAGC
UUGAUAGUUUCAUCAGCCAGAGCUCAGAUGGAUUCCGUCGGGGAGACGCCAUGUCUCA
UCGGGUGGAGUCCAGUUAGAGGCGGAUGGCGUAAGAUAGUGAACAGGUUUGGCAACCA

AGCAGUAAAUUGUUUAGAUAGAUCGGCUUGGGAUUGGACUGUACAAGGCUAUUUAAU
UGAUCUUUGGAUAUUGUUUUUGGAAAAUUUACCAGUGAAUCCUCCAGACUGGUGGCUU
AAGAUGAUGAAACUACGUUUUAAGCUACUGUUUGAAGAGGUUUGGUUUCAGUUCGAA
GACGGGACAAGAGUAAAGCAGGCAACGCGAGGAGUAAUGAAGAGUGGUUGCUAUUUAA
CUAUUUUGUUAAAUAGUCUCAGCCAAAGUCUCUUACAUUACGUAGUCAACAGUAGAUG
UGGGAAGCCCAUGACUAUGAAACAACCAUACUCGAUAGGUGAUGACACGGUACAGGAA
GCUAUGGAUUGUUUCAUACUCAUCGUUAGACAACUCGAGCGAGUGGGAAUCACGGUCA
AAGGAGCGAAAGUGCAGCAUUGGGUGGAAUUGGCGGGGAUCUGUUUUGACGGGAAAAC
UUGUUAUCCGGCAUACUGGCAAAAACAUCUUUUUAACCUUAAGUAUACGCAAAAUCUU
GACCUAACUCACGAUCUACACACAAAUUCGUACGUCAAUGAGCCUGUGAUGUACGAAU
UUGUGUGUAGAGUGGCUCGUGAGUUAGGUCCUCGUUUCGUCUUGCCUAAGAUAGAAGC
ACUAGACAUAAUGAAUCAACCAAAUUGGUUCAUUAUGUCUAGUGCUUCUAUCUUAUCU
UGGGCAAGACGAAACGAGGACCUAACUCACGAGCCA

>Guadeloupe_mosquito_viruses_strain_AP27_segment2_MT808030
UAUUAUGCUCUCUCAGAUUCAGGGGAUAUACCCAUGUAUCCGAGAGAGCACGAUACUU
ACGACUCCGCAACCUAACGCUUCUGCUAAGAACCGGCGCAAUGGGCGCAAUAAGAAAC
GCGGAGGAGCCGCUGGCCCGAGCGGGGUUUCUGCUGAUGGACGUAUUACCGUUCAGAG
AGCUGAGCUCCUGUUCGAGAUUAAGGUGGCCAAAGGUAGUUCUUCCACGUCUUAUUAU
GAAGCCUUGCAGCCGACCUCGACCCGAAUGCCGUGGCAAAUUACUCUGAGAGAAACUA
CCACUCAGAUAAUUUGGCAUUCCGCGAGACUAGAGUAUCGCCCUGCUGUGGGGGCGAU
GAAAGAUGGCUCUCUAGUCGUCGGAGUCGACUGGGACCCUACGACGACCGUGCCUAGU
AAAAGCAAGGUCCAAAGUUGUACUCCUAACUUUCAGGUCCCGGUAUGGCAGAAGAAGG
AGUUGACUCUCCCAGCGUCUCGGUUGCAGAGUCGAAGUUAUUCUGCCAUCCCAGAUUC
AGGGGGUAUAUUUAGGCCCCGCAGGGUUCCUUAUAGUGGAUCCAGAUGUCUGUACUGU
UCCUCGAAUAAGGAUGAGGAACAGUACUUUGGAGACAUCUGGAUCCACUAUAAGGUAA
CCCUGCAGGGGCCUAAAUAGGGAGACGAGACGCCCCCCCAUUACCUCCAGGACCUAGU
GUGUUUGAUUACCACAUGGUCCGGGAGGUGACCCUAUAUGAAGUGCGGGCCAUUGUAA
GAACUAAAUAUGAGCCCCAACAUAAACAGACUUCUGGAUAUUAUAAACCUUUUGAGAU
CCAGAUGGCGAACAGGGAUAUGAUAACGUAUGCAGCGUCGGCCGACUUUCGAAUCAUC
GUGAGAAACGCUAUAAUUAUUGUCGAUAGUUAUAGCGUUUCUCACGAUGAUUCGAAAG
UGGACAACGUGAGUGAAAUUCAAUGCGCUUCGAGACCCCCGACAUGGAAUGUUGAACC
UGUAGGAUUGGUGCACAUUGGUUUAUCUUUGAAAAGUCCGUAUUUUCCGAUCAUCGCG
AUCUCAGAAAUAGCAGUUCACUUCUUUAAGAGCCUUAGGAACAAAGAUGUUCCUAAGG
CUCUUAAAGAAGUGAACGUUGCUAUUUCUGAGAGCGCGAAGAUCAAGGUUAUUGGUCC
UGUAAUGAAUCUUUUCAAUAGUCAAGCUCCUAGGAGCGAUAAUUGGUUCAUGAUAAAG
UUUUGUCAGUUGAUAGAAAUCUUAGAUCCAACAGCGGUAAUAAAUGUUAACAUCGUCA
UCAACCAAUCCUCAUAUGGAUUGGUUGAUGACGAUGUUAACAUUUAUUACCGCUGUUG
GAUCUAAGA

>Guadeloupe_mosquito_viruses_strain_AP29_segment1_MT808031
CUUUGUUUGCUCAAACCCAUUUUUGGUUAUCAGUUUUGGAUUACGCAGUAUGUCGCCA
AUCCAACGACUGAUAACGCAAAAAUUGGGUUUGAGCAAAGCAAAGUUUGCGAGGCUGG
CGGUCGAAAAAGAUGCCGGCUUGAGUGUUAACAUCACAGCCAUGGGAAAGAGAACGAU
AGGAUUCCUAGAUAGACAUCCACAAUUUGGGUAUGUUCGUUAUAGUGGAAGCACCAUU
AAAGGAUUUAGUGGAGCUCCUUACUAUUUUGGCAGGACCAUAUUUGGAAUGCAUCUGG
GAUCCUAUUCUAUGGUCCUGAAAUGUAAGGAGCUCCACCAGAAAACUGAACUCAAGCC
CAGUCGGGUUAUAAGAAACCAGUUAAAUUUAAGAACUGAAGAUAGUGCAAGUUUUCUA
GAGCAGCAGUUAGAGCGUUAUGAAGAAGUAGAGUAUUCUAGAUCUCCGUAUAAUCCCG

AUGAAUAUAAGGUUAGGAUUGGAGGGAUGUAUCAUAUUGUUGAUGAGGAAGUGUUGA
GUACCGUCUUAAAGAGAAGAUCCGCUAAGCGAGCACAGGGGGAUUUACAAUAUUUGGC
UGAACAAGCUGAAGUUGGGCCCCUCAUUGAUCUCGAAGCUGUAACCACUAAGGGAGAC
CUUACUAGGUCUCAACAGAGUGGACAAAAACAGCACCAGGAGCUUUCUACUGAGACGG
AUCCGAUAAUAGCUAUGACUAAAGGGCUAUUGUCAGAAUUUGGCAAAACCUCAGUGUA
CGUUCCUCCAGAACCUAAGAGUAAAGACCAAGGGACAUGCACUAGUUGUGAGUCUUGU
GGCGCCUAUAUAUAUUGGGGGAGUCUAGGGAUAUUCCUGAAACGCCUACUGCGGAUG
CCAAGAAAGCGGAAGAUUUACCUUUAGUGCCGAGGGAUGCUAUGACGUUUAAUGAUUC
GGAAAACCUGAUCAGGGCUCCAGCUGUAGUUGUUGGGGCCCGUGGCAUGGAAUAUCAG
AGGGAAAGUGUACAGAGCCACGACUUGCCAAGAUGCAAGAAAAUGGAUUGCAUGUGCC
AGAAACCUUUGGAGACCUGCCUUAUGGAAUCCCGUACAUUGACGCGUGCUCCGCCAAA
AGAAGCUUCCGCACGCACUGUCAGAAACAAAAACAGACGGAUGCGCCGCCAACAGCAG
AAGAAAGAACUAGAGCAGUUCAAGCAGCUCUACGGACCUAUUCCGCAUGGAGGUGUGA
CCUUACAUCCGCAGCAGAUCCCCACAAAUGGUUUGACAGGGAGUUCAAUAAAGCACUA
GAGGAGAUAGAUAUGGAUAGCACUCCUGGCUACUGUAUUUUGACUCUUUUAGGCUCUA
CGAAUGCGCAAAUCUUUGGUUGGAAUGGUAUAUGCUGUGAUCCGGAUAGAGUUAGUAU
GGUACGAGAGCAUGUCAAAUUAAGGUUCAAUGAGUUGUUGGCAGCUUCGUCUAUAAUC
UAUCGGAUCAAGAUAUUUGUGAAGCAAGAGCUGCAUCCAACGAACAAAAUUCUGUCAA
AAGGUACUUAUCGCUUGAUAAGCGCUGUUAGCUUAAUUGAUACCUUUAUUGACAGAAU
UUUGUUCGCUUGGAUAGCCAGAGCUCAGAUGGAUUCCGUCGGGGAGACGCCAUGUCUC
AUCGGGUGGAGUCCAGUUAGAGGCGGAUGGCGUAAGAUAGUGAACAGGUUUGGCAACC
AAGCAGUAAAUUGUUUAGAUAGAUCGGCUUGGGAUUGGACUGUACAAGGCUAUUUAA
UUGAUCUUUGGAUAUUGUUUUUGGAAAAUUUACCAGUGAAUCCUCCAGACUGGUGGCU
UAAGAUGAUGAAACUACGUUUUAAGCUACUGUUUGAAGAGGUUUGGUUUCAGUUCGA
AGACGGGACAAGAGUAAAGCAGGCAACGCGAGGAGUAAUGAAGAGUGGUUGCUAUUUA
ACUAUUUUGUUAAAUAGUCUCAGCCAAAGUCUCUUACAUUACGUAGUCAACAGUAGUU
GUGGGAAGCUCAUGACUAUGAAACAACCAUACCAGAUAGGUGAUGACACGGUACAGGA
AGCUAUGGAGUAUGGCUUGUUUUAUACUACGUUAAGCAACUCGAGAUAUUGGGAAUCA
CGGUCAAAGGAGCUAAAGUCCAGCAUUGGGUUGAAUUCGCGGGGUUCUGUUUUGACGG
GAAAACUUGUUAUCCGGCAUACUGGCAAAAACAUCUUUUUAACCUUAAGUAUACGCAA
AAUCUUGGGGAGACUUUGCGUAGUCUACACACAAAUUCGUACAUCACAGAGCUCAUUG
ACGUACGAAUUUGUGUGUAGAGUGGCUCGUGAGUUAGGUCCUCGUUUCGUCUUGCCUA
AGAUAGAAGCACUUGACAUAAGGAAUCAACCAAAUUGAUUCACUAUGUCAAGUUCGCU
UCUAUCUUAUGGCAAGACGAAACG

>Guadeloupe_mosquito_viruses_strain_AP29_segment2_MT808032
UAUCUCAAGCAGCGGAAUCCACCUUUUAUUGUCUACACACCAGCGCUCGGGCCAGCGGC
UUCCUCCACGUCUUCUGCUAAGAAACGGCGCAAUAGGCGCAAUAAGAAACGUGGAGGA
GCCGCUGGCCCGAGCGGGGUUUCUGCUGAUGGACGUAUUACCGUUCAGAGAGCUGAGC
UCCUGUUCGAGAUUAAGGUGGCCAAAGGUAGUUCUUCCACGUCUUAUUAUGAAGCCUU
GCAGCCGACCUCGACUCGAAUGACGUGGCUAAAGACUCUGGGCGAUACUUACUCUCGG
AUAAUUUGGCAUUCCGCGAGACUAGAGUAUCGCCCUGCUGUGGGGGCGAUGAAAGAUG
GCUCUCUAGUCGUCGGAGUCGACUGGGACCCUACGACGACCGUGCCUAGUAAAAGCAA
GGUCCAAAGUUGUACUCCUAACUUUCAGGUCCCGGUAUGGCAGCAUAAGUACUUGACU
CUGCAACCGAGACGCUGGGAGAGUCGUAAGUAUUAUGCUCUCUCAGAUUCAGGGGAUA
UACCCAUGAUAGACAAAGCCCCUUGUGAGGUUUUGGCCUAUUUGGCUUGUUCCUCGAA
UAAGGAUGAGGAACAGUACUUUGGAGACAUCUGGAUCCACUAUAAGGUAACCCUGCAG
GGGCCUAAAUAGGGAGUCCAGAUGCUAUCCAAAGUACCUCCAGGACCUAGUGUGUUUG
AUUAUCACAUGGUUCGGGAGGUAACCCUAUAUGAAGUGCGGGCCAUUGUAAGAACUAA

AUAUGAGCCCCAACAUAAACAGACUUCUGGAUAUUAUAAACCCUUUGAGAUCCAGAUG
GCGAACAGGGAUAUGAUAACGUAUGCAGCGGCGGCUGAGGUUUUAGUCAAUGGAACCG
AUUUAACUAUUGAGGAUAAUUAUAGCGUUUCUCACGAUGAUUCGAAAGUGGACAACGU
GAGCGAAAUUCAAUGCGCUUCGAGACCCCCGACAUGGAAUGUUGAACCUGUAGGAUUG
GUGCACAUUGGUUUAUCUUUGAAAAGUCCGUAUUUUCCAAAGAUAGCGAUGUUUGAUU
UUACGGUUCAUUUCGUGUCGAAUAGGAACGUUCACUUCUUUAAACGCCUUAGGAACAA
AGUUGUUCCUAUUUGUCACGAAAUGAACCGUAAAAUCUCUGAUAUUGAAAAGAUUCAG
GUUAUCGGUACUGUAAUGAAUCUUUUCAAUAGUCAAGCUCCUAGGAGCGAUAAUUGGU
UCAUGAUAAAGUUUUGUCAGUUGAUAGAAAUCUUAGAUCCAACAGCGGUAAUAAAUGU
UAACAUCGUCAUCAACCAAACCUAUGUGGAUUGCGAUGAUGAGGAUGGUGACAUGGUG
UUAGCGAUCUUAAGAUUUCUAU

>Guadeloupe_mosquito_viruses_strain_AP30_segment1_MT808033
CAUAGCUUUCUACUGCAUAAGUGCCAAGCUCCAGCUGGCACUUAUGUCAGUGAGAAAG
CUAUGCCGGGAAGUUCUUUUGAGGUAGCUAAAGUCCUGCCUACUUUCCAGGCUAUGGU
AAUGCCAUCGUUAGAUGCCAUAACUUAUCACGUCAUGGGCCAGUGUUUCUGGGUCGAU
GAAGGCUUAGUGACAGCAGCCCACGUGAUUGAGGGUUUUGAAUAUUUGUGUAUCUACA
AGGACGAAGAGAAACGCAUCGAAGUAGGACCAAAAACAUUCAAAACCCUCAAUGGGGA
UUGCGCAGUAAGUCGUAAUCCAACACUGAUAACGCAAAAAUUGGGUUUGAGCAAAGCA
AAGUUUGCGAGGCUGGCGGUCGAAAAAGAUGCCGGCUUGAGUGUUAACAUCACAGCCA
UGGGAAAGAGAACGAUAGGAUUCCUAGAUAGACAUCCACAAUUUGGGUAUGUUCGUUA
UAGUGGAAGCACCAUUAAAGGAUUUAGUGGAGCUCCUUACUAUUUUGGCAGGACCAUA
UUUGGAAUGCAUCUGGGGUCGGACUCUAAGAACCUUGGCACUGAUGCUUCAUUCUUGA
AAACUGAACUCAAGCCCAGUCGGGUUAUAAGAAACCAGUUAAAUUUAAGAACUGAAGA
UAGUGCAAGUUUUCUAGAGCAGCAGUUAGAGCGUUAUGAAGAAGUAGAGUAUUCUAG
AUCUCCGUAUAAUCCCGAUGAAUAUAAGGUUAGGAUUGGAGGGAUGUAUCAUAUUGUU
GAUGAGGAAGUGUUGAGUACCGUCUUAAAGAGAAGAUCCGCUAAGCGAGCACAGGGGG
AUUUACAAUAUUUGGCUGAACAAGCUGAAGUUGGGCCCCUCAUUGAUCUCGAAGCUGU
AACCACUAAGGGAGACGUGACUAGGUCUCUACAGAGUGGACAAAAACAGCACCAGGAG
CUUUCUACUGAGACGGAUCCGAUAAUAGCUAUGACUAAAGGGCUAUUGUCAGAAUUUG
GCAAAACCUCAGUGUACGUUCCUCCAGAACCUAAGAGUAAAGACCAAGGGACAUGCAC
UAGUUGUGAGUCUUGUGGCGCCUAUAUAUAUUGGGGGGCGUCUAGAGAUAUUCCUGAA
ACGCCUACUGCGGAUGCCAAGAAAGCGGAAGAUUUACCUUUAGCGCCUAGGAAUGCCA
UGACGUUUAAUGAUUCGGAAAACCUGAUCAGGGCUCCAGCUGUAGUUGUUGGGGCCCG
UGGCAUGGAACAUCAGAGCGCAAGCGUACACACCCACCACUUGCCAAGAUGCAACAAA
AUCGAUUGCAUGUGCCAGAAACCUUUGGAGACCUGCCUUAUGGACUCCCGUACAUUGA
CGCGUGCUCCGCCAAAAGAAGCUUCCGCACGCACUGUCAGAAACAAAAACAGACGGAU
GCGCCGCCAACAGCAGAAGAAAGAACUAGAGCAGUUCAAGCAGCUCUACGGACCUAUU
CCGCAUGGAGGUGUGACCUUACAUCCGCAGCAGAUCCCCACAAAUGGUUUGACAGGGA
AUUCAAUAAAGCACUAGAGGAGAUAGAUAUGGAUAGCACUCCUGUCUACUGUAAUUUG
ACUCUUAUAGGUACUACGAAUGCGCGAAUCUUUGGUUCGAAUGGUAUAUGCUGUGAUC
CGGAUAGAGUUAGUAUGGUACGAGAGCAUGUCAAAUUAAGGUUCAAUGAGUUGUUGG
CAGGUAAGGCUGUGAGCGAUUGUAUUAAGGUUUUUGUGAAGCAAGAGCCGCAUAAAAU
GAAGAAAAUUCAGGAAGGUACUUAUCGCUUGAUAAGUACCGUUAGCUGGAUUGUUCGC
UUGAUAGCCAGAAGAUGGUUCGCGGGGAGAGCCAGCUCUCGGGUGGAGUCCGUCGAGG
AGACGCCAUGUCUCAUCGGGUGGAGUCCAGUUAGAGGCGGAUGGCGUAAGAUAGUGAA
CAGGUUUGGCAACCAAGCAGUAAAUUGUUUAGAUAGAUCGGCUUGGGAUUGGACUGUA
CUAUCUUACGUAGUUGAUCUUUGGAUAUUGUUUUUGGAAAAUUUACAAACCAAACCUC
CAGACUGGUGGCUUAAGAUGAUGAAACUACGUUUUAAGCUACUGUUUGAAGAGGUUUG

GUUUCAGUUCGAAGACGGGACAAGAGUAAAGCAGGCAACGCGAGGAGUAAUGAAGAGU
GGUUGCUAUUUAACUAUUUUGUUAAAUAGUCUCAGCCAAAGUCUCUUACAUUACGUAG
UCAACAGUAGAUGUGGGAAGCCCAUGACUAUGAAACAACCAUACUCGAUAGGUGAUGA
CACGGUACAGGAAGCUAUGGAUUGGUUGUUUCUAGACUACGUUAGACAACUCGAGAUA
UUGGGAAUCACGGUCAAAGGAGCUAAAGUCCAGCAUUGGGUUGAGUUCGCGGGGUUCU
GCUUUGACGGAAAAAGAUGUUUUCCAGUAUACAGGCUCAUUGAUCGUACAAAACUGAU
AACUACGCAAAGUCUCCCGGAGACUUUGCGUAGUUAUCAGUAUUUGUACGUCAAUGAG
CCUGUGAUGUACGAAUUUGUGUGUAGAGUGGCUCGUGAGUUAGGUCCUCGUUUCGUCU
UGCCUAAGAUAGAAGCACUAGACAUAAUGAAUCAACCAAAUUGAUCCUAUGUCAAGUU
CUCUUCUAUCUAAUGGUGAGUGAAACGGGAUGAGUUGGCAAACAAUAGUGGGA

>Guadeloupe_mosquito_viruses_strain_AP30_segment2_MT808034
ACCCCUUUAACUGCUCAAGUAGCUGCGAUGAAGGUUGCUAAACCGCAACCAAACGCUU
CUGCUAAGAAAGGGCGCAAUAGGCGCAAUAAGAAACGGGGAGGAGCCUCUAGUCCGAG
CGGUGUGUCUGCUGAUGGACGUAAUACCGUUCAGAGAGCUGAGCUCCUGUUCGAGAUU
AAGGUGGCCAAAGGUAGUUCUUCCACGUCUUAUUAUGAAGCCUUGCAGCCGACCUCGA
CCCGAAUGACGUGGCUAAAGACUUUGGGUGGUAGUUUCUCUCAUAUAAUGUGGCAUUC
CGCGAGACUAGAGUAUCGCCCUGCUGUGGCGGCGAUGAAACAUGGCUCUCUAGUCGUC
GGAGUCGACUGGGACCCUACGACGACCGUGCCUAGUAAAAGGAAGGUACAAAGUUGUA
CUCCCAACUUUCAGGUCCCGGUAUCUGAGAAGAAAUACUUGACUCUGCAACCGUGUCG
GUUGCAGAGUCGUAAGUAUUAUGCUCUCUCAGAUUCAGGGGAUAUACCCAUGAUAGAC
AAAGCCCCUUGUGAGGUUUUGGCCUAUUUGGCUUGUUCCUCGAAUAAGGAUGAGGAAC
AGUACUUUGGAGACAUCUGGAUCCACUAUAAGGUAACCCUGCAGGGGCCUAAAUAGGG
AGACGAGACGCCCCCCCAUUACCUCCAGGACCUAGUGUGUUUGAUUACCACAUGGUUC
GGGAGGUAACCCUAUAUGAAGUGCGGGCCAUUGUAAGAACUAAAUAUGAGCCCCAACA
UAAACAGACUUCUGGAUAUUAUAAACCCUUUGAGAUCCAGAUGGCGAACAGGGAUAUG
AUAACGUAUGCAGCGGCGGCUGAGGUUUUAGUCAAUGGAACCGAUUUAACUAUUGGGG
GUAAUAAGAGCGUUUCUCAAGUUGAUUCCACUUUGAACAUUGUGAUUGAAAUUCAAUG
CGCUUCAAGACCCCCGAAAACCAAUGUUGAACCUGUAGGAUUGGUGCACAUUGGUUUA
UCUUUGAAAAGUCCGUAUUUUCCGAAGAUAGCGAUGUUUGAUUUUACGGUUCAUUUCG
UGUCAAAUAGGAACAUUUUUGUUCCUACGGCACUUAAGGAAGUGAACGUAGCGAUUUC
UAUGAACGCGAAAAUCAACAUCGCUAUUUCUAUAAUGAAUUUUCAUGACAAUUCAGCU
CCUAGGAGCGAUAAUUGGUUCAAGAUUAAGUUUUGUCAGUUGUUGGAAAUCUUGGAUC
CAACAGCGGUAAUAAAUGUUAACAUCGUCAUCAACCAAACCUAUGUGGAUUGGUUGAU
GGCGCAUCAUUACAUGGUAUUGGAUAUUACGCCAAUAAUUACGUUCUAUAACUCUGUC
ACGUCUCUGUUGCACUUUAGGGAGGGGGAGGGUGAGAGUUCCGCAGCGAACAGUAGCG
AGUCGCUUGAAAGUUAUGAGGAAUCCCCAUCCUCAAGUGUUUUAAGCGAUGAGUUUCA
AGUUGUGGACCACUAUUAGUUUGCCAACGUGUCGUUAAGUGCUCGACAGUAACUAAUA
UUGGUCCUGUACACUGAAUCUUUUCAAUAGUCAAGCUCCUAGGAGCGAUAAUUGGUUC
AUGAUAAAGUUUUGUCAGUUGAUAGAAAUCUUAGAUCCAACAGCGGUAAUAAAUGUU
AACAU

>Guadeloupe_mosquito_viruses_strain_AP33_segment1_MT808035
CCCAUCUAACGAUGCCAUUACAUAGCCUGGAAAUUAGGCAGGACUUUAGCUACCUCAA
AAGAACUUCCCGGGGAAASGAGCAUAGCUUUCUCACUGUACAUAAUGGCCAAGCUGGA
GCUGGCACUUAUGUCAGUGAGAAAGCUAUGCCGGGAAGUUCUUUUGAGGUAGCUAAAG
UCCUGCCUACUUUCCAGGCUAUGGUAAUGGCAUCGUUAGAUGGGAUUACUUAUCACGU
UAUGGGCCAGUGUUUCUGGGUCGAUGAAGGCUUAGUGACAGCAGCCCACGUGAUUGAG
GGUUUUGAAUAUUUGUGUAUCUACAAGGAUGAAGAGAAACGCAUCGAAGUAGGACCAA

AAACAUUCAAAACCCUCAAUCACGUGGGCUGCUCACUAAGCCGUAAUCCAACACUCAA
GCCGGCAUCUUUUUCGUUCUGCCAAGCAAAGGUUUCUUAUAACCCGACUGGGCUUGAG
UUCAGUUUGAAUGCUCACAUCACAGCCAUGGGAAAGAGAACGAUAGGAUUCCUAGAUA
GACAUCCACAAUUUGGGUAUGUUCGUUAUAGUGGAAGCACCAUUAAAGGAUUUAGUGG
AGCUCCUUACUAUUUUGGCAGGACCAUAUUUGGAAUGCAUCUGGGGUCGGACUCUAAG
AACCUUGGUUAUGAUGGAGCAUUCUUGAAAACUGAACUCAAGCCCAGUCGGGUUAUAA
GAAACCAGAUACAUCUWAGAACUGAAGAUAGUGCAAGUUUUCUAGAGCAGCAGUUAGA
GCGUUAUGAAGAAGUAGAGUAUUCUAGAUCUCCGUAUAAUCCCGAUGAAUAUAAGGUU
AGGAUUGGAGGGAUGUAUCGCAGGGUUAAUGAAGAAGUAGAGUAUAUCUCCGUAAAG
AGAAGAUCCGCUAAGCGAGCACAGGGGGAUCUUCUCUUUAAGACUGUACAAGCUGAAG
UUGGGCCCCUCAUUGAUCUCGAAGCUGUAACCACUAAGGGAGACCUUACUAGGUCUCA
ACAGAGUGGACAAAAACAGCACCAGGAGCUUUCUACUGAGACGGAUCCGAUAAUAGCU
AUGACUAAAGGGCUAUUGUCAGAAUUUGGCAAAACCUCAGUGUACGUUCCUCCAGAAC
CUAAGAGUAAAGACCAAGGGACAUGCACUAGUUGUGAGUCUUGUGGCGCCUAUAUAUA
UUGGGGGGAGUCUAGGGAUAUUCCUGCAACAACUACAGCUGGAGCCCUGAUCGCGGAA
GAUUUACCUUUAGCGCCUAGGAAUGCCAUGACGUUUAAUGAUUCGGAAAACCUGAUCA
GGGCUCCAGCUGUAGUUGUUGGGGCCCGUGGCAUGGAAUAUCAGAGGGAAAGUGUACA
GAGCCACGACUUGCGGAGAUGCGUCAAUGUACGGGAUUGCAUAAGGCAGGUCUCUUUA
GGUUUCUGCCAUAUGCAAUCCCGUACAUUGACGCGUGCUCCGCUAAAAGAAGCUUCCG
CACGCACUGUCAGAAACAAAAACAGACGGAUGCGCCGCCAACAGCAGAAGAAAGAACU
AGAGCAGUUCAAGCAGCUCUACUUCUUUCUUCCGCUUGGAGGUGCGACCUCACAUUCG
CUGCAGAUCCCCACAUUCGUAGAGCUAAAAGAGUCAAUAAAGCACUAGAGGAGAUAGA
UAUGGAUAGCACUCCUGGCUACUGUAUUUUGACUCUUUUAGGCUCUACGAAUGCGCAA
AUCUUCGGCUGGAAUGUGAUAUGCUGUGAUCCGGAUAGAGUUAGUAUGGUACGAGAGC
AUGUCAAAUUAAGGUUCAAUGAGUUGUUGGCAGGUAAGGCUGUGAGCGAUUGUAUUA
AGGUUUUUGUGCAGCAAGAGCUUCAUAAAAUGAAGAAAAUUCAGGAAGGUACUUAUCG
CUUGAUAAGCGCUGUAAGCUUAAUUGAUACCUUUAUUGACAGAAUUUUGUUCGCUUGG
AUAGCCAGAGCUCAGAUGGAUUCCGUCGGGGAGACGCCAUGUCUCAUCGGGUGGAGUC
CAGUUAGAGGCGGAUGGCGUCAGAUACGGAAUCCAUCUGAGCUCAAGCUAGUCAAUUG
UUUAGACAGAUCAGCUUGGAAUUGGACUGUACAAGGCUAUUUAUUUGAUCUUAGGACA
UCGUAUCUGGAAAAUUUACCAGUGAAUCCUCCAGACUGGUGGCUUAAGAUGAUGAAUC
UACAUUUUAAGCUACUGUUUGAAGAGGUUUGGUUUCAGUUCGAAGACGGAACAAGAGU
AAAGCAGGCAACGCGAGGAGUAAUGAAGAGUGGUUGCUAUUAUACUAUUUUGUUAAA
UAGUCUUAGCCAAAGUCUCUUACAUUACGUAGUCAACAGUAGAUGUGGGAAGCCCAUG
ACCAUGAAACAACCAUACUCGAUAGGUGAUGACACGGUACAUGAGCUAUCGGAUUCCC
UUCUAGACUAUGUUAGACAACUCGAGAUAUUGGGAAUCACGGUCAAAGGAGCUAAAGU
CCAGCAUUGGGUUGAAUUUGCGGGGUUCUGUUUUGACGGGAAAACUUGUUAUCCGGUC
AAAACAGAACCCCGCAAAUUCAAUCUUAGGCAAGACGAAACGAGGACCGAACUCACGA
GCCACUCUACACAUUCGUACGUCAAUGAGCCUGUGAUGUACGAAUUUGUGUGUAGAGU
GGCUCGUGAGUUAGGUCCUCGUUUCGUCUUGCCUAAGAUAGAAGCACUAGACAUAAUG
AAUCAACCAAAUUGAUCCUAUGUCUAGUGCUUCUAUCUUAGGCAAGACGAAACGAGGA
CCUAACUCACGAGCCACUCUACACACAAAUUCGUACAUCA

>Guadeloupe_mosquito_viruses_strain_AP35_segment1_MT808036
GGCACUGACACUAAUGCCAGUGAGAAAGCUAUGCCGGGAAGUUCUUUUGAGGUAGCUA
AAGUCCUGCCUACUUUCCAGGCUAUGGUAAUGGCAUCGUUAGAUGGGAUUACUUAUCA
CGUUAUGGGCCAGUGUUUCUGGGUCGAUGAAGGCUUAGUGACAGCAGCCCAUAACGUG
AUUGAGGGUUUUGAAUAUUUGUGUAUCUACAAGGACGAAGAGAAACGCAUCGAAGUA
GGACCAAAAACAUUCGAGAUCGGCAAUGGGGAUUACGCAGUAUGUCGCAAUCCACCAU

UGCCGACUCGAAUCUUUGGUUUGCUCAAAGCCCAAUUUUUGCGUUAUGCAGUCGAACU
UGAGUUCAGUUUGAGUGUUAACAUCAUAGCCAUGGGAAAGAGAACGAUAGGAUUCCUA
GAUAGACAUCCACAAUUUGGGUAUGUUCGUUAUAGUGGAAGCACCAUUAAAGGAUUUA
GUGGAGCUCCUUACUAUUUUGGCAGGACCAUAUUUGGAAUGCAUCUGGGGUCGGACUC
UAAGAACCUUGGUUAUGAUGGAGCAUUCUUGAAAACUGAACUCAAGCCCAGUCGGGUU
AUAAGAAUGCUCCAGUCAAAACCAAGGUUCUUAGAGUCCGACGCCAGUUUUCUAGAGC
AGCAGUUAGAGCGUUCUUCAAAAAUACAGUCCUCCAGAUCCCCGUAUAUUCCCGAUGA
AUAUAAGGUUAGGAUUGGAGGGAUGUAUCAUAUUGUUGAUGAGGAAGUGUUGAGUAC
CGUCUUAAAGAGAAGAUCCGCUAAGCGAGCACAGGGGGAUUUACAAUUUUUGACGGUA
CAAGCUGAAGUUGGGCCCCUCAUUGAUCUCGAAGCUGUAACCACUAAGGGCGACCUUA
CUAGGUCUCAACAGAGUGGACAAAAACAGCACCAGGAGCUUUCUACUGAGACGGAUCC
GAUAAUCUCCAUGACUUACUGUCUAUUGUCAGAAUUUUGCAAAACCUCAGUGUACGUU
CCUCCAGAACCUAAGAGUAAAGACCAAGGGACAUGCACUAGUUGUGAGUCUUGUGGCG
CCUAUAUAUAUUGGGGGGAGUCUAGGGAUAUUCCUGAAACGCCUACUGCGGAUGCCAA
GAAAGCGGAAGAUUUACCUUUAGCGCCUAGGAAUGCCAUGACGUUUAAUGAUUCGGAA
AACCUGAUCAGGGCUCCAGCUGUAGUUGUUGGGGCCCGUGGCAUGGAAUAUCAGAGGC
AAAGUGUACAGAACCACUGGCGCUGGCGACGAUGCAAGAAAAUGGAUUGCAUGUGCCA
GAAACCUUUGGAGACCUGCCUUAUGGAAUCCCGUACAUUGACGCGUGCUCCGCCAAAA
GAAGCUUCCGCACGCACUGUCAGAAACAAAAACAGACGGAUGCGCCGCCAACAGCAGA
AGAAAGAACUAGAGCAGUUCAAGCAGCUCUACGGACCUAUUCCGCAUGGAGGUGUGAC
CUCACAUCCGCAGCAGAUCCCCACAAAUGGUUUGAUAGGGAAUUCAAUAAAGCACUAG
AGGAGAUAGAUAUGGAUAGCACUCCUGGCUACUGUAUUUUGACAAAUAAAGUAGCCAG
GAAGACGCAAAUCUUUGGUUCGAAUGGUAUAUGCUGUGAUCCGGAUAGAGUUAGUAUG
GUACGAGAGCAUGUCAAAUUAAGGUUCAAUGAGUUGUUGGCAGGUAAGGCUGUGAGCG
AUUGUAUUAAGGUUUUUGUGAAGCAAGAGCCGCAUAAAAUGAAGAAAAUUCAGGAAG
GUACUUAUCGCUUGAUAAGCGCUGUUAGCUUAAUUGAUACCUUUAUUGACAGAAUUUU
GUUCGCUUGGAUAGCCAGAGCUCAGAUGGAUUCCGUCGGGGAGACGCCAUGUCUCAUC
GGGUGGAGUCCAGUUAGAGGAGGAUGGCGUAAGACAGUGAACAGGUUUGGCAAACAAG
CAGUAAAUUGUUUAGAUCGAUCGGCUUGGGAUUGGCCUGUACAAGGCUAUUUAAUUGA
UCUUAAGACAUUGAUUUUGGAAAAUUUACCAGUGAAUCCUCCAGACUGGUGGCUUAAG
AUGAUGAAACUACGUUUUAAGCUACUGUUUGAAGAGGUUUGGUUUCAGUUCGAAGACG
GGACAAGAGUAAAGCAGGCAACGCGAGGAGUAAUGAAGAGUGGUUGCUAUUUAACAAA
UUUGUUAAAUAGUCUUAACCAAACUCUCUUACAUCGCGUUGCCUGCUUUAGAUGUGGG
AAGCCCAUGACCAUGAAACAACCAUACUCGAUAGGUGAUGACACGGUACAGGAAGCUA
UGGAUUGGCUUCUAGACUAUGUUAGGCAACUCGAGAUAUUGGGAAUCACGGUCAAAGG
AGCUAAAGUCCAGCAUUGGGUUGAAUUCGCGGGGUUCUGUUUUGACGGGAAAACUUGU
UAUCCGGCAUACUGGCAAAAACAUCUUUUUAACCUUAAGUAUACGCAAAAACGAGGCC
UAACUACGAGCUACUUAACAAAAUUCGUACGUCAAUGAGCCUGUGAUGUACGAAUUUG
UGUGUAGAGUGGCUCGUGAGUUAGGUCCUCGUUUCGUCUUGCCUAAGAUAGAAGCACU
UGACAUAAGGAAUCAAUUUGGUUGAUUCAUUAUGUCAAGUGCUUCUAU

>Guadeloupe_mosquito_viruses_strain_AP35_segment2_MT808037
GGUCAAGGAGGUGUGAAGAGACGGGCUCGGGCCAGAGGCCAACUUCACGCUUCUGCUA
AGAAACGGCGCAAUAGGCGCAAUAAGAAACGUGGAGGAGCCACUGGCCCGAGCGGGGU
UUCUGCUGAUGGACGUAUUACCGUUCAGAGAGCUGAGCUCCUGUUCGAGAUUAAGGUG
GCCAAAGGUAGCUCUUCCACGUCUUAUUAUGAAGCCUUGCAGCCGACCUCGACCCGAA
UGACGUGGCCAAAUACUCUGAGUGGUAGUUUCUCUCAGAUAAUUUGGCAUUCCGCGAG
ACUAGAGUAUCGCCCUGCUGUGGGGGCGAUGAAAGAUGGCUCUCUAGUCGUCGGAGUC
GACUGGGACCCUACGACGACCGUGCCUAGUAAAAGCAAGGUCGUCGUAGGGUCCAGUC

CUAACUUUCAGGUCCCAGUAUGGCAGAAGAAGGAGUUGACUCUCCCAGCGUCUCGGUU
GCAGAGUCGUAAGUAUUAUGCUCUCUCAGAUUCAGGGGACAUACCUCUCACAGACUGU
UCCCAUGAGUCAGAUGUCUCCAAAGUGACUUGUUCCUCGAAUAAGGAUGAGGAACAGU
ACUUUGGAGACAUCUGGAUCCACUAUAAGGUAACCCUGCAGGGGCCUAAAUAGGGAGA
CGAGACGCCCCCCCAUUACCUCAAACACCUAGUGUGUUUGAUUACCACAUGGUUCGGG
AGGUAACCCUAUAUGAAGUGCGGGCAUUUGUAAAAAAUAAAUAUGAGCGCCAACAUAA
ACAGCCUACUGGAUGUCAUAAGUGUUUUAAAAAUCAGAUGCCGAAGACGCCACAUAGG
GCUCUCAUACCGUCUCCAGCGGCCGCUGAGGCUUCCAUAAAUGCAAACAAAUUCACUA
UUGACGAUAAUUAUAGCGUGUCUCACGAUGAUUCCAAAGGGGUCAACGUGAGGGAAAU
UCAAUGCGCUUCAAUAUACCUACCGACAUGGAAUGUUGAACCUGUAGGAUUGGUGCAC
AUUGGUUUAUCUUUGAAAAGUCCGUAUUUUCCGAAGAUAGCGAUGUUUGAUUUUACGG
UUCAUGUCGGGUCAAAUACGACCAUAUUUGUACAUAAGGCUUUUCAAGAAGUGAACGU
UGCAACAGAGUCUGAGAGAGUCGGAAAAAACAAGGUUAUUGGUCAACUGAUAAAUCUU
UUCAAUAGUCAAGCUCCUAGGAGCGAUAAUUGGUUCAUGAUAAAGUUUUGUCAGUUGA
UAGAAAUCUUAGAUCCAACAGCGGUAAUAAAUGUUAACAUCGUCAUCAACCAAACCUA
UGUGGAUUGGAGAUGGGAUGGUGACAUGGUGUUGUCACUUAUGAACAUU

>Guadeloupe_mosquito_viruses_strain_AP43_segment1_MT808038
UCGUGGAUUAUCCCUUCAAUCGAUGGCGCAAGGCUCCAUAUGUAUUUCAAAAGAAAUC
CGGUUUGCACGUAGAAGGAUACCGCCAACAUGGCUUUAUUCAACGGGUUCACCACUGC
AGGUAGUCGCCUUAAUGAUGUCAGCUAUAAUGAUGUCAUUUGUGGUGAACCCGUUGAA
UAAAGCCAUGUUGGCGGUAUCCUUCUACGUACAAACCGGAUUUCUUUUAAAAUACAUA
UGGAGCCUUGCGCCAUCGAUUGAAGGGAUAAUCCACGAGCGAUUGUCAGAGACGUAUU
CUAAAGUGCAUAUGGAAACUUUGGAAACGUUUUUAUUAGACUAUAAGGUCUAUUUAA
UGGCUAUAGCAUGUGGUUUUGUAAUCCUUCUUUCGAGUUAUAUAUAUGUUGAGGUAA
GAAAUUGGUGGGGAAAAGACCACCGUGUGUUUGUGGUGCCUAAUGGCCAAGCUGGAGC
UGGCACUUAUGUCAGUGAGAAAGCUAUGCCGGGAAGUUCUUUUGAGGUGGCUAAAGUU
CUGCCUACUUUCCAGGCUAUGGUAAUGGCAUCGUUAGAUGGGAUUACUUAUCACGUUA
UGGGCCAGUGUUUCUGGGUCGAUGAGGGAUUAGCGACAACUGCUAACCCCAUUGCCGA
UCUCGAAUAUUUGUGUAUCUACAAGGACGAAGAGAAACGCAUCGAAGUAGGACCAAAA
ACAUUCGAGAUCGGCAAUGGGGAUUACGCAGUAUGUCGAAAUCCAACACUGAUAACGC
AAAAAUUGGGUUUGAGCAAAGCAAAGUUUGCGAGGCUGGCGGUCGAAAAAGAUGCCGG
CUUGAGUGUCAACAUCACAGCCAUGGGAAAGAGAACGAUAGGAUUCCUAGAUAGACAU
CCACGUUUUUCCCAUGGCUGUGAUAGUGUAAGCACCAUUAAAGGAUUUAGUGGAGCGC
CUUACUAUUUCGGCAGGACCAUAUUUGGGAUGCACCUGGGGUCGGACUCUAAGAACCU
UGGUUAUGAUGGGGCAUUCUUGAAAACUGAACUCAAGCCCAGUCGGGUUAUAAGAAAC
CAGUUAAAUUUAAGAACUGAAGAUAGUGCAAGUUUUCUAGAGCAGCAGUUAGAGCGUU
AUGAAGAAGUAGAGUAUUCUAGAUCUCCGUAUAACCCUGAUGAAUAUAAGGUUAGGAU
UGGAGGGAUGUACCAUAUUGUUGAUGAGGAAGUGUUGAGUACCGUCUUAAAGAGAAG
AUCCGCUAAGCGAGCACAGGGGGAUUUACAAUAUUUGGCUGAACAAGCUGAAGUUGGA
CCCCUCAUUGAUCUCGAAGCUGUAACCACUAAGGGAGACCUUACUAGGUCUCAACAGA
GUGGACAAAAACAGCAUCAGGAGCUUUCUACUGAGACGGAUCCGAUAAUAGCUAUGAC
UAAAGGGCUAUUGUCAGAAUUUGGCAAAACCUCAGUGUAUGUUCCUCCAGAACCUAAG
AGUAAAGACCAAGGGACAUGCACUAGUUGUGAGUCUUGUGGCGCCUAUAUAUGUUCUG
UACAUUCCUCUGAUAUUCCUGAAACGCCUACUGCGGAUGCCAAGAAAGCGGAAGAUUU
ACCUUUAGCGCCUAGGAAUGCCAUGACGUUUAAUGAUUCGGAAAACCUGAUCAGGGCU
CCAGCUGUAGUUGUUGGGGCCCGUGGCAUGGAAUAUCAGAGGGAAAGUGUACAGAGCC
ACGACUUGCCAAGAUGCAAGAAAAUGGAUUGCAUGUGCCAGAAACCUUUGGAGACCUG
CCUUAUGGAAUCCCGUACAUUGACGCGUGCUCCGCCAAAAGAAGCUUCCGCACGCACUG

UCAGAAACAAAAACAGACGGAUGCGCCGCCAACAGCAGAAGAAAGAACUAGAGCAGUU
CAAGCAGCUCUACGGACCUAUUCCGCAUGGAGGUGCGACCUCACAUCCGCAGCAGAUCC
CCACAAAUGGUUUGAUAGGGAAUUCAAUAAAGCACUAGAGGAGAUAGAUAUGGAUAGC
ACUCCUGGCUACUGUAUUCUAUCGCUCACAGCCUCUACCAAUGCACAACUCAUUGACUU
AAUUUGUAUAUGCUGUGAUCCGGAUAGAGUUAGUAUGGUACGAGAGCAUGUCAAAUU
AAGGUUCAAUGAGUUGUUGGCAGGUAAGGCUGUGAGCGAUUGUAUUAAGGUUUUUGU
GAAGCAAGAGCCGCAUAAAAUGAAGAAAAUUCAGGAAGGUACUUAUCGCUUGAUAAGC
GCUGUUAGCUUAAUUGAUACCUUUAUUGACAGAAUUUUGUUCGCUUGGAUAGCCAGAG
CUCAGAUGGAUUCCGUCGGGGAGACGCCAUGCCUCAACGGACGGAAUCCACCGAGAGC
CGGAUGGCGUAAGAUAGUGAACAGGUUUGGCAACCAAGCUGUAAAUUGUUUAGAUAGA
UCAGCUUGGGACUGGACUGUACAAGGCUAUUUAGUUGAUCUUUGGAUAUUGUUUUUCG
AACUGAAACCAGUGAACCCUUCACUCUUGUGGCUUAAGAUGAUGAAACUACGUUUUAA
GCUACUGUUUGAAGAGGUUUGGUUUCAGUUCGAAGACGGGACAAGAGUAAAGCAGGCA
ACGCGAGGAGUAAUGAAGAGUGGUUGCUAUUUAACUAUUUUGUUAAAUAGUCUCAGCC
AAAGUCUCUUACAUUACGUAGUCAACAGUAGAUGUGGGAAGCCCAUGACUAUGAAACA
ACCAUACUCGAUAGGUGAUGACACGGUACAGGAAGCUAUGGAUUGGCUUCUAGACUAU
GUUAGACAACUCGAGAUAUUGGGAAUCACGGUCAAAGGAGCUAAAGUCCAGCAUUGGG
UUGAAUUUGCGGGGUUCUGUUUUGACGGGAAAACUUGUUAUCCGGCAAAACAGAAACC
CAGCAAAUUCAACCCAAUGCUGGACUUUAGCUCCUUUGACCGUGAUUCCCAAUAUCUC
GACGUCAAAGGAGCUAAAGUCCAGCAUUGGGUUGAAUUUGCGGGGUGCUGUUUUAGAC
AACUCGGGAUAUUGGGAAUUGCGGUCAAAGGAGCUAAGGUCCAGUCUUUG

>Guadeloupe_mosquito_viruses_strain_AP43_segment2_MT808039
ACGUCACGGCGCCGUUCGGCACCGAACUAAUGUGUAACGAGACCGCUAAGAAACGGCG
CAAUAGGCGCAAUAAGAAACGUGGAGGAGCCGACGGCCCGAGCGGGGUUUCUGCUGAU
GGACGUAUUACCGUUCAGAGAGCUGAGCUCCUGUUCGAGAUUAAGGUGGCCAAAGGUA
GCUCUUCCACGUCUUAUUAUGAAGCCUUGCAGCCGACCUCGACCCGAAUGACGUGGCU
AAAGUCUCUGAGUGGUAGUUUCUCUCAGAUAAUUUGGCAUUCCGCGAGACUAGAGUAU
CGCCCUGCUGUGGGGGCGAUGAAAGAUGGCUCUCUAGUCGUCGGAGUCGACUGGGACC
CUACGACGACCGUGCCUAGUAAAAGCAAGGUCCAAAGUUGUACUCCUAACUUUCAGGU
CCCGGUAUGGCAGAAGAAGGAGUUGACUCUCCCAGCGUCUCGGUUGCAGAGUCGUAAG
UAUUAUGCUCUCUCAGAUUCAGGGGAUAUACCCAUGAUAGACAAAGCCCCUUGUGAGG
UUUUGGCCUAUUUGGCUUGUUCCUCGAAUAAGGAUGAGGAACAGUACUUUGGAGACAU
CUGGAUCCACUAUAAGGUAACCCUGCAGGGGCCUAAAUAGGGAGACGAGACGCCCCCC
CCAUCACCUCCAGGACCUAGUGUGUUUGAUUACCACAUGGUUCGGGAGGUAACCCUAU
AUGAAGUGCGGGCCAUUGUAAGAACUAAAUCUGAACGUUAUCAUACCCCGCCAUCUGG
AUCUCAUAAGGGUUUUAAUAUCCAGAUGUCGAACAGGGAUAUGAUAACGUAUGCAGCG
GCGGCUGAGGUUUCAGUCAAUGGAACCGAUUUAACUAUUGAGGAUAAUUAUAGCGUUU
CUCACGAUGAUUCGAAAGUGGACAACGUGAGUGAAAUUCAAUGCGCUUCAAUACCCCC
GACAUGGAAUGGUGAACCUGUAGGAUUGGUGCACAUUAGUUUAUCUUUGAAAAGUCCU
UAUUUUCCGAAGAUAGCGAUGUUUGAUUUUAAGGUUCAUUUCGUGUCAAAUAGGAACA
UUUUUGUUCCUAAGGCUCUUAAAGAAGUGAACGUUGCUAUUUCUGAGAGCGUAGAGAA
CAAGGUUAUUGGUCAACUGAUAAAUCUUUUCAAUAGUCAAGCUCCUAGGAGCGAUAAU
UGGUUCAUGAUAAAGUUUUGUCAGUUGAUAGAAAUCUUAGAUCCAACAGCGGUAAUAA
AUGUUAACAUCGUCAUCAACCAAACCUAUGUGGAUUGGUUGAUGACGAUGUUGACAUG
GUGUUGGAUAUUACGACCAAUAAUCACGUUCUAUAACUCUGUUACGUCUCUGUUGGAC
GUUAGGGAG

>Guadeloupe_mosquito_viruses_strain_AP44_segment1_MT808040

GGCACUGACACUAAUGCCAGUGAGAAAGCUAUGCCGGGAAGUUCUUUUGAGGUAGCUA
AAGUCCUGCCUACUUUCCAGGCUAUGGUAAUGGCAUCGUUAGAUGGGAUUACUUAUCA
CGUUAUGGGCCAGUGUUUCUGGGUCGAUGAAGGCUUAGUGACAGCAGCCCAUAACGUG
AUUGAGGGUUUUGAAUAUUUGUGUAUCUACAAGGACGAAGAGAAACGCAUCGAAGUA
GGACCAAAAACAUUCGAGAUCGGCAAUGGGGAUUACGCAGUAUGUCGCAAUCCACCAU
UGCCGACUCGAAUCUUUGGUUUGCUCAAAGCCCAAUUUUUGCGUUAUGCAGUCGAACU
UGAGUUCAGUUUGAGUGUUAACAUCAUAGCCAUGGGAAAGAGAACGAUAGGAUUCCUA
GAUAGACAUCCACAAUUUGGGUAUGUUCGUUAUAGUGGAAGCACCAUUAAAGGAUUUA
GUGGAGCUCCUUACUAUUUUGGCAGGACCAUAUUUGGAAUGCAUCUGGGGUCGGACUC
UAAGAACCUUGGUUAUGAUGGAGCAUUCUUGAAAACUGAACUCAAGCCCAGUCGGGUU
AUAAGAAUGCUCCAGUCAAAACCAAGGUUCUUAGAGUCCGACGCCAGUUUUCUAGAGC
AGCAGUUAGAGCGUUCUUCAAAAAUACAGUCCUCCAGAUCCCCGUAUAUUCCCGAUGA
AUAUAAGGUUAGGAUUGGAGGGAUGUAUCAUAUUGUUGAUGAGGAAGUGUUGAGUAC
CGUCUUAAAGAGAAGAUCCGCUAAGCGAGCACAGGGGGAUUUACAAUUUUUGACGGUA
CAAGCUGAAGUUGGGCCCCUCAUUGAUCUCGAAGCUGUAACCACUAAGGGCGACCUUA
CUAGGUCUCAACAGAGUGGACAAAAACAGCACCAGGAGCUUUCUACUGAGACGGAUCC
GAUAAUCUCCAUGACUUACUGUCUAUUGUCAGAAUUUUGCAAAACCUCAGUGUACGUU
CCUCCAGAACCUAAGAGUAAAGACCAAGGGACAUGCACUAGUUGUGAGUCUUGUGGCG
CCUAUAUAUAUUGGGGGGAGUCUAGGGAUAUUCCUGAAACGCCUACUGCGGAUGCCAA
GAAAGCGGAAGAUUUACCUUUAGCGCCUAGGAAUGCCAUGACGUUUAAUGAUUCGGAA
AACCUGAUCAGGGCUCCAGCUGUAGUUGUUGGGGCCCGUGGCAUGGAAUAUCAGAGGC
AAAGUGUACAGAACCACUGGCGCUGGCGACGAUGCAAGAAAAUGGAUUGCAUGUGCCA
GAAACCUUUGGAGACCUGCCUUAUGGAAUCCCGUACAUUGACGCGUGCUCCGCCAAAA
GAAGCUUCCGCACGCACUGUCAGAAACAAAAACAGACGGAUGCGCCGCCAACAGCAGA
AGAAAGAACUAGAGCAGUUCAAGCAGCUCUACGGACCUAUUCCGCAUGGAGGUGUGAC
CUCACAUCCGCAGCAGAUCCCCACAAAUGGUUUGAUAGGGAAUUCAAUAAAGCACUAG
AGGAGAUAGAUAUGGAUAGCACUCCUGGCUACUGUAUUUUGACAAAUAAAGUAGCCAG
GAAGACGCAAAUCUUUGGUUCGAAUGGUAUAUGCUGUGAUCCGGAUAGAGUUAGUAUG
GUACGAGAGCAUGUCAAAUUAAGGUUCAAUGAGUUGUUGGCAGGUAAGGCUGUGAGCG
AUUGUAUUAAGGUUUUUGUGAAGCAAGAGCCGCAUAAAAUGAAGAAAAUUCAGGAAG
GUACUUAUCGCUUGAUAAGCGCUGUUAGCUUAAUUGAUACCUUUAUUGACAGAAUUUU
GUUCGCUUGGAUAGCCAGAGCUCAGAUGGAUUCCGUCGGGGAGACGCCAUGUCUCAUC
GGGUGGAGUCCAGUUAGAGGAGGAUGGCGUAAGACAGUGAACAGGUUUGGCAAACAAG
CAGUAAAUUGUUUAGAUCGAUCGGCUUGGGAUUGGCCUGUACAAGGCUAUUUAAUUGA
UCUUAAGACAUUGAUUUUGGAAAAUUUACCAGUGAAUCCUCCAGACUGGUGGCUUAAG
AUGAUGAAACUACGUUUUAAGCUACUGUUUGAAGAGGUUUGGUUUCAGUUCGAAGACG
GGACAAGAGUAAAGCAGGCAACGCGAGGAGUAAUGAAGAGUGGUUGCUAUUUAACAAA
UUUGUUAAAUAGUCUUAACCAAACUCUCUUACAUCGCGUUGCCUGCUUUAGAUGUGGG
AAGCCCAUGACCAUGAAACAACCAUACUCGAUAGGUGAUGACACGGUACAGGAAGCUA
UGGAUUGGCUUCUAGACUAUGUUAGGCAACUCGAGAUAUUGGGAAUCACGGUCAAAGG
AGCUAAAGUCCAGCAUUGGGUUGAAUUCGCGGGGUUCUGUUUUGACGGAAAACUUGU
UAUCCGGCAUACUGGCAAAAACAUCUUUUUAACCUUAAGUAUACGCAAAAACGAGGCC
UAACUACGAGUACUUAUCAAAAUUCGUACGUCAAUGAGCCUGUGAUGUACGAAUUUGU
GUGUAGAGUGGCUCGUGAGUUAGGUCCUCGUUUCGUCUUGCCUAAGAUAGAAGCACUU
GACAUAAGGAAUCAAUUUGGUUGAUUCAUUAUGUCAAGUGCUUCUAU

>Guadeloupe_mosquito_viruses_strain_AP44_segment2_MT808041
UGGCAUUCCGCAGAUAGAGUAUCGCCCUGCUGGGGGGCGAGCUCUAGUUCGCGAUGAA
GGUGGCCAAAGGUAGUUCUUCCACGUCUUAUUAUGAAGCCUUGCAGCCGACCUCGACC

CGAAUGACGUGGCUAAAGACUCUGAGUGGUAGUUUCUCUCAGAUAAUUUGGCAUUCCG
CGAGACUAGAGUAUCGCCCUGCUGUGGGGGCGAUGAAAGAUGGCUCUCUAGUCGUCGG
AGUCGACUGGGACCCUACGACGACCGUGCCUAGUAAAAGCAAGGUCCAAAGUUGUACU
CCUAACUUUGAGGUCCCGGUAUGGCAUAUGAAGGAGUUGACUCUCCCAGCGUCUCGGU
UGCAGAGUCGUAAGUAUUAUGCUCUCUCAGAUUCAGGGGAUAUACCCAUGAUAGACAA
AGCCCCUUGUGAGGUUUUGGCCUAUUUGGCUUGUUCCUCGAAUAAGGAUGAGGAACAG
UACUUUGGAGACAUCUGGAUCCACUAUAAGGUAACCCUGCAGGGGCCUAAAUAGGGAG
ACCACCAGCCACCCCCAUCGCCUCCAGGACCUAGUGUUUUCGACUACUACAUGGUUACG
GAUGUAGCCCUACGCGAAGUGCGGGACAUCGUAAGAACUAUAGAUGAGCCCCAACAUA
AACAGACUUCUGGAUAUUAUAAACCUUUUGAGAUCCAGAUGGCGAACAGGGAUAUGAU
AACGUAUGCAGCGGCGGCUGAGGUUUUAGUCAAUGGAACCGAUUUAACUAUUGAGGAU
UAUUAUAGCUUUUCUCACGAUGAUUCGAAAGUGGAGAAUGUGAGUGCAAGUCAAGGCG
CUUCAAGGCCCCCGACAUGGAAUGUUGAACCUGUAGGAUUGGUGCACAUUGGUUUAUC
UUUGAAAAGUCCUUAUUUUCCGAAGAAAGCGAUGUUUGAUUAUACGGUUCAUUUCGUG
AAAAAUAGGAACAUCCUUCUUCGUACGGCUUUUAACGAAAUGAACGUUGCUAUUUCUG
AGAGCGCGAAAAUCAAGGUUAUUGGUCCUGUAAUGAAUCUUUUCAAUAGUCAAGCUCC
UAGGAGCGAUAAUUGGUUCAUGAUAAAGUUUUGUCAGUUGAUACCAAUUAUCGCUCCA
GGAGCGGUAAUAAAUGUUAACAUCGUCAUCAACCAAAGCCAUGUGGAUUGGAGAUGGG
AUGGUGACGUGGUGUUGGAUAUUACGCCAAUAAUCACG

>Guadeloupe_mosquito_viruses_strain_AP47_segment1_MT808042
CCCCCUUCCCCCCCCACUUCCCCCCCUCACCAUAUCUAUAGCUCGAAAGAAGGAUAUCA
AAACCACAUGCUAUCCUUCUUUCGAGCUAUAUAUAUGUUGAGGUUAGAAAUUGGUGGG
CAAAAGACCACCGUGUGUUUGUGGUGCCUAAUGGCCAAGCUGGAGCUGGCACUUAUGU
CAGUGAGAAAGCUAUGCCGGGAAGUUCUUUUGAGGUGGCUAAAGUCCUGCCUACUUUC
CAGGCUAUGGUCAUGGCAUCGUUACAUGGGAUUAUCCAACCCCUCAUGGGCUGCUGUC
ACUAAGCCUUCAUCGACUCCGAAACAAUGGCCCACGUUAUUGAGGGUUUUGAAUAUUU
GUGUAUCUACAAGGACGAAGAGAAACGCAUCGAAGUACGACCAAAAACAUUCGAGAUC
GGCAAUGGGGAUUACGCAGUAUGUCGCAAUCCAACACUGCUCACGCAAAAAUUGGGUU
UGGGCAAAGCAAAGUUUGCGAGGCUGGCGGUCGAAAAAGAUGCCGGCUUUAGUGUUAA
CAUCACAGCCAUGGGAAAGAGAACGAUUGGAUUCCUGGAUAGACAUCCACAAUUUGGG
UAUGUUCGUUAUAGUGGAAGCACCAUUAAAGGAUUUAGUGGAGCUCCUUACUAUUUUG
GCAGGACCAUAUUUGGAUUSCAUCUGGGGUCGGAAUCUAGAGAUCUUCAUAAUGAUGG
UGCAUUCUUUAAAACUGAACUCAAGCCCAGUCGGGUUAUACGAAACCAGUUUAAUUUU
AGAACUGAAGAUAGUGCAAGUUUUCUUAAGCAGCAGUCUGAACGUUAUGAAGAAGUGG
AGUAUUCUAGAUCUCCGUAUAAUCCCGAUGAAUAUAAGGUUAGGAUUGGAGGGAUGUA
UCAUAUUGUUGAUGAGGAAGUGUUUAGUACCGUCUUUAAGAGAAGAUCCCGCUGUGCG
CGCACAGGCGGAUCUUCUCUUUAAGACGGUACUCAACGCUGAAGUUGGGCCCCUCAUU
GAUCUCGAAGCUGUAACCACUAAGGGAGACCUUACUAGGUCUCAACAGAGUGGACAAA
AACAGCACCAGGAGCUUUCUACUGAGACGGAUCCGAUAAUAGCUAUGACUAAAGGGCU
AUUGUCAGAAUUUGGCAAAACCUCAGUGUACGUUCCUCCAGAACCUAAGAGUAAAGAC
CAAGGGACAUGCACUAGUUGUGAGUCUUGUGGCGCCUAUAUAUAUYRGKRGGMGUYUC
AGGAAUAUCAGAGGGAAAGUGUACAGAGGAUGCCAAGAAAGCGGAAGAUUUACCUUUG
GCGCCUAGGAAUGCCAUGUCGUUUAAUGAUUCGGAAAACCUGCUCAUGGCUCCAGCUG
UAUUUGUUGGGGCCCGUGGCAUGGAAUAUCAGAGGGAAAGUGUACAGAGCCACGACUU
GCCAAGAUGCAAGAUACUGGAUUGCAUGUGCCAGAAACCUUUGGAGACCUGCCUUAUG
GAAUCCCGUACAUCGACGCGUGCUCCGCCAAAAGAAGCUUCCGCACGCACUGUCAGAAA
CAAAAACAGACGGAUGCGCCGCCAACAGCAGAAGAAAGAACUACAAUAGGUCGAACUG
CUCUAGUUCUUUCUUCUGCAUGUAUGUGCGGUCUCACAUCCGUAGCAGUUCCCCAUCA

AAUGGUUCGAUUGGGAAUUCAACAAAGCACUAGAGGAGAUAGAUAUGGAAAGCACUCC
UGGCUACUGUAUUUUGACUCUUUUAGGCUCUACGAAUGCGCAAAUCUUCGGUUGGAAU
GGUAUAUGCUGCGAUCCGGACAGAGUGAGUAUGGUACGAGAGCAUGUCAAAUUAAGGU
UCAAUCAGUUGUUGGCAGGAAAGGCUGUGAGCGAUUGUAUCAAGGUUUUUGUGAAGCA
AGAGCCGCAUCAAAUGAAGAAAAUUCAGGAAGGUACUUAUCGCUUGAUAAGCACCGUA
UGCUUAAUCGAUUCCUUUAUAUGCAGAGUCUUGCUUCACAAAAUAGCCAGAGCUCAGA
CGGAUCCCGUCAGCUAGACGCCAUGUCUCAUCAGGUGGAGUCCAGUUCGAGGCGGAUG
GCGUACGAUAGUGAACAGGUUUGGCAACCAAGCAGUAAAUUGUUUAGAUCGAUCGGCU
UGGGAUUGGACUGUACAAGGCUAUUUAGUCGAUCUUUGGAUAUUCCAAUCCCAAGAAA
ACUUACCACAAAACCUCUCAGACUGGUGGCUAGAGAUGAUGAAACUACGUUUCAAGCU
ACUGUUUCAAGAGGUUUGGUUUCAGUUCGAAGACGGAACAAGAGUAAAGCAGGCAACG
CGAGGAGUAAUGAAGAGUGGUUGCUAUUUAACUAUUUUGUUAAAUCGCAACCACUCUU
CAUUACAUUACGUAGUCAACAGUCGAUGUGGGAAGCCCAUGACCAUGAAACAACCAUA
CUCGAUAGGUCACGACACGGUACAGGAAGCUAUGGAUUGGCUUCUAGACUAUGUCAGA
CAACUCGAGAUAUUGGGAAUCACGGUCAAAGGAGCCAAAGUCCAGCAUUGGGUUCAAU
UUGCGGGGUUCUGUUUCGACGGGAAAACUUGUUAUCCGGCAUACUGGCAAAAACAUCU
UCUCAAACUGAUAACUACGCAAAAUCUUGGGGAGACUUUGCGGAGUUAUCAGUAUUUG
UACGUCAAUCAGCCUGUGAUGUACGAAUUUGUGUGCAGAGUGGCUCGGGAGUUAGGUC
CUCGUUUCGUCUUGCCCAAGAUAGAAGCACUAGACAUAAUGGAUCAAUUUGGUUGAUU
CAUUAUGUCUAGUGCUUCUAUCUUAGGCAAGACGAAACGAGGACCUAACUCACGAGCC
ACUCUACACACAAAUUCGUACAUCACAGGCUCAUUGACGUACGAAUUUGUGUGUAGAG
UGGCUCGUGAGUUAGGUCCUCGUUUCGUCUUGCCUAAGAUAGAAGCACUAGACAUAAU

>Guadeloupe_mosquito_viruses_strain_AP47_segment2_MT808043
UGUUAAAAUAAACAAUGAGUAAACAGUUGAAGAAACAGUUUCUUGCAUCUUUAACUGC
UCAAGUAGCUGCGAUGAGGGUUGCCAAACCGCUAACCUUCAACGCUUCUGUUAAGAAA
CGGCGCAAUAGGCGCAAUAAGAAACGUGGAGGAGCCACUGGCCCGAGCGGGGUUUCUG
CUGAUGGACGUAUUACCGUUCAGAGAGCUGAGCUCCUGUUCGAGAUUAAGGUGGCCAA
AGGUAGCUCUUCCACGUCUUAUUAUGAAGCCUUGCAGCCGACCUCGACCCGAAUGACG
UGGCUAAAGACUCUGAGUGGUAGUUUCUCUCAGAUAAUUUGGCAUUCCGCGAGACUAG
AGUAUCGCCCUGCUGUGGGGGCGAUGAAAGAUGGCUCUCUAGUCGUCGGAGUCGACUG
GGACCCUACGACGACCGUGCCUAGUAAAAGCAAGGUCCAAAGUUGUACUCCUAACUUU
CAGGUCCCGGUAUGGCAGAAGAAGGAGUUGACUCUCCCAGCGUCUCGGUUGCAGAGUC
GUAAGUAUUAUGCUCUCUCAGAUUCAGGGGAUAUACCCAUGAUAGACAAAGCCCCUUG
UGAGGUUUUGUCCUAUUUGGCUUGUUCCUCGAAUAAGGAUGAGGAACAGUACUUUGGA
GACAUCUGGAUCCACUAUAAGGUAACCCUGCAGGGGCCUAAAUAGGGAGACGAGACGC
CCCCCCCAUUACCUCCAGGACCUAGUGUGUUUGAUUACCACAUGGUUCGGGAGGUAAC
CCUAUAUGAAGUGCGGGCCAUUGUAAGAACUGCAUAUUAUCGAUAUCCUGUUCCGCCA
UCUGGAUCUCAUAAGGGUUUUAAUAUCCAGAUGUUGAACUGGGAUAUGAUAACGUAUG
UAGCGGCGGCUGAGGUUUCAGUCAAUGGAACCGAUUUAACUAUUGAGGAUAAUUAUAG
CGUUUCUCACGAUGAUUCGAAAGUGGACAACGUGAGUGAAAUUCAAUGCGCUUCAAUA
CACCCGACAUGGAAUGUUGAACCUGUAGGAUUGGUGCACAUUGGUUUAUCUUUGAAAA
GUCCGUAUUUUCCGAAGAUAGCGAUGUUUGAUUUUACGGUUCAUUUCGUGUCAAAUAG
GAACAUUUUUGUUCCUAAGGCUCUCAAAGAAGUGAACGUUGCCAUUUCAGAGAGCGGG
ACCGUCAACAUUACUGGUCAACUGAUAAACUUUAUCAAUAGUCAAGCUCCUAGGAGCG
AUAAUUGGUUCAUGAUAAAGUUUUGUCAGUUGAUAGAAAUCUUAGAUCCAACAGCGGU
AAUAAAUGUUAACAUCGUCAUCAACCAAACCUAUGUGGAUUGUAGAUGGGAUGGUGAC
AUGGUGUUGGAUAUUACACCAAUAAUCACGUUCUAUAACUCUGUUACGUCUCUGUUGC
ACUUCA

>Guadeloupe_mosquito_viruses_strain_AP49_segment1_MT808044
UUCCCACAUCCCCUUCAGACACGUCUCUAUUAGACCACAAUCGCUCGUGGAUUAUCCCU
UCAAUCGAUGGCGCAAGGCUACCAUACUGUUAUAUGUAUGUUGGCACGAACAUUCGGU
GGGCAAAAGACCACCGUGUGUUUGUGGUGCCUAUUGGCCAAGCUGGAGCUGGCACUUA
UGUCAGGGAGAAAGCUAUGCCGGGAAGUUCUUUCGAGGAAGCCAAAGUCCUGCCUACU
UUCCAGGCUAUGGUCAUGGCAUCGUUACAUGGGAUUACCUAUCCUGGUAUGGGGCAGU
AUCUUCAAGUCAAUCAGGGCUUAGUGACAGCAGCCCACGUGAUGGAGGGUUUCGAAUA
UUUGUGUAUCUACAAGGACGAAGAGAAACGCAUCGAAGUAGGACCAAAAAUAUUCAAA
ACCCUCAAUCACGUUUGGGCAGUGUGUCUAAAGCCCACAGUGAUGGCUGUGAUGUUAA
CACUCAAGCCGGCAUCUUUUUCGAGGCUGGCUGUCGUAAAAGUUGCCGGCUUGAGUGU
CAACAUCACAGCCAUGGGAAAGAGAACGAUAGGAUUCCUAGAUCGACAUCCACAAUUU
GGGUAUGUUCGUUAUACUGGAAGCACCACCAGAUCCUUCAGUGGUGCUUCCACUAUAA
CGACAUACCCAAAUUGUGGAUGUGCUAUCUGGGGUCGGACUCUAAGAACCUUGGUUAU
GAUGGAGCAUUCUUGAAAACUGAACUCAAGCCCAGUCGGGUUAUAAGAACCUGGUUAA
AUGUAAGAUCUUAUGAUAGUGCAAGUUUUCUAGAGCAGCAGUUAGAGCGUUAUGAAG
AAGUAGAGUAUCUCAGAUCCCCGUAUAAUCCCGAUGAAUAUAAGGUUAGGAUUGGAGG
GAUGUAUCAUAUUGUUGAUGAGGAAGUGUUGAGUACCGUCUUAAAGAGAAGAUCCGCU
AAGCGAGCACAGGGGGAUUUACAGGUUCUGGAGGAACACACUGAAGUUGGGCCCCUCA
UUGAUCUCGAAGCUGUAACCACUAAGGGAGACCUUACUAGGUCUCAACAGAGUGGACA
AAAACAGCACCAGGAGCUUUCUACUGAGACGGAUCCGAUAAUAGCCAUGACUAAAGGG
CUAUUGUCAGAAUUUGGCAAAACCUCAGUGUACGUUCCUCCAGAACCUAAGAGUAAAG
ACCAAGGGACAUGCUCCAAUCCUGAACCUUAUAUUCAUCGGGAUUAUACAGGAGAUCU
CAGGGAUAUUCCUGAAACGCCUACUGCGGAUGCCAAGAAAGCGGAAGAUUUACCUUUA
GCGCCUAGGAAUGCCAUGACGUUUAAUGAUUCGGAAAACCUGAUCAGGGCUCCAGCUG
UAGUUGUUGGGGCCCGUGGCAUGGAAUAUCAGAGGGAAAGUGUACAGAGCCACGACUU
GCCAAGAUGCAAGAAAAUGGAUUGCAUGUGCCAGAAACCUUUGGACACCUGCCUUCUG
GAAUCCCGUACAUUGACGCGUGCUCCGCUAAAAGAAGCUUCCGCACGCACUGUCAGAA
ACAAAAACAGACGGAUGCGCCGCCAACAGCAGAAGAAAGAACUAGAGCAGUUCAAGCA
GCUCUACGGACCUAUUCCGCAUGGAGGUGUGACCUCACAUCCGCAGCAGAUCCCCACAA
AUGGUUUGAUAGGGAAUUCAAUAAAGCACUAGAGGAGAUAGAUACAGAUAGCACAGG
AGGCUAUCCAUAUCUAUCUCUUCUAGGCUCUACGAAUGCCCUAAUCAUUUGUUGGAAU
GGUAUAUGCUGUGAUCCGGAUAGAGUUAGUAUGGUACGAGAGCAUGUCAAAUUAAGG
UUCAAUGAGUUGUUGGCAGGUAAGGCUGUGAGCGAUUGUAUUAAGGUUUUUGUGAAG
CAAGAGCCGCAUAAAAUCGAGAAAAUUCAGGAAGGUACUUAUCGCUUGAUAAGCACCU
UCCUGAAUGUUAUGUCAUUUCCUGACGGAAUUUUCUUCGCUUGGAUAGCCAGAGCUCU
AAUGGAUUCCGUCGGGGAGACGCCAUGUCUCAUCGGGUGGAGUCCAGUUAGAGGCGGA
UGGCGUAAGAUAGUGAACAGGUUUAGGUCCAACCAAGCAGUAAAUUGUUUAGAUAGAU
CGGCUUGGGAUUGGACUGUACAAGGCUAUUUAGUUGAUCUUUGGAUAUUGUUUUUGG
AAAAUUUACCAGUGAAUCCUCCAGACUGGUGGCUUAAAACGUUGUUUCAUCAUCUUAA
GCCACCGUCUGGAGGAUUCUGGUAUCAGUUCGAAGACGGAUCAUGGGCUUCCCACAUC
UACUGUGGAGUAAUGAAGAGUGGCUACUAUUUAACAAAAUUGUUAAAUAGUCUUAGCC
AAAGUCUCUUACAUUACGUAGUCAACAGUAGAUGUGGGAAGCCCAUGACCAUGAAACA
ACCAUACUCGAUAGGUGAUGACACGGUACAGGAAGCUAUGGAUUGGUUUCUGGACUGU
GUUACACAUCUCGAGAUAUUGGGAAUCACGGUCAAAGGAGCUAAAGUCCAGCAUUGGG
UUGAAUUUGCGGGGUUCUGUUUUGACGGGAAAACUUGUUAUCCGGCAUACUGGCAAAA
ACAUCUUUUUAACCUUAAGUAUACGCAAAAUCUUGGGGAGACUCACGAGCUACACACA
AAUUCGUACAUCACAGGCUCAUUGACGUACGAAUUUGUGUGUAGAGUGGCUCGUGAGU
UAGGUCCUCGUUUCGUCUUGCCUAAGAUAGAAGCACUAGACAUAAUGAAUCAACCAAA

UUGAUCCUAUGUCUAGUGCUUCUAUCUUAGGCAAGACGAAACGAGGACCUAACUCACG
AGCCACUCUACA

>Guadeloupe_mosquito_viruses_strain_AP49_segment2_MT808045
AUGAAGGCUGCCAAACCGCAACCUAACGUUUCUUAGCAGAAGCGUUAGGUUGCGGUUU
GGCAACCCUCAUCGCAGCUACUUGAGCAGCUUAAAGACACAACUUGCAUCUUUAACAG
CUUAAGCAGUUGUGUUCAACGUUACGAUUUAGUCAAGCUACAUGAGCUUCUUAGGUUA
AAGAUGCAAGCUGUAUCAUCAACUGAUUACUCAUUGCAUUAUUUAACACAAUAAGUA
AACAGUUGAAGAAACAGCUAGCAUCUUUAACUGUUUACUCAUUGUGUUAGAAUAAAGA
AAUUAGUAAGUGAUUUUAUGCUUCCUUGGUUAGCCCUUAUAUAACUGAGUUUAAUUGC
UUUAUUUUAACACAAUGAGUAAACAGUUGAAGAUCCAUCUUGCAUCGCCCCCAACAGG
GCAACUAUCUUCGCGGAAGGUUGCCAAACCGCAACCUAACGCUUCUGCUAAGAAACGG
CGCAAUAGGCGCAAUAAGAAACGUGGAGGAGCCGCUGGCCCGAGCGGGGUUUCUGCUG
AUGGACGUAUUACCGUUCAGAGAGCUGAGCUCCUGUUCGAGAUUAAGGUGGCCAAAGG
UAGUUCUUCCACGUCUUAUUAUGAAGCCUUGCAGCCGACCUCGACCCGAAUGACGUGG
CUAAAGACUCUGAGUGGUAGUUUCUCUCAGAUAAUUUGGCAUUCCGCGAGACUAGAGU
AUCGCGGAAUGCAAAUUAUGGGGGCGAUGAAAGAUGGCUCUCCAGAGGGUCGUACCUC
UAGUUGGGACCCCAGGGCGACAGUGCAGGGUGAAGGUAAUGUCCUUAGUUGUACUCCU
AACCUUGAGGUCCCGGAAUCGCAGAAGAUGGAGUUGACUCUCCCAGCGUCUCGGUUGC
AGAGUCGUAAGUAUUAUGCUCUCUCAGAUUCAGGGGAUAUACCCAUGAUAGACAAAGC
CCCUUGUGAGGUUUUGGCCUAUUUGGCUUGUUCCUCGAAUAAGGAUGAGGAACAGUAC
UUUGGAGACAUCUGGAUCCACUAUAAGGUAACCCUGCAGGGGCCUAAAUAGGGAGACG
AGACGCCCCCCCCAUUACCUCCAGGACCUAGUGUGUUUGAUUACCACAUGGUUCGGGA
GGUAACCCUAUAUGAAGUGCGGGCCAUUGUAAGAACUAAAUAUGAGUCACACCCUGUU
CAGCCAUCUGGAUAUUAUAAACCCUUUGAGAUCCAGAUGGCGAACAGGGAUAUGAUAA
CGUAUGCAGCGGCGGCUGAGGUUUCAGUCAAUGGAACCGAUUUAACUAUUGAGGAUAA
UUAUAGCGUUUCUCACGAUGAUUCGAAAGUGGACAACGUGAGUGAAAUUCAAUGCGCU
UCAAGACCCCCGACAUGGAAUGUUGAACCUGUAGGAUUGGUGCACAUUGGUUUAUCUU
UGAAAAGUCCGUAUUUUCCGAAGAUAGCGAUGUUUGAUUUUACGGUUCAUUUCGUGUC
AAAUAGGAACAUUUUUGUUCCUAAGGCUCUUAAAGAAGUGAACGUUGCUAUUUCUGAG
AGCGCGAAGAUCAAGGUUAUUGGUCCUGUAAUGAAUCUUUUGAACAAUUACGCUCCUA
GGAGCGAUAAUUGGUUCAUGAUAAAGUUUUGUCAGUUGAUAGAAAUCUUAGAUCCAAC
AGCGGUAAUAAAUGUUAACAUCGUCAUCAACCAAACCUAUGUGGAUUGGAGAUGGGAU
GGUGACAUGGUGUUGGAUAUUACGCCAAUAAUCACGUUCUAUAACUCUGUUACGUCUC
UGUUGCACUUUAGGGAGGGGGAGGGUGAGAGUUG

>Guadeloupe_mosquito_viruses_strain_AP50_segment1_MT808046
UGUCAUCGCUUCAUGCGAUCCCUAAUCACGUUAUGGGCCAGUGUUUCUGGGUCGAUGA
AGGCUUAGUGACAGCAGCCCACGUGAUUGAGGGUUUUGAAUGUUUUUGUAUCUACAAG
GACGAAGAGAAACGCAUCGAAGUAGGACCAAAAACAUUCGAGAUCGGCAAUGGGGAUU
ACGCAGUACGGCGCAAUCCAACACUGAUAACGCAAAAAUUGGCUUUGAGCAAAGCAAA
GUUUGCGAGGCUGGCGGUCGAAAAAGAUGCCGGCUUGAGUGUUAACAUCACAGCCAUG
GGAAAGAGAACGAUAGGAUUCCUAGAUAGACAUCCACAAUUUGGGUAUGUUCGUUAUA
GUGGAAGCACCAUUAAAGGAUUUAGUGGAGCUCCUUACUAUAUUGGCAGGACCAUAUU
UGGAAUGCAUCUGGGGUCGGACUCUAAGAACCUUGGUUAUGAUGGGGCAUUCUUGAAA
ACUGAACUCAAGCCCAGUCGGGUUAUAAGAAACCAGUUAAAUUUAAGAACUGAAGAUA
GUGCGAGUUUUCUAGAGCAGCAGUUAGAGCGUUAUGAAGAAGUAGAGUAUUCUAGAUC
UCCGUAUAAUCCCGAUGAAUAUAAGGUUAGGAUUGGAGGGAUGUAUCAUAUUGUUGA
UGAGGAAGUGUUGAGUACCGUCUUAAAGAGAAGAUCCGCUAAGCGAGUACAGGGGAU

UUACAAUAUUUGGCUGAACAAGCUGAAGUUGGGCCCCUCAUUGAUCUCGAAGCUGUAA
CCACUAAGGGAGACCUUACUAGGUCUCAACAGAGUGGACAAAAACAGCACCAGGAGCU
UUCUACUGAGACGGAUCCGAUAAUAGCUAUGACUAAAGGGCUAUUGUCAGAAUUUGGC
AAAACCUCGGUGUACGUUCCUCCAGAACCUAAGAGUAAAGACCAAGGGACAUGCACUA
GUUGUGAGUCUUGUGGCGCCUAUAUAUAUUGGGGGGAGUCUAGGGAUAUUCCUGAAAC
GCCUACUGCGGAUGCCAAGAAAGCAGAAGAUUUACCUUUAGCGCCGAGGAAUGCUAUG
ACGUUUAAUGAUUCGGAAAACCUGAUCAGGGCUCCAGCUGUAGUUGUUGGGGCCCGUG
GCAUGGAAUAUCAGAGGGAAAGUGUACAGAGCCACGACUUGCCAAGAUGCAAGAAAAU
GGAUUGCAUGUGCCAGAAACCUUUGGAGACCUGCCUUAUGGAAUCCCGUACAUUGACG
CGUGCUCCGCUAAAAGAAGCUUCCGCACGCACUGUCAGAAACAAAAACAGACGGAUGC
GCCGCCAACAGCAGAAGAAAGAACUAGAGCAGUUCAAGCAGCUCUACGGACCUAUUCC
GCAUGGAGGUGUGACCUCACAUCCGCAGCAGAUCCCCACAAAUGGUUUGAUAGGGAAU
UCAAUAAAGCACUAGACCAGAUAGCUAUCGAUAUCACUCCUGGCUACUGUUUAUUGAC
UCUUUUAGGCUCUACGAAUGCGCAAAUCUUUGGUUGGAAUGGUAUAUGCUGUGAUCCG
GAUAGAGUUAGUAUGGUACGAGAGCAUGUCAAAUUAAGGUUCAAUGAGUUGUUGGCA
GGUAAGGCUGUGAGCGAUUGUAUUAAGGCUCUUGUUCAGCAAAAACCUAAUACAAUGC
UCACAGUUCAGUCCAAUACCAACCGAUCUAUAAACACUUUACGCUUAAUUGAUACCUU
UAUUGACAGAAUUUUGUUCGCUUGGAUAGCCAGAGCUCAGAUGGAUUCCGUCGGGGAG
ACGCCAUGUCUCAUCGGGUGGAGUCCAGUUAGAGGCGGAUGGCGUAAGAUAGUGAACA
GGUUUGGCAACCAAGCAGUAAAUUGUUUAGAUAGAUCGGCUUGGGAUUGGACUGUACA
AGGCUAUUUAGUUGAUCCUUGCAUAUUGUAUUUAGAAAAUUUACCAGUGAAUCCUCCA
GACUGGUGGCUUAAGAUGAUGAAAUUACGUUUUAAGCUACUGUUUGAAGAGGUUUGG
UUUCAGUUCGAAGACGGGACAAGAGUAAAACAGUCUACGCGACUAGUAAUGAAGAGUG
GCUGCUAUUUAACAAUUUUGUUAAAUAGUCUCAGCCAAAGUCUCUUACAUUACGUAGU
CAACAGUAGAUGUGGGAAGCCCAUGACUAUGAAACAACCAUACUCGAUAGGUGAUGAC
ACGGUACAGGAAGCUAUGGAUUGGCUUCUAGACUAUGUUAGACAACUCGAGAUAUUGG
GAAUCACGGUCAAAGGAGCUAAAGUCCAGAAUUGGGUUGAAUUUGGGGGGUCUGUUU
UGACGGGAAAACUUGUUAUCCGGCAUACUGGCAAAAACAUCUUUUUAACCUUAAGUAU
ACGCAAACACUAGGACAAACUCACGAGCCUCUACACACAAAUUCGUACGUCAAUGAGC
CUGUGAUGUACGAAUUUGUGUGUAGAGUGGCUCGUGAGUUAGGUCCUCGUUUCGUCUU
GCCUAAGAUAGAAGCACUAGACAUAAUGAAUCAACCAAAUUGAUCCUAUGUCAAGUGC
UUCUAUCUUAUGGCGAAUGAAACGAAACGAGUCGACCUAACUCCGAGCCACUCUACAC
ACAAAUCUACACACAAAUUCGUACGUCAAUGAGCCUGUGAUGUACGAAAUUUGUGUGU
AGAGUGGCGCGGAGGUAGGUCCUCGUUUCGGUUUGCGUAGUUAUCAGUAUUUGUA

>Guadeloupe_mosquito_viruses_strain_AP50_segment2_MT808047
CUUUGGUACCCAAAGGUGCUUUUACUACGCACGGUCGUGUAGGGUCCCAGCCGACCUC
GACCCGAAUGACGUGGCUAAAUACUCUGAGGGGAAGUUUCUCUCAGAUAAUUUGGCAU
UCCGCGAGACUAGAGUAUCGCCCUGCUGUGGGGGCGAUGAAAGAUGGCUCUCUAGUCG
UCGGAGUCGACUGGGACCCUACGACGACCGUGCCUAGUAAAAGCAAGGUCCAAAGUUG
UACUCCUAACUUUCAGGUCCCGGUAUGGCAGAAGAAGGAGUUGACUCUCCCAGCGUCU
CGGUUGCAGAGUCGUAAGUAUUAUGCUCUCUCAGAUUCAGGGGAUAUACCCAUGAUAG
ACAAAGCCCCUUGUGAGGUUUUGGCCUAUUUGGCUUGUUCCUCGAAUAAGGAUGAGGA
ACAGUACUUUGGAGACAUCUGGAUCCACUAUAAGGUAACCCUGCAGGGGCCUAAAUAG
GGAGACGAGACGCCCCCCCCAUUACCUCCAGGACCUAGUGUGUUUGAUUACCACAUGG
UUCGGGAGGUAACCCUAUAUGAAGUGCGGGCCAUUGUAAGAACUAAAUAUGAGCCCCA
ACAUAAACAGACUUCUGGAUAUUAUAAACCUUUUGAGAUCCAGAUGGCGAACAGGGAU
AUGAUAACGUAUGCAGCGGCGGCUGAGGUUUUAGUCAAUGGAACCGAUUUAACUAUUG
AGGAUAAUUAUAGCGUUUCUCACGAUGAUUCGAAAGUGGACAACGUGAGUGAAAUUCA

AUGCGCUUCGAGACCCCCGACAUGGAAUGUUGAACCUGUAGGAUUGGUGCACAUUGGU
UUAUCUUUGAAAAGUCCGUAUUUUCCGAWSWUCGCGCUCUCAGAAAUAGCAAUUCACU
UCGUUUCAAGCCUUAGGAACAAAAAUGUUCCUAAGGCUCAUAAAGAAGUGAACGUUGC
UAUUUCUGAGAGCGCGAAGAUCAAGGUUAUUGGUCCUGUAAUGAAUCUUUUCAAUAGU
CAAGCUCCUAGGAGCGAUAAUUGGUUCAUGAUAAAGUUUUGUCAGUUGAUAGAAAUCU
UAGAUCCAACAGCGGUAAUGAAUGAUGACAUGUUAACAUUAUUAC

>Guadeloupe_mosquito_viruses_strain_AP53_segment2_MT808048
CUUACUAAAUUCUUUAUUUUAACGCAAUGAGUAAACAGUUGAAGAAACAGCUUGCAUC
UUUAACUGCUCAAGUAGCGGCGAUGAAGGUUGCCAAGCCGCAACCUAACGCUUCUGCU
AGCACCCGUCGCGCUAGUCCCAAUAAGAAGCGUGGAGGAGCCGCUGGCCCGAGCGGGG
UUUCUGCUGAUGGACGUAUUACCGUUCAGAGAGCUGAGCUCCUGUUCGAGAUUAAGGU
GGCCAAAGGUAGUUCUUCCACGUCUUAUUAUGAAGCCUUGCAGCCGACCUUAAUCUCG
AACAGGUGGCUCAGCUCUCUGAACGGUCACUCCCCUCAGAUAAUUUGGCAUUCCGCGA
GACUAGAGUAUCGCCCUGCUGUGGGGGCGAUGAAAGAUGGCUCUCUAGUCGUCGGAGU
SGACUGGACUCCUACGACGACCGUGCCUAGUAAAAGCAAGGUCCAAAGUUGUACUCCU
AACUUUCAGGUCCCGGUAUGGCAGAAGAAGGAGUUGACUCUCCCAGCGUCUCGGUUGC
AGAGUCGUAAGUAUUAUGCUCUCUCAGAUUCAGGGGAUAUACCCAUGAUAGACAAAGC
CCCUUGUGAGGUUGUGGCCUAUUUGGCUUGUUCCUCGAAUAAGGAUGAGGAACAGUAC
UUUGGAGACAUCUGGAUCCAUGGUAAUCCAACCACACUAGGUCCUGGAUAGGGGGGCG
UCUCGUCUCCCCCAUUACCUCCAGGACCUAGUGUGUUUGAUUACCACAUGGUUCGGGA
GGUGACCCUAUAUGAAGUGCGGGCCAUUGUAAGAACUAAAUAUGAGCCCCAACAUAAA
CAGACUUCUGGAUAUUAUAAACCCUUUGAGAUCCAGAUGGCGAACAGGGAUAUGAUAA
CGUAUGCAGCGGCGGCUGACGUUUCAAUCAAUGGAACCAGUUUAACUAUUGAGGAUAA
UUAUAGCGUUUCUCACGAUGAUUCGAAAGUGGACAACGUGAGUGAAAUUCAAUGCGCU
UCAAGACCCCCGACAUGGAAUGUUGAACCUGUAGGAUUGGUGCACAUUGGUUUAUCUU
UGAAAAGUCCGUAUUUUCCGAAGAUAGCGAUGUUUGAUUCUACAGUUCAAUGCGUGUC
AAAUAGGAACAUUUUUGUUCCUAAGGCUCUUAAAGAAUUGAACGUUGUUAUUUCUGAG
AGCGCGAUGUUCAUGGUUAUUGGUUCUGUAACAAAACUUUUCAUGAACCAAGCUCCUA
GGAGCGAUAAUUGGUUCAUGAUAAAGUUUUGUCAGUUGAUAGAAAUCUUAGAUCCAAC
AGCGGUAAUAAAUGUUAACAUCGUCAUCAACCAAUCCGACAUAGGUUUGGGAUGGGAU
GGUGACAUGGUGUUGGAUAUUACGCC

>Guadeloupe_mosquito_viruses_strain_AP57_segment1_MT808049
AUUGCAUGUGGCAGUCUCCAAAGGAUUUCUGGCACAUGCAAUCCCGUACAUUGACGCG
UGCUCCGCUAAAAGAAGCUUCCGCACGCACUGUCAGAAACAAAAACAGACGGAUGCGC
CGCCAACAGCAGAAGAAAGAACUAGAGCAGUUCAAGCAGCUCUACGUUCUUCUUCUGC
UUGGCGGCGCAUCCUCUCAUCCGUCGCAGAUCCCCACAAAUGGUUUGAUAGGGAAUUC
AAUAAAGCACUAGAGGAGAUAGAUAUGGAUAGCACUCCUGGCUACUGUAUUUUGACUC
UUUUAGGCUCUACGAAUGCGCAAAUCUUCGGUUGGAAUGGUAUAUGCUGUGAUCCGGA
UAGAGUUAGUAUGGUACGAGAGCAUGUCAAAUUAAGGUUCAAUGAGUUGUUGGCAGG
UAAGGCUGUGAGCGAUUGUAUUAAGGUUUUUGUGAAGCAAGAGCCGCAUAAAAUGAA
GAAAAUUCAGGAAGGUACUUAUCGCUUGAUAAGCGCUGUAAGCUUAAUUGAUACCUUU
AUUGACAGAAUUCUGUUCACUAGGUUAGCCAGAGCUCAACUGGAUUCCCCCGGGGAGA
CGCCAUGUCUCAUCGGGUGGAGUCCAGUUAGAGGCGGAUGGCGUAAGAUAGUGAACAG
GUUUGGCAACCAAGCAGUAAAUUGUUUAGAUAGAUCGGCUUGGGAUUGGACUGUACAA
GGCUAUUUAAUUGAUCUUUGGAUAUUGUUUUUGGAAAAUUUACCAGUGAAUCCUCCAG
ACUGGUGGAUUAAGAAGAUCAAAUUACGUUUUAAGCUACUGUUUGAAGAGGUUUGGU
UUCAGUUCGAAGACGGGACAAGAGUAAAGCAGGCAACGCGAGGAGUAAUGAAGAGUGG

UUGCUACUUAACUAUUUUGUUAAAUAGUCUUAGCCAAAGUCUCUUACAUUACGUAGUC
AACAGUAGAUGUGGAAGCCCAUGACCAUGAAACAACCAUACUCGAUAGGUGAUGACA
CGGUACAGGAAGCUAUGGAUUGGCUUCUAGACUAUGUUAGACAACUCGAGAUAUUGGG
AAUCACGGUCAAAGGAGCUAAAGUCCAGCAUUGGGUUGAAUUUGCGGGGUUCUGUUUU
GACGGGAAAACUUGUUAUCCGGCAUACUGGCAAAAACAUCUUUUUAACCUUAAGUAUA
CGCAAAAUCUUGGGGAGACUCACGAUCUACACACAAAUUCGUACGUCACAGAGCCAUU
GACGUACGAAUUUGUGUGUAGAGUGGCUCGUGAGUUAGGUCCUCGUUUCGUCUUGCCU
AAGAUAGAAGCACUAGACAUAAUCAAUUUGGUUAAUUCAUCCUAUGUCUAGUGCUUCU
AUCUUAUGGCUAAUGGCAACCGAAACGAGUCGACCU

>Guadeloupe_mosquito_viruses_strain_AP57_segment2_MT808050
GCUCUCGAACGGCGCAAUAGUCGCAAGCAGAAACCCGCUCGGGCUACUAGCACGUGCG
AGGUUACUACCUUUGGACGUAAUCUCGUUCAGAGAGCUGAGCUCCUGUUCGAGAUUAA
GGUGGCCAAAGGUAGCUCUUCCACGUCUUAUUAUGAAGCCUUGCAGCCGACCUCGACC
CGAAUGACGUGGCUAAAGACUCUGAGUGGUAGUUUCUCUCAGAUUCUUUGGCAUUCCG
CGAGACUGGAGUAUCGCCCUGCUGUGGGGGCGAUGCAAGAUGGCUCUCUAGUCGUCGG
AGUCGACUGGGACCCUACGACGACCGUGCCUAGUAAAAGCAAGGUCCAAAGUUGUACU
CCUAACUUUCAGGUCCCGGUAUGGCAGAAGAAGGAGUUGACUCUCCCAGCGUCUCGGU
UGCAGAGUCGUAAGUAUUAUGCUCUCUCAGAUUCAGGGGAUAUACCCAUGAUAGCCAA
AACCCCUAGUGAGGCUUUGGCCUAUUUGGCUUGUUCCUCGAAUAAGGAUGAGGAACAG
UACUUUGGAGACAUCUGGAUCCACUAUAAGGUAACCCUGCAGGGGCCUAAAUAGGGAG
ACGAGAUGCUCUCCAAAGUACCUCCAGGACCUAGUGUGUUUGAUUAUCACAUGGUUCG
GGAGGUAACCCUAUAUGAAGUGCGGGCCAUUGUAAGAACUAAAUAUGAGCCCCAACAU
AAACAGACUUCUGGAUAUUAUAAACCCUUUGAGAUCCAGAUGUCGAACAGGGUUAUGC
UAAGAUAUUCAUCAACUGCAAAACUUUCAGUCAAUGGAACCGAUUUAACUAUUGUGGA
UUAUUAUAGCGUUUCUCACGAUGAUUCGAAAGUGGACAACGUGAGUGAAAUUCAAUGC
GCUUCAAUACCCCCGACAUGGAAUGUUGAACCUGUAGGAUUGGUGCACAUUGGUUUAU
CUUUGAAAAGUCCGUAUUUUCCGAAGAUAGCGGGGGUUGAUUUUACGGUUCACUUCGU
UUCAAUUAGGAACAAAAAUGUUCCUAAGGCUCUUGAAGAAAUGAACGUUGCUAUUUCU
GAAAGCGUAAAGAUCAAGAUUAUUGAUCAACUGAUAAAUCUUUUCAAUAGUCAAGCUC
CUAGGAGCGAUAAUUGGUUCAUGAUAAAGUUUUGUCAGUUGAUAGAAAUCUUAGAUCC
AACAGCGGUAAUAAAUGUUAACAUCGUCAUCAACCAAACCUAUGUGGAUUGGAGAUGG
GAUGGUGACAUGGUGUUGGAUAUUACGCCAAUAAUCACGUUCUAUAACUCUGUUACGU
CUCUGUUGCACUUUA

>Guadeloupe_mosquito_viruses_strain_AP59_segment1_MT808051
UACUUUAUUCCUAACUGUAAAACGAGAUGGAAACUGUGAAUGUUAUUUACACCACUGU
GAGUGACGCGGUAGGAUAUAUUUCCAUACCACUGCAGGUAGUCGCCUUAAUGAUGUCA
GCUAUAAUGAUGUCAUUUGUGGUGAACCCGUUGAAUAAAGCCAUGUUGGCGGUAUCCU
UCUACGGGUUCACCACAAAUGACAUCAUUAUAGAUAUGGAGCCUUGCGCCAUCGAUUU
AAGGGAUAAUCCACGAGCGAUUGUCAGAGACGUAUUCUUAAGUGCAUAUGGAAACUUU
GGAAACGUUUUUAUUAGACUAUACGGUCUAUUUAAUGGCGAUAGCAUGUGGUUUUGU
UAUCCUUCUUUCGAGCUAUAUAUAUGUUAUGGUGAGAAAUUGGUGGGCAAAAGACCAC
CGUGUGUUUGUGGUGCCGAAUGGCCAAGCUGGAGCUGGCACUUAUGUCAGGGAGAAAG
CUAUGCCGGGAAGUUCUUUCGAGGUAGCCAAAGUCCUGCCUACUUUCCAGGCUAUGGU
AAUGGCAUCGUUAGAUGGGAUUACUUAUCACGUUAUGGGCCAGUGUUUCUGGGUCGAU
GCAGGCUUAGUGACAGCAGCCCACGUGAUAGAGGGUUUCGAACAAUUGUGUAUCUACA
AGGACGAAGAGAAACGCAUCGAAGUAGGACCAAAAACAUUCGAGAUCGGCAAUGGGGA
UUACGCAGUAUGUCGCAAUCCCACACUGAUAACGCAAAAAUUGGGUUUGAGCAAAGCA

AAGUUUGCGAGGCUGGCGGUCGAAAAAGAUGCCGGCUUGAGUGUCAACAUCACAGCCA
UGGGAAAGAGAACGAUAGGAUUCCUAGAUGGACAUCCACAAUUUGGGUAUGUUCGUUA
UACUGGAAGCACCAUCAAAGGAUUCAGUGGAGCUCCUUACUAUUUUGGCAGGACCAUA
UUUGGAAUGCAUCUGGGGUCGGACUCCAAGAACCUUGGUUAUGCUGGAGCAUUCUUGA
AAACCGAACUCAAGCCCAGUCGGGUUAUAAGAAUUCCAUUAGAACCAAGAACCGAAGA
UCGUGCAAGUUUUCUAGAGCAGCAGUUAGAGCGUUAUCAAGAAGUAGAGUAUUCUCGA
UCUCCGUAAGAUCCCGAAGAAUAGAAGGUCAGGAUUGGAGGGAUGUAUCAUAUUGUCG
AUCAGGAAGUGUUGAGUACCGUCUUAAAGAGAAGAUCCGCCAAGCGAGCACAGGGGGA
UUUACAAUAUUUGGCUGCACAAGCCAAAGUUGGGCCCCUCAUCGAUCUCGAAGCUGUA
ACCACUCAGGGAGACCUUACUGGGUCUCAACAGAGUGGACAAAAACAGCACCAGGAGC
UUUCUACAGAGACGGAUCCGAUAAUAGCCAUGACUCAAGGGCUAUUGUCAGAAUUUGG
CAAAACCUCAGUGUACGUUCCUCCAGAACCUCAGAGUUAAAGACCAAGGGACAUGCAC
UAGUUGUGAGUCUUGUGGCGCCUAUAUAUAUUGGGGGGAGUCUAGGGAUAUUCCUGAA
ACGCCUACUGCGGAUGCCAAGAAAGCGGAAGAUUUACCUUUGGCGCCUAGGAAUGCCA
UGUCGUUUAAUGAUUCGGAAAACCUGCUCAGGGCUCCAGCUGUACUUGUUGGGGCCCG
UGGCAUGGAAUAUCAGAGGCGCUAAAGUGUACAAGAGCCACGACUUGCCAAGAUGCAA
GAAAAUGGAUUGCAUGUGCCAGAAACCUUUGGAGACCUGCCUUAUGGAAUCCCGUACA
UUGACGCGUGCUCCGCCAAAAGAAGCUUCCGCACGCACUGUCAGAAACAAAAACAGAC
GGAUGCGCCGCCAACAGCAGAAGAAAGAACUAGAGCAGUUCAAGCAGCUCUACGGACC
UAUUCCGCAUGGAGGUGUGACCUCACAUCCGCAGCAGAUCCCCACAAAUGGUUUGAUA
GGGAAUUCAAUAAAGCACUAGAGGAGAUAGAUAUGGAUAGCACUCCUGGCUACUGUAU
UUUGACUCUUUUAGGCUCUACGAAUGCGCAAAUCUUCGGUUGGAAUGGUAUAUGCUGU
GAUCCGGAUAGAGUUAGUAUGGUACGAGAGCAUGUCAAAUUAAGGUUCAAUGAGUUG
UUGGCAGGUAAGGCUGUGAGCGAUUGUAUUAAGGUUUUUGUGAAGCAAGAGCCGCAUA
AAAUGAAGAAAAUUCAGGAAGGUACUUAUCGCUUGAUAAGCGCUGUAAGCUUAAUUGA
UACCUUUAUUGACAGAAUUUUGUUCGCUUGGAUAGCCAGAGCUCAGAUGGAUUCCGUC
GGGGAGACGCCAUGUCUCAUCGGGUGGAGUCCAGUUAGAGGCGGAUGGCGUAAGAUAG
UGAACAGGUUUGGCAACCAAGCAGUAAAUUGUUUAGAUAGAUCGGCUUGGGAUUGGAC
UGUACAAGGCUAUGUACUUGAUCUUUGGAUAUUGUUUUUGGAAAAUUUACCAGUGAA
UCCUCCAGACUGGUGGCUUAAGAUGAUGAAACUACGUUUUAAGCUACUGUUUGAAGAG
GUUUGGUUUCAGUUCGAAGACGGAACAAGAGUAAAGCAGGCAACGCGAGGAGUAAUGA
AGAGUGGUUGCUAUUUAACUAUUUUGUUAAAUAGUCUUAGCCAAAGUCUCUUACAUUA
CGUAGUCAACAGUAGAUGUGGGAAGCCCAUGACCAUGAAACAACCAUACUCGAUAGGU
GAUGACACGGUACAGGAAGCUAUGGAUUGGCUUCUAGACUAUGUUAGACAACUCGAGA
UAUUGGGAAUCACGGUCAAAGGAGCUAAAGUCCAGCAUUGGGUUGAAUUUGCGGGGUU
CUGUUUUGACGGGAAAACUUGUUAUCCGGCAAACUGGCAAAAAAGCCUCUACACCGUU
AAGUAUACGCAAAAUCUUGGGGAGACUUUGCGUACUCUACACACAAAUUCGUACGUCA
AUGAGCCUGUGAUGUACGAAUUUGUGUGUAGAGUGGCUCGUGAGUUAGGUCCUCGUUU
CGUCUUGCCUAAGAUAGAAGCACUAGACAUAAUGAAUCAACCAAAUUGAUCCUAUGUC
AAGUGCUUCUAUCUAUCUAAUGGUGAGUGAAACGGGAUGAGUUGGCAAACAAUAGUGG
UUUGCCAACUCAUCCCGUUUCACUCACCAUUAGA

>Guadeloupe_mosquito_viruses_strain_AP59_segment2_MT808052
CCGGGCCGGCGUCUCCUCCUCGUUUCUUAUUGCGCUUAUUGCGCCGUUUCUUAUCAGA
AGCGUUAGGUUGCGGAGUCGUAAGUAUUAUGCUCUCUCAGAUUCAUGGGUAUAUCCCC
UGAAUCUGAGAGAGCAUAAUACUUACGACUCUGCAACCGAGACGCUGGGAGAGUCAAC
UCCUUCUUCUGCCAUACCGGGACCUGAAAGUUAGGAGUACAACUUUGGACCUUGCUUU
UACUAGGCACGGUCGUCGUAGGGUCCCAGUCGACUCCGACGACUAGAGAGCCAUCUUU
CAUCGCCCCCACAGCAGGGCGAUACUCUAGUCUCGCGGAAUGCCAAAUUAUCUGAGAG

AAACUACCACUCAGAGUCUUUAGCCACGUCAUUCGGGUCGAGGUCGGCUGCAAGGCUU
CAUAAUAAGACGUGGAAGAACUACCAUGGGCCACCUUAAUCUCGAACAGGAGCUCAGC
UCUCUGAACGGUAAUACGUCCAUCAGCAGAAACCCCGCUCAGCGGCUCCUCCACGCUUC
UGCUAAGAAACGGCGCAAUAGGCGCAAUAAGAAACGUGGAGGAGCCGCUGGCCCGAGC
GGGGGUUCUGCUGAUGGACGUAUUACCGUUCAGAGAGCUGAGCUCCUGUUCGAGAUUA
AGGUGGCCAAAGGUAGUUCUUCCACGUCUUAUUAUGAAGCCUUGCAGCCGACCUCGAC
CCGAAUGACGUGGCUAAAGACUCUGAGUGGUAGUUUCUCUCAGAGUCUUUGGCCACGU
CAUUCGAGACUAGAGUAUCGCCCUGCUGUGGGGGCGAUGAAAGAUGGCUCUCUAGUCG
UCGGAGUCGACUGGGACCCUACGACGACCGUGCCUAGUAUAGGCAAGGUCUGGGGCUU
UGUCUAUCAUGGGUAUAUCCCAAUCUGGGAGAGCAUAAUACUUACGACUCUGCAACCG
AGACGCUGGGAGAGUCGUAAGUAUUAUGCUCUCUCAGAUUCAGGGGAUAUACCCAUGA
UAGACAAAGCCCCUUGUGAGGUUUUGGCCUAUUUGGCUUGUUCCUCGAAUAAGGAUGA
GGAACAGUACUUUGGAGACAUCUGGAUCCACUAUAAGGUAACCCUGCAGGGGCCUAAA
UAGGGAGACGAGACGCCCCCCCCAUUACCUCCAGGACCUAGUGUGUUUGAUUACCACA
UGGUUCGGGAGGUAACCCUAUAUGAAGUGCGGGCCAUUGUAAGAACUAAAUAUGAGCC
CCAACAUAAACAGACUUCUGGAUAUUAUAAACCUUUUGAGAUCCAGAUGGCGAACAGG
GAUAUGAUAACGUAUGCAGCGGCGGCUGAGGCUACAGGUUCAACAUUCCAUGUCGGGG
GUCUCGAAGCGCAUUGUAAUUUCACUCACGUUGAUUCCACUUUCGACAUCGUGAGUGA
AAUUCAAUGCGCUUCGAGACCCCCGACAUGGAAUGUUGAACCUGUAGGAUUGGUGCAC
AUUGGUUUAUCUUUGAAAAGUCCGUAUUUUCCGAAGAUAGCUAUGUUUGAUUUUACGG
UUCAUUUCGUGUCAAAUAGGAACAUCUUUGUUCCUAAGGCUCUUAAAGAAGUGAACGU
UGCUAUUUCUGAGAGCGCGAAGAUCAAGGUUAUUGGUCCUGUAAUGAAUCUUUUCAAU
AGUCAAGCUCCUAGGAGCGAUAAUUGGUUCAUGAUAAAGUUUUGUCAGUUGAUAGAAA
UCUUAGAUCCAACAGCGGUAAUAAAUGUUAACAUCGUCAUCAACCAAACCUAUGUGGA
UUGGAGAUGGGAUGUUAACAUGCUGUUGGAUCUAAGAUUUCUAUCAACGUGGCAAAAC
UUUAUCAUGAACCAAUUAU

>Guadeloupe_mosquito_viruses_strain_AP60_segment1_MT808053
GUUAUUUACACCACUGUGAGUGACGCGGUAGGAUAUAUUUCCAUACCACUGCAGGUAG
UCGCCUUAAUGAUGUCAGCUAUAAUGAUGUCAUUUGUGGUGAACCCGUUGAAUAAAGC
CAUGUUGGCGGUAUCCUUCUACGUGCAAACCGAACAUGGCUUUAUUCACGGGUUCACC
CAAAUGACAUCGAUUGAAGGGAUAAUCCACGAGCGAUUGUCAGAGACGUAUUCUAAAG
UGCAUAUGGAAACUUUGGAAACGUUUUUAUUGGACUAUACUGCGUAUUCCCCAUUGCC
GAUCUCGAAUGUUUUUGGUCCUACUUCGAGCUAUAUAUAUGUUGAGGUGAGAAAUUGG
UGGGCAAAAGACCACCGUGUGUUUGUGGUGCCUAAUGGCCAAGCUGGAGCUGGCACUU
AUGUCAGUGAGAAAGCUAUGCCGGGAAGUUCUUUUGAGGUAGCUAAAGUCCUGCCUAC
UUUCCAGGCUAUGGUAAUGGCAUCGUUAGAUGGGAUUACCUAUCCCGUAAUGGGCCAG
UGUUUCUGGGUCGAUGAAGGCUUAGUGACAGCAGCCCAGGUGAUUGGGGGUUUUGAAU
AUUUGUGUAUCUACAAGGAUGAAGAGAAACGCAUCGAAGUAGGAUCAAAAACAUUCGA
GAUCGUCAAUGGGGAUUACGCAGUAUGUCGCAAUCCAACACUGAUAACGCAAAAAUUG
GGUUUGAGCAAAGCAAAGUUUGCGAGGCUGGCGGUCGAAAAAGAUGCCGGCUUGAGUG
UUAACAUCACAGCCAUGGGAAAGAGAACGAUAGGAUUCCUAGAUAGACAUCCACAAUU
UGGGUAUGUUCGUUAUAGUGGAAGCACCAUUAAAGGAUUUAGUGGAGCUCCUUACUAU
UUUGGCAGGACCAUAUUUGGAAUGCAUCUAGGGUCCUAUCGUUCUCUUUCCUAUCGUU
CUCUUUCCGCAUUCUUGAAAACUAAACUCAAGCCCAGUCGGGUUAUAAGAAACCAGUU
AAAUUUAAGAACUGAAGAUAGUGCAAGUUUUCUAGAGCAGCAGUUAGAGCGUUAUGA
AGAAGUAGAGUAUUCUAGAUCUCCGUAUAAUCCCGAUGAAUAUAAGGUUAGGAUUGGA
GGGAUGUAUCAUAUUGUUGAUGAGGAAGUGUUGAGUACCGUCUUAAAGAGAAGAUCC
GCUAAGCGAGCACAGGGGGAUCUUCUCUUUAAGACGGUACUCAACACUGAAGUUGGC

CCCUUAUUGAUCUCGAAGCUGUGUCCACUCUGGGAGACCUAGUAAGGUCUCAACAGAG
UGGACAAAAACAGCACCAGGAGCUUUCUACUGAGACGGAUCCGAUAAUAGCUAUGUCU
ACAGGGCUAUUGUCAGAAUUUGGCAAAACCUCAGUGUACGUUCCUCCAGAACCUAAGA
GUAAAGACCAAGGGACAUGCACUAGUUGUGAGUCUUGUGGCGCCUAUAUAUAUUGGGG
GGAGUCUAGGGAUAUUCCUGAAACGCCUACUGCGGAUGCCAAGAAAGCGGAAGAUUUA
CCUUUAGCGCCUAGGAAUGCCAUGACGUUUAAUGAUUCGGAAAACCUGAUCAGGGCUC
CAGCUGUCCGCUUUCUUGGCAUCCGCAGUAGGGAAUAUCAGAGGGAAAGUGUACAGAG
CCACGACUUGCCAAGAUGCAAGAAAAUGGAUUGCAUGUGCCAGAAACCUUUGGAGACC
UGCCUUAUGGAAUCCCGUACAUUGACGCGUGCUCCGCUAAAAGAAGCUUCCGCACGCA
CUGUCAGAAACAAAAACAGACGGAUGCGCCGCCAACAGCAGAAGAAAGAACUAGAGCA
GUUCAAGCAGCUCUACGGACCUAUUCCGCAUGGAGGUGUGACCUCACAUCCGCAGCAG
AUCCCCACAAAUGGUUUGAUAGGGAAUUCAAUAAAGCACUAGAGGAGAUAGAUAUGGA
UAGCACUCCUGGCUACUGUAUUUUGACUCUUUUAGGCUCUACGAAUGCGCAAAUCUUC
GGUUGGAAUGGUAUAUGCUGUGAUCCGGAUAGAGUUAGUAUGGUACGAGAGCAUGUC
AAAUUAAGGUUCAAUGAGUUGUUGGCAGGUAAGGCUGUGAGCGAUUGUAUUAAGGUU
UUUGUGAAGCAAGAGCCGCAUACAAUGAACAAAAUUCUGAACAGCGCUUAUCAAGCGA
UAAGCGCUGUAAGCUUAAUUGAUACCUUUAUUGACAGAAUUUUGUUCGCUUGGAUAGC
CAGAGCUCAGAUGGAUUCCGUCGGGGAGACGCCAUGUCUCAUCGGGUGGAGUCCAGUU
AGAGGCGGAUGGCGUAAGAUAGUGAACAGGUUUGGCAACCAAGCAGUAAAUUGUUUAG
AUAGAUCGGCUUGGGAUUGGACUGUACAAGGCUAUUUAGUUGAUCUUUGGAUAUUGU
UUUUGGAAAAUUUACCAGUGAAUCCUCCAGACUGGUGGCUUAAGAUGAUGAAACUACG
UCUUAAGCUACUGUUUGAAGAGGUUUGGUUUCAGUUCGAAGACGGAACAAGAGUAAAG
CAGGCAACGCGAGGAGUAAUGAAGAGUGGUUGCUAUUUAACAAAAUUGUUAAAUAGUC
UUAGCCAAAGUCUCUUACAUUACGUAGUCAACAGUAGAUGUGGGAAGCCCAUGACCAU
GAAACAACCAUACUCGAUAGGUGAUGACACGGUACAGGAAGCUAUGGAUUGGCUUCUA
GACUAUGUUAGACAACUCGAGAUAUUGGGAAUCACGGUCAAAGGAGCUAAAGUCCAGC
AUUGGGUUGAAUUUGCGGGGUUCUGUUUUGACGGGAAAACUUGUUAUCCGGCAUACUG
GCAAAAACAUCUUUUUAACCUUAAGUAUACGCAAAAUCUUGGGGAGACUUUGCGUAGU
CUACACACAAAUUCGUACGUCAAUGAGCCUGUGAUGUACGAAUUUGUGUGUAGAGUGG
CUCGUGAGUUAGGUCCUCGUUUCGUCUUGCCUAAGAUAGAAGCACUAGACAUAAUSAA
USGACCUAACUCACGAGCCACUCUACAAGUUCUCUUCUAUCUAAUGGUGAGUGAAACG
GGAUGAGUUGGCAAACAAUA

>Guadeloupe_mosquito_viruses_strain_AP60_segment2_MT808054
AAAAUAAGCAAUUUAACUCACUUGAAUGUAAGUGCAGCAACCUAAGGAAGCAUAUCAU
CUACCUUACUUUAAUUUCUGUUAUUUUAACACAAUGAGUAAACAGUUGAAGAAACAGC
UUGCAUCUUUAACUGCUCAAGUAGCUGCGAUGAGGGUUGCCAAACCGCAACCUAACGC
UUCUGCUAAGAAACGGCGCAAUAGGCGCAAUAAGAAACGUGGAGGAGCCGACGGCCCG
AGCGGGGUUUCUGCUGAUGGACGUAUUACCGUUCAGAGAGCUGAGCUCCUGUUCGAGA
UUAAGGUGGCCAAAGGUACUUUUACUAGGCACGGUCGCGUAGGGUCCCAGCCGACCUC
GACCCGAAUGACGUGGCUAAAGUCUCUGAGUGGUAGUUUCUCUCAGAUAAUUUGGCAU
UCCGCGAGACUAGAGUAUCGCCCUGCUGUGGGGGCGAUGAAAGAUGGCUCUCUAGUCG
UCGGAGUCGACUGGGACCCUACGACGACCGUGCCUAGUAAAAGCAAGGUCCAAAGUUG
UACUCCUAACUUUCAGGUCCCGGUAUGGCAGAAGAAGGAGUUGACUCUCCCAGCGUCU
CGGUUGCAGAGUCGUAAGUAUUAUGGUCUCUCAGAUUCAGGGGGUAUACCUGCGGUAG
ACAGAUCGGAGGGUGCGGGGGACGCCUACUUGGCUUGUUCCUCGAAUAAGGAUGAGGA
ACAGUACUUCGGAGACAUCUGGAUCCACUAUAAGGUAACCCUGCAGGGGCCUAAAUAG
GGAGACGAGACGCCCCCCCCAUCGCCUCCAGGACCUAGUGUGUUUGAUUACCACAUGGU
UCGGGAGGUAACCCUAUAUGAAGUGCGGGCCAUUGUAAGAACUAAAUAUGAGCCCCAA

CAUAAACAGACUUCUGGAUAUUAUAAACCUUUUGAGAUCCAGAUGCUGAACUGGGAUA
UGAUAACGUAUGCAGCGGCGGCUGAGGUUUUAGUCAAUGGAACCGAUUUAACUAUUGA
GGAUAAUUAUAGCGUUUCUCACGAUGAUUCGAAAGUUGACAACUUGCUUGAAAGCCUA
GGAACAAAAAUGUUCCUGACAUGGAAUGUUGAACCUGUAGGAUUGGUGCACAUUGGUU
UAUCUUUGAAAAGUCCGUAUUUUCCGAAGAUAGCGAUGUUUGAUUUUACGGUUCAUUU
CGUGUCAAAUAGGAACAUUUUUGUUCCUAAGGCUCUUAAAGAAGUGAACGUUGCUAUU
UCUGAGAGCGUAAAGAUCAAGGUUAUUGGUCAACUGAUAAAUCUUUUCAAUAGUCAAG
CUCCUGCUAUAGGAAAUAGGUUCUUGAUUUCGCGCUCUCAGUUAAUAGGAACGUUACU
UCUAAGAGCGGAUAUAGACGCUAAGAUCGUCAUAAACUACGUCUCUGUGCAUUGGUGG
AUGACG
