# Supplementary material for: Aedes aegypti from Amazon Basin Harbor High Diversity of Novel Viral Species
Source: Viruses. 2020 Aug 8;12(8):866. doi: 10.3390/v12080866 (PMC7472207; doi:10.3390/v12080866)
Supplement: Supplementary file 1 [file viruses-12-00866-s001.zip › viruses-862891-supplementary/viruses-862891supplementaryFile2forXML.pdf]

viruses

MDPI

Supplementary materials

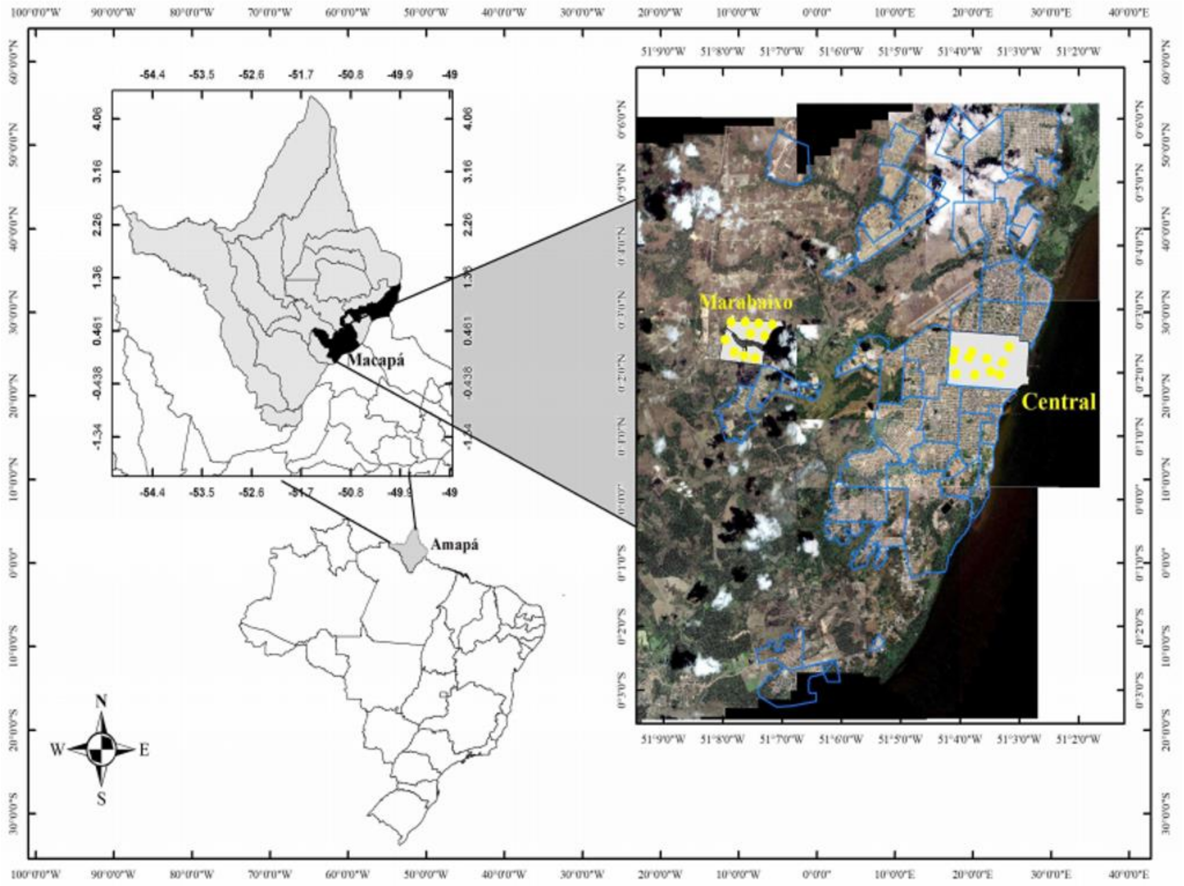

Figure S1. Map showing the coordinates where mosquitoes were captured in Brazil. Amapa state is shown in the north of Brazil. The locations of 21 points where samples were collected were shown in an aerial view in the vicinity of Macapa city.

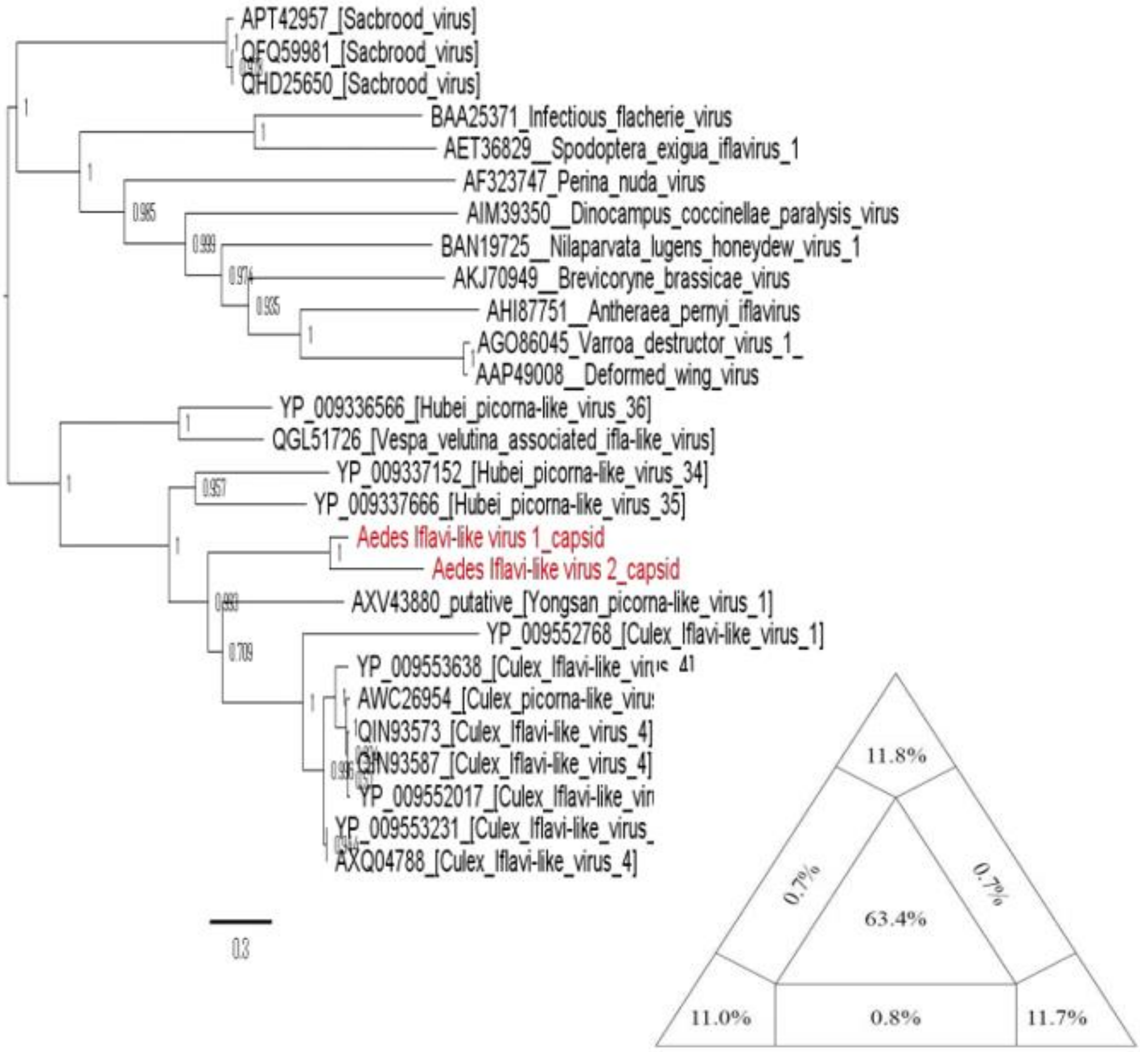

Figure S2. Phylogenetic tree of Iflavi-like virus and Iflavirus genus members based on the alignment of capsid region protein. Aedes Iflavi-like virus 1 and Aedes Iflavi-like virus 2 are highlighted in red. This tree was made using the maximum likelihood methods assuming VT model and values on the nodes of the tree indicate the statistical support based on the approximate likelihood-ratio text (aLRT). The diagram in the base of the tree is a likelihood map of the nucleotide alignment of the genomes of Iflavi-related and iflaviviruses. The likelihood quartet mapping is a method that allows to visualize the tree-likeness of all quartets in a single graph and provides a direct measure of the phylogenetic signal in an alignment. The triangle shows the locations of all quartets calculated with the alignment used to infer the ML tree. Values in the center of the triangle represent the percentage of unresolved quartet trees (star-like trees), values in the vertices represent the percentage of fully resolved trees and values in the intermediate areas (between vertices) are the percentage of conflicting trees. The analysis was performed using JTT model as is implemented in the tree puzzle software v 5.3 (https://www.tree-puzzle.de).

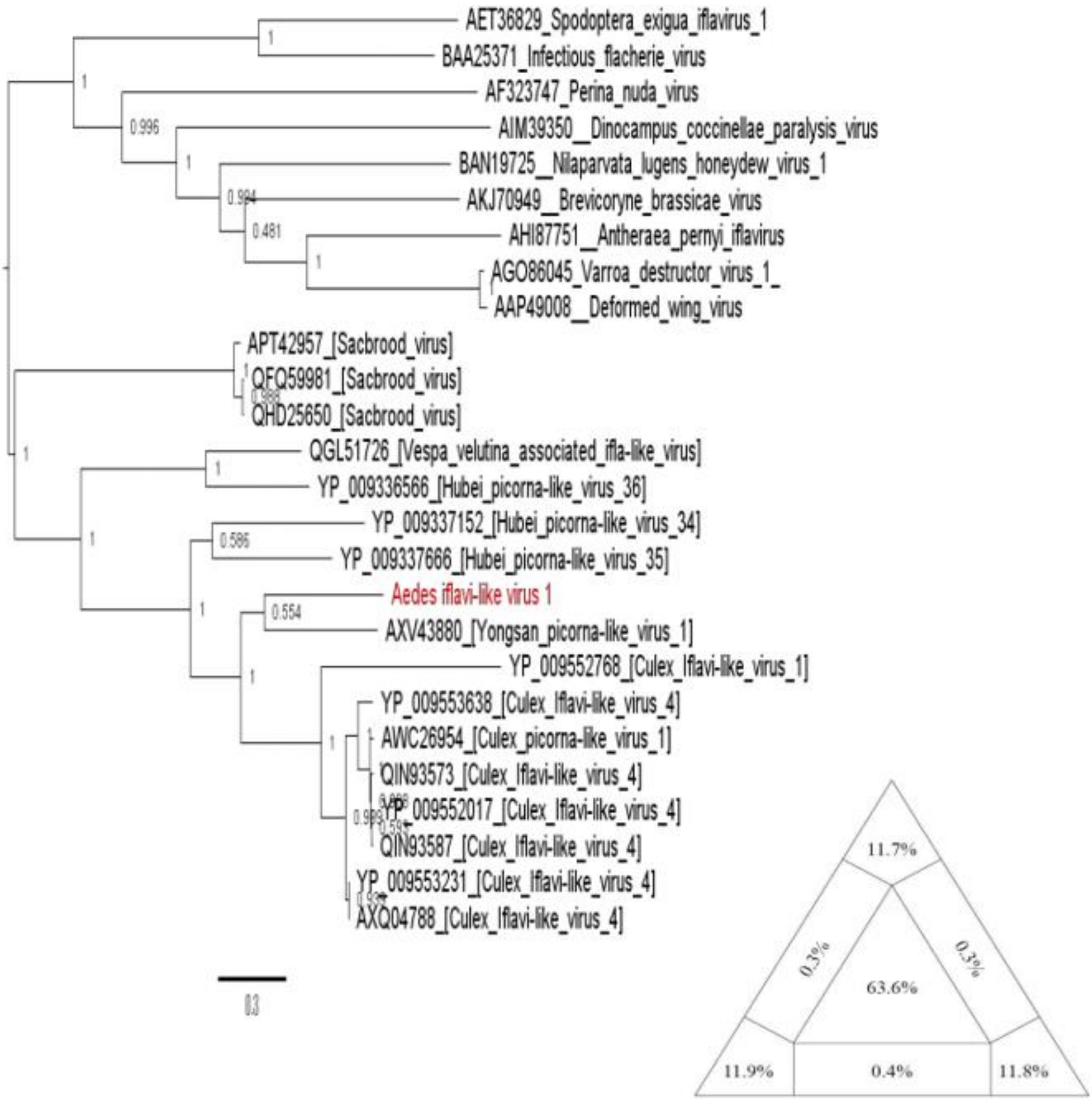

Figure S3. Phylogenetic tree of Iflavi-like virus and Iflavirus genus members based on the alignment of helicase region protein. Aedes Iflavi-like virus 1 is highlighted in red. This tree was made using the maximum likelihood methods assuming VT model and values on the nodes of the tree indicate the statistical support based on the approximate likelihood-ratio test (aLRT). The diagram in the base of the tree is the a likelihood map of the nucleotide alignment of genomes of Iflavi-like virus and Iflavirus. The likelihood quartet mapping is a method that allows to visualize the tree-likeness of all quartets in a single graph and provides a direct measure of the phylogenetic signal in an alignment. The triangle shows the location of all quartets calculated with the alignment used to infer the ML tree. Values in the center of the triangle represent the percentage of unresolved quartet trees (star-like trees), values in the vertices represent the percentage of fully resolved trees and values in the intermediate areas (between vertices) are the percentage of conflicting trees. The analysis was performed using JTT model as implemented in the tree puzzle software v 5.3 (https:/www.tree-puzzle.de).

FIGURE s4

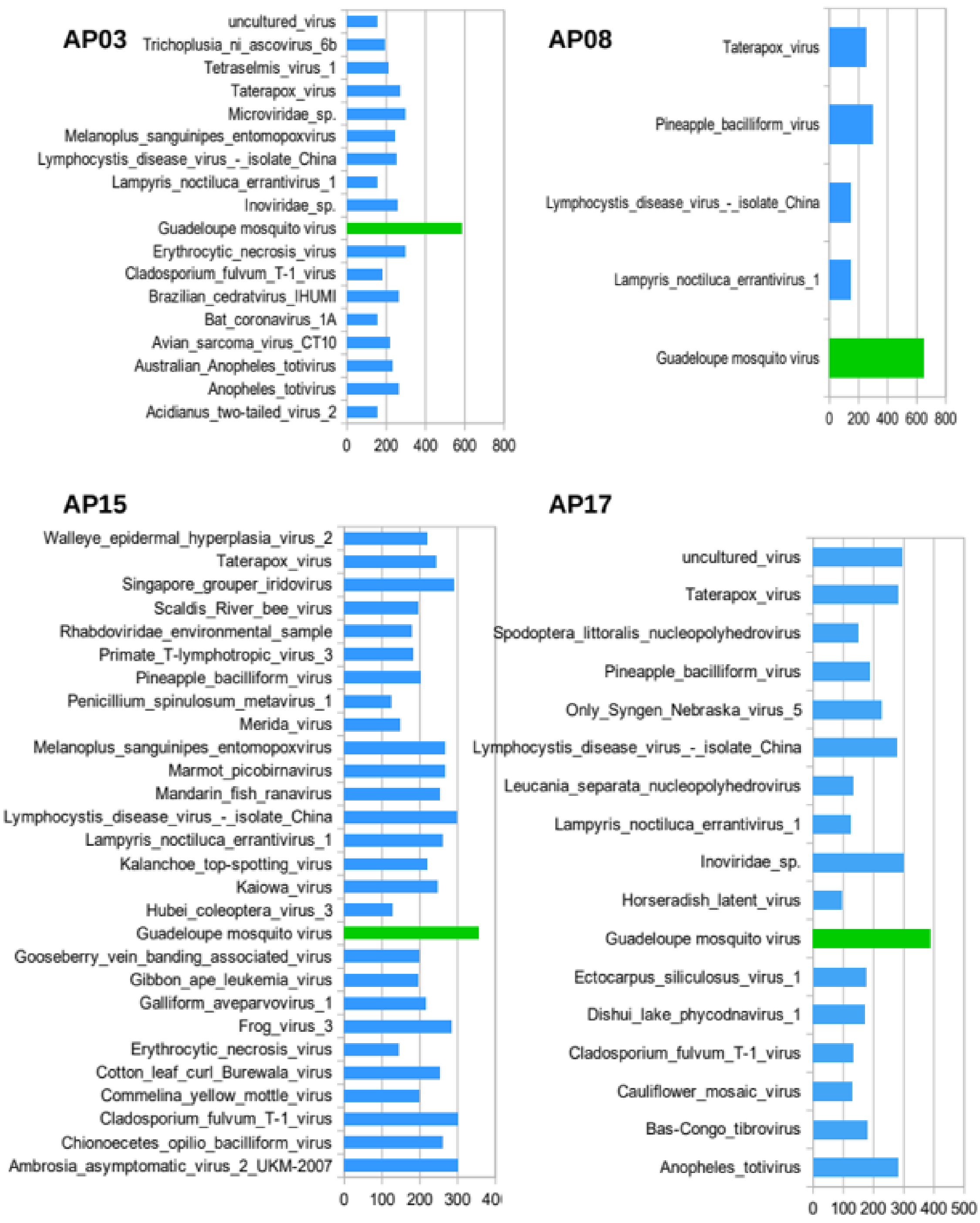
AP03
uncultured_virus
Trichoplusia_ni_ascovirus_6b
Tetraselmis_virus_1
Taterapox_virus
Microviridae_sp.
Melanoplus_sanguinipes_entomopoxvirus
Lymphocystis_disease_virus_-_isolate_China
Lampyris_noctiluca_errantivirus_1
Inoviridae_sp.
Guadeloupe mosquito virus
Erythrocytic_necrosis_virus
Cladosporium_fulvum_T-1_virus
Brazilian_cedratvirus_IHUMI
Bat_coronavirus_1A
Avian_sarcoma_virus_CT10
Australian_Anopheles_totivirus
Anopheles_totivirus
Acidianus_two-tailed_virus_2
0 200 400 600 800
AP08
Taterapox_virus
Pineapple_bacilliform_virus
Lymphocystis_disease_virus_-_isolate_China
Lampyris_noctiluca_errantivirus_1
Guadeloupe mosquito virus
0 200 400 600 800
AP15
Walleye_epidermal_hyperplasia_virus_2
Taterapox_virus
Singapore_grouper_iridovirus
Scaldis_River_bee_virus
Rhabdoviridae_environmental_sample
Primate_T-lymphotropic_virus_3
Pineapple_bacilliform_virus
Penicillium_spinulosum_metavirus_1
Merida_virus
Melanoplus_sanguinipes_entomopoxvirus
Marmot_picobirnavirus
Mandarin_fish_ranavirus
Lymphocystis_disease_virus_-_isolate_China
Lampyris_noctiluca_errantivirus_1
Kalanchoe_top-spotting_virus
Kaiowa_virus
Hubei_coleoptera_virus_3
Guadeloupe mosquito virus
Gooseberry_vein_banding_associated_virus
Gibbon_ape_leukemia_virus
Galliform_aveparvovirus_1
Frog_virus_3
Erythrocytic_necrosis_virus
Cotton_leaf_curl_Burewala_virus
Commelina_yellow_mottle_virus
Cladosporium_fulvum_T-1_virus
Chionoecetes_opilio_bacilliform_virus
Ambrosia_asymptomatic_virus_2_UKM-2007
0 100 200 300 400
AP17
uncultured_virus
Taterapox_virus
Spodoptera_littoralis_nucleopolyhedrovirus
Pineapple_bacilliform_virus
Only_Syngen_Nebraska_virus_5
Lymphocystis_disease_virus_-_isolate_China
Leucania_separata_nucleopolyhedrovirus
Lampyris_noctiluca_errantivirus_1
Inoviridae_sp.
Horseradish_latent_virus
Guadeloupe mosquito virus
Ectocarpus_siliculosus_virus_1
Dishui_lake_phycodnavirus_1
Cladosporium_fulvum_T-1_virus
Cauliflower_mosaic_virus
Bas-Congo_tibrovirus
Anopheles_totivirus
0 100 200 300 400 500

FIGURE s5

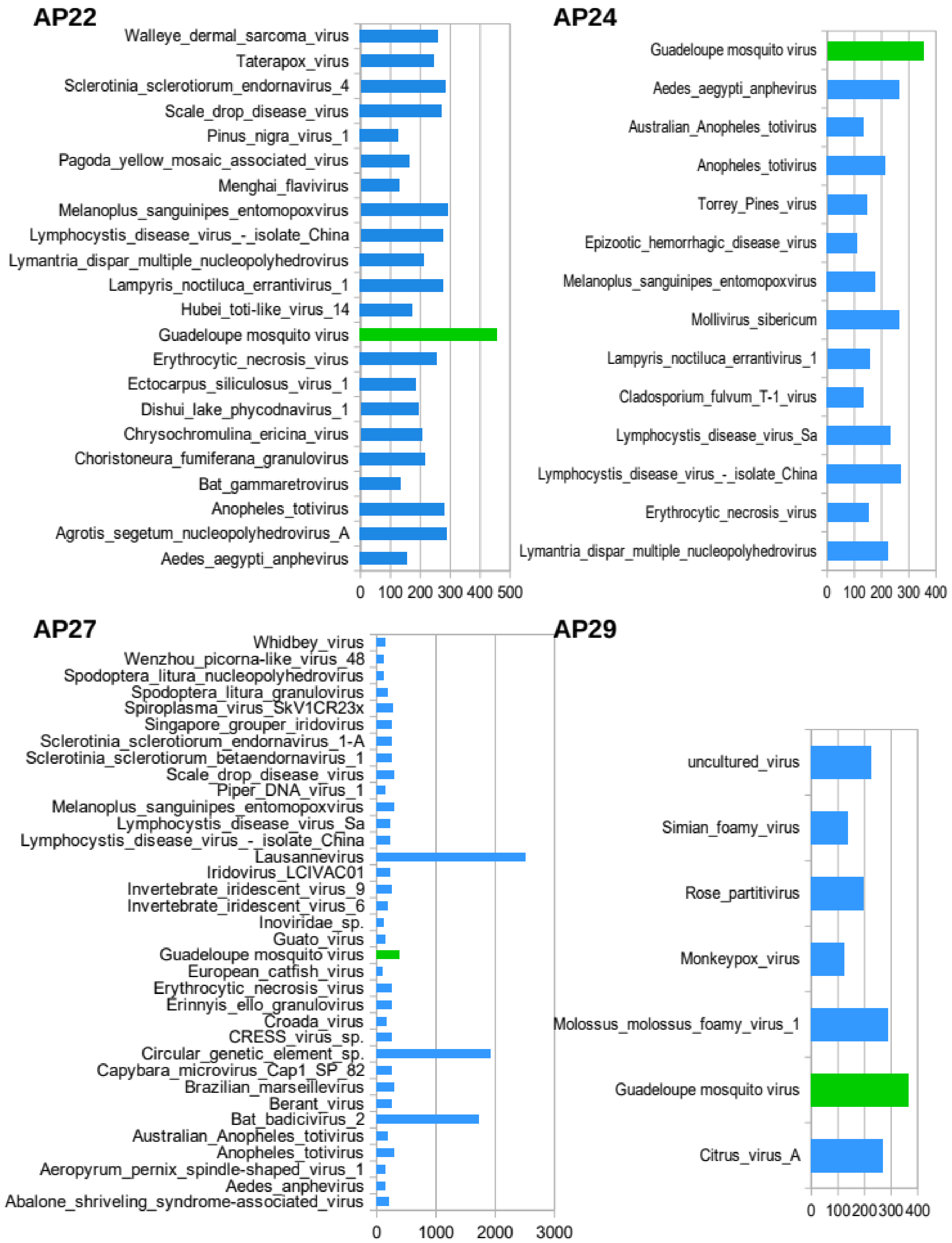
AP22
Walleye_dermal_sarcoma_virus
Taterapox_virus
Sclerotinia_sclerotiorum_endornavirus_4
Scale_drop_disease_virus
Pinus_nigra_virus_1
Pagoda_yellow_mosaic_associated_virus
Menghai_flavivirus
Melanoplus_sanguinipes_entomopoxvirus
Lymphocystis_disease_virus_-_isolate_China
Lymantria_dispar_multiple_nucleopolyhedrovirus
Lampyris_noctiluca_errantivirus_1
Hubei_toti-like_virus_14
Guadeloupe mosquito virus
Erythrocytic_necrosis_virus
Ectocarpus_siliculosus_virus_1
Dishui_lake_phycodnavirus_1
Chrysochromulina_ericina_virus
Choristoneura_fumiferana_granulovirus
Bat_gammaretrovirus
Anopheles_totivirus
Agrotis_segetum_nucleopolyhedrovirus_A
Aedes_aegypti_anphevirus
0 100 200 300 400 500
AP24
Guadeloupe mosquito virus
Aedes_aegypti_anphevirus
Australian_Anopheles_totivirus
Anopheles_totivirus
Torrey_Pines_virus
Epizootic_hemorrhagic_disease_virus
Melanoplus_sanguinipes_entomopoxvirus
Mollivirus_sibericum
Lampyris_noctiluca_errantivirus_1
Cladosporium_fulvum_T-1_virus
Lymphocystis_disease_virus_Sa
Lymphocystis_disease_virus_-_isolate_China
Erythrocytic_necrosis_virus
Lymantria_dispar_multiple_nucleopolyhedrovirus
0 100 200 300 400
AP27
Whidbey_virus
Wenzhou_picorna-like_virus_48
Spodoptera_litura_nucleopolyhedrovirus
Spodoptera_litura_granulovirus
Spiroplasma_virus_SkV1CR23x
Singapore_grouper_iridovirus
Sclerotinia_sclerotiorum_endornavirus_1-A
Sclerotinia_sclerotiorum_betaendornavirus_1
Scale_drop_disease_virus
Piper_DNA_virus_1
Melanoplus_sanguinipes_entomopoxvirus
Lymphocystis_disease_virus_Sa
Lymphocystis_disease_virus_-_isolate_China
Lausannevirus
Iridovirus_LCIVAC01
Invertebrate_iridescent_virus_9
Invertebrate_iridescent_virus_6
Inoviridae_sp.
Guato_virus
Guadeloupe mosquito virus
European_catfish_virus
Erythrocytic_necrosis_virus
Erinnyis_ello_granulovirus
Croada_virus
CRESS_virus_sp.
Circular_genetic_element_sp.
Capybara_microvirus_Cap1_SP_82
Brazilian_marseillevirus
Berant_virus
Bat_badicivirus_2
Australian_Anopheles_totivirus
Anopheles_totivirus
Aeropyrum_pernix_spindle-shaped_virus_1
Aedes_anphevirus
Abalone_shriveling_syndrome-associated_virus
0 1000 2000 3000
AP29
uncultured_virus
Simian_foamy_virus
Rose_partitivirus
Monkeypox_virus
Molossus_molossus_foamy_virus_1
Guadeloupe mosquito virus
Citrus_virus_A
0 100 200 300 400

FIGURE s6

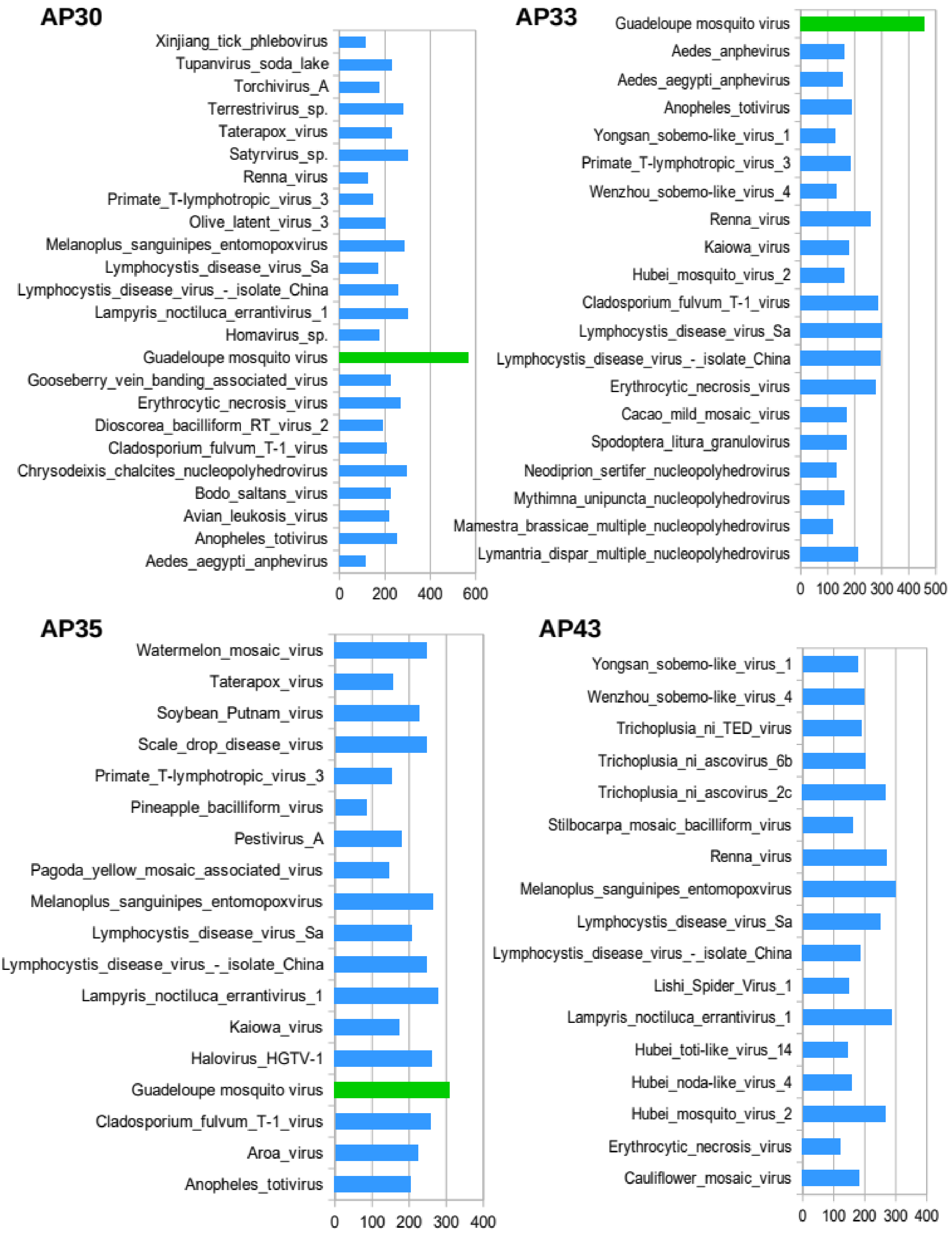
AP30
Xinjiang_tick_phlebovirus
Tupanvirus_soda_lake
Torchivirus_A
Terrestrivirus_sp.
Taterapox_virus
Satyrvirus_sp.
Renna_virus
Primate_T-lymphotropic_virus_3
Olive_latent_virus_3
Melanoplus_sanguinipes_entomopoxvirus
Lymphocystis_disease_virus_Sa
Lymphocystis_disease_virus_-_isolate_China
Lampyris_noctiluca_errantivirus_1
Homavirus_sp.
Guadeloupe mosquito virus
Gooseberry_vein_banding_associated_virus
Erythrocytic_necrosis_virus
Dioscorea_bacilliform_RT_virus_2
Cladosporium_fulvum_T-1_virus
Chrysodeixis_chalcites_nucleopolyhedrovirus
Bodo_saltans_virus
Avian_leukosis_virus
Anopheles_totivirus
Aedes_aegypti_anphevirus
0 200 400 600
AP33
Guadeloupe mosquito virus
Aedes_anphevirus
Aedes_aegypti_anphevirus
Anopheles_totivirus
Yongsan_sobemo-like_virus_1
Primate_T-lymphotropic_virus_3
Wenzhou_sobemo-like_virus_4
Renna_virus
Kaiowa_virus
Hubei_mosquito_virus_2
Cladosporium_fulvum_T-1_virus
Lymphocystis_disease_virus_Sa
Lymphocystis_disease_virus_-_isolate_China
Erythrocytic_necrosis_virus
Cacao_mild_mosaic_virus
Spodoptera_litura_granulovirus
Neodiprion_sertifer_nucleopolyhedrovirus
Mythimna_unipuncta_nucleopolyhedrovirus
Mamestra_brassicae_multiple_nucleopolyhedrovirus
Lymantria_dispar_multiple_nucleopolyhedrovirus
0 100 200 300 400 500
AP35
Watermelon_mosaic_virus
Taterapox_virus
Soybean_Putnam_virus
Scale_drop_disease_virus
Primate_T-lymphotropic_virus_3
Pineapple_bacilliform_virus
Pestivirus_A
Pagoda_yellow_mosaic_associated_virus
Melanoplus_sanguinipes_entomopoxvirus
Lymphocystis_disease_virus_Sa
Lymphocystis_disease_virus_-_isolate_China
Lampyris_noctiluca_errantivirus_1
Kaiowa_virus
Halovirus_HGTV-1
Guadeloupe mosquito virus
Cladosporium_fulvum_T-1_virus
Aroa_virus
Anopheles_totivirus
0 100 200 300 400
AP43
Yongsan_sobemo-like_virus_1
Wenzhou_sobemo-like_virus_4
Trichoplusia_ni_TED_virus
Trichoplusia_ni_ascovirus_6b
Trichoplusia_ni_ascovirus_2c
Stilbocarpa_mosaic_bacilliform_virus
Renna_virus
Melanoplus_sanguinipes_entomopoxvirus
Lymphocystis_disease_virus_Sa
Lymphocystis_disease_virus_-_isolate_China
Lishi_Spider_Virus_1
Lampyris_noctiluca_errantivirus_1
Hubei_toti-like_virus_14
Hubei_noda-like_virus_4
Hubei_mosquito_virus_2
Erythrocytic_necrosis_virus
Cauliflower_mosaic_virus
0 100 200 300 400

FIGURE s7

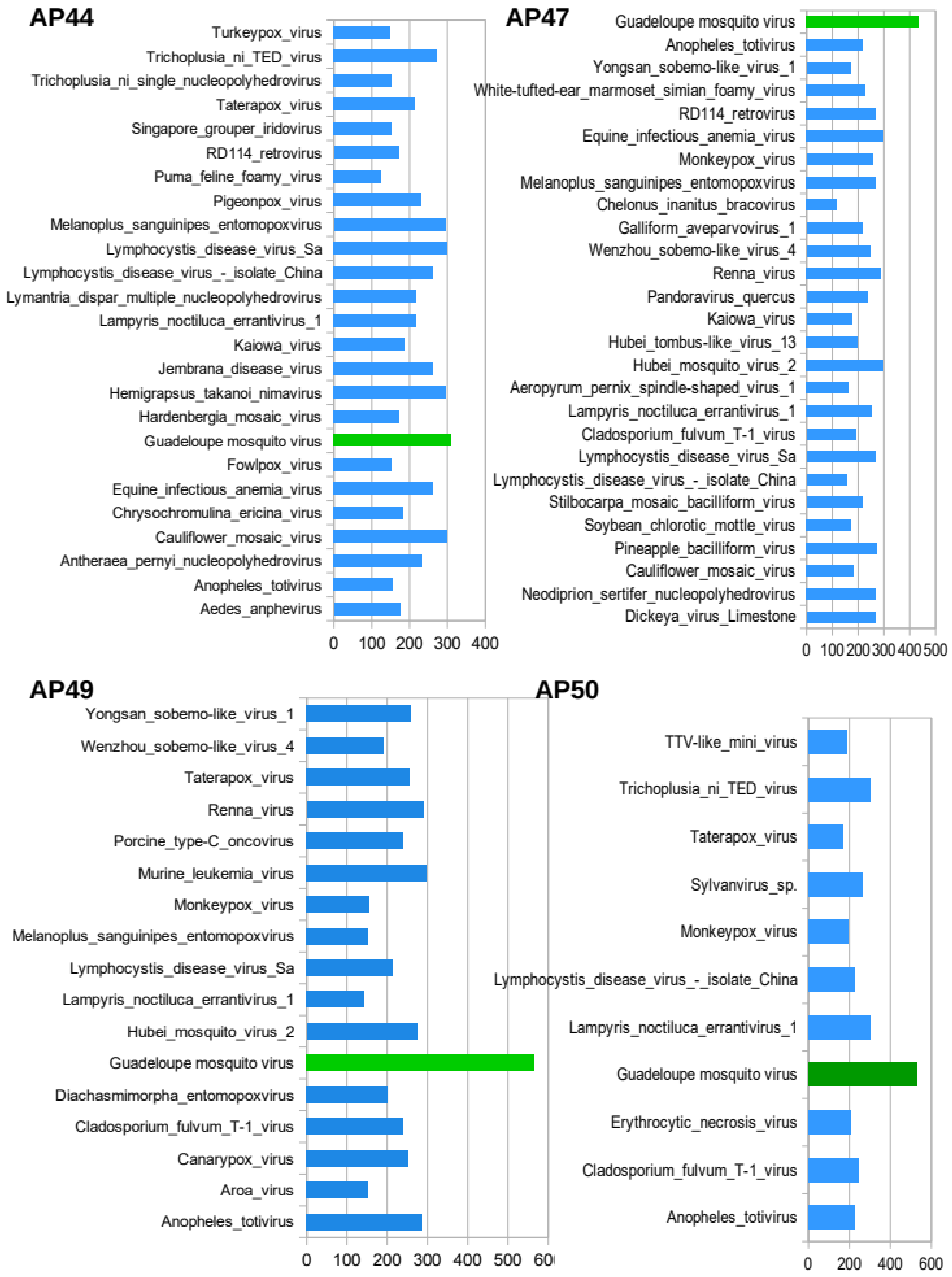
AP44
Turkeypox_virus
Trichoplusia_ni_TED_virus
Trichoplusia_ni_single_nucleopolyhedrovirus
Taterapox_virus
Singapore_grouper_iridovirus
RD114_retrovirus
Puma_feline_foamy_virus
Pigeonpox_virus
Melanoplus_sanguinipes_entomopoxvirus
Lymphocystis_disease_virus_Sa
Lymphocystis_disease_virus_-_isolate_China
Lymantria_dispar_multiple_nucleopolyhedrovirus
Lampyris_noctiluca_errantivirus_1
Kaiowa_virus
Jembrana_disease_virus
Hemigrapsus_takanoi_nimavirus
Hardenbergia_mosaic_virus
Guadeloupe mosquito virus
Fowlpox_virus
Equine_infectious_anemia_virus
Chrysochromulina_ericina_virus
Cauliflower_mosaic_virus
Antheraea_pernyi_nucleopolyhedrovirus
Anopheles_totivirus
Aedes_anphevirus
0 100 200 300 400
AP47
Guadeloupe mosquito virus
Anopheles_totivirus
Yongsan_sobemo-like_virus_1
White-tufted-ear_marmoset_simian_foamy_virus
RD114_retrovirus
Equine_infectious_anemia_virus
Monkeypox_virus
Melanoplus_sanguinipes_entomopoxvirus
Chelonus_inanitus_bracovirus
Galliform_aveparvovirus_1
Wenzhou_sobemo-like_virus_4
Renna_virus
Pandoravirus_quercus
Kaiowa_virus
Hubei_tombus-like_virus_13
Hubei_mosquito_virus_2
Aeropyrum_pernix_spindle-shaped_virus_1
Lampyris_noctiluca_errantivirus_1
Cladosporium_fulvum_T-1_virus
Lymphocystis_disease_virus_Sa
Lymphocystis_disease_virus_-_isolate_China
Stilbocarpa_mosaic_bacilliform_virus
Soybean_chlorotic_mottle_virus
Pineapple_bacilliform_virus
Cauliflower_mosaic_virus
Neodiprion_sertifer_nucleopolyhedrovirus
Dickeya_virus_Limestone
0 100 200 300 400 500
AP49
Yongsan_sobemo-like_virus_1
Wenzhou_sobemo-like_virus_4
Taterapox_virus
Renna_virus
Porcine_type-C_oncovirus
Murine_leukemia_virus
Monkeypox_virus
Melanoplus_sanguinipes_entomopoxvirus
Lymphocystis_disease_virus_Sa
Lampyris_noctiluca_errantivirus_1
Hubei_mosquito_virus_2
Guadeloupe mosquito virus
Diachasmimorpha_entomopoxvirus
Cladosporium_fulvum_T-1_virus
Canarypox_virus
Aroa_virus
Anopheles_totivirus
0 100 200 300 400 500 600
AP50
TTV-like_mini_virus
Trichoplusia_ni_TED_virus
Taterapox_virus
Sylvanvirus_sp.
Monkeypox_virus
Lymphocystis_disease_virus_-_isolate_China
Lampyris_noctiluca_errantivirus_1
Guadeloupe mosquito virus
Erythrocytic_necrosis_virus
Cladosporium_fulvum_T-1_virus
Anopheles_totivirus
0 200 400 600

FIGURE s8

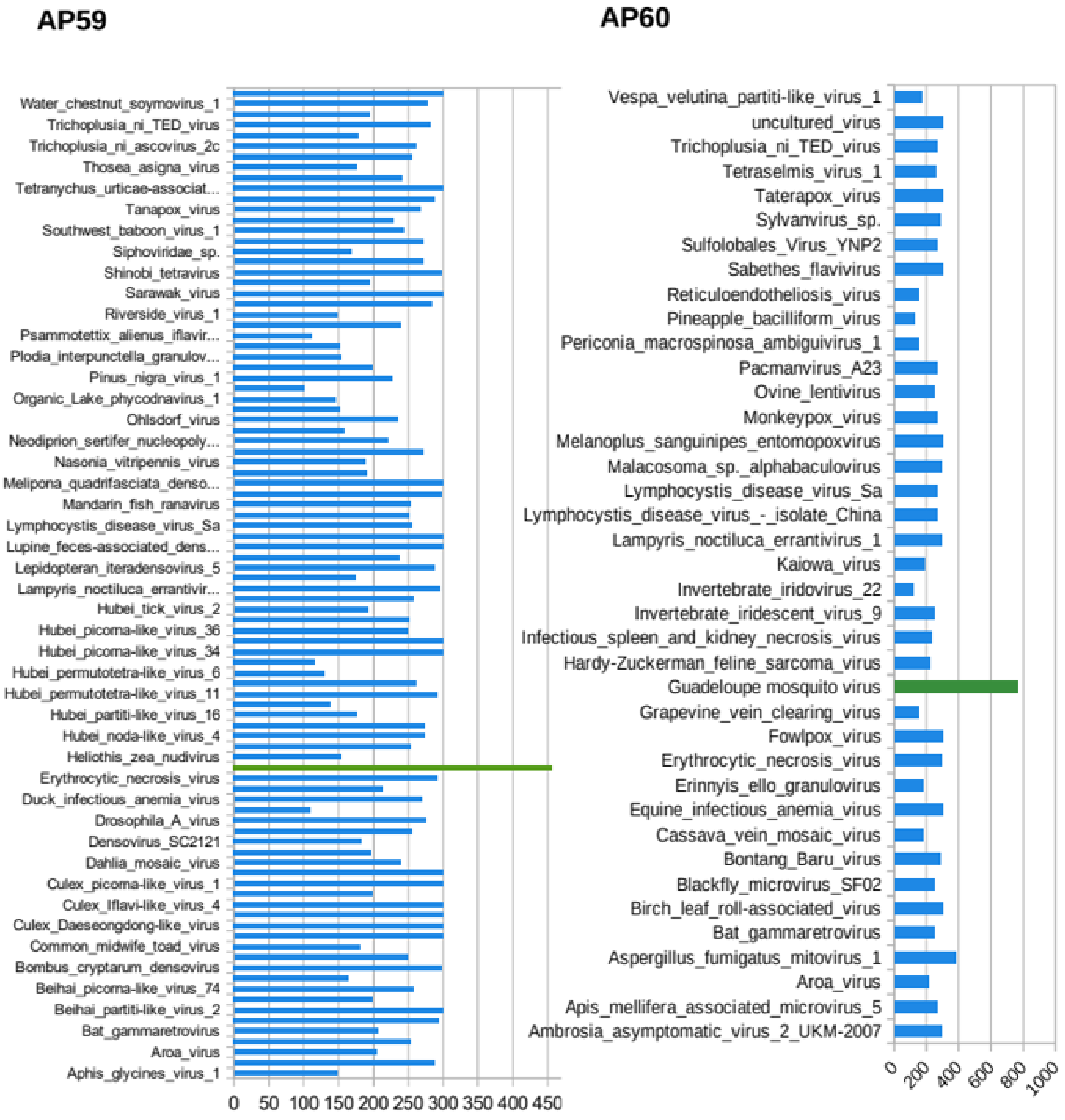

Figures S4-S8. Virome composition of Aedes mosquitoes. On the *y*-axis horizontal bars indicate all viruses detected in the pools of mosquitoes collected in Amapa. On the *x*-axis the number of contigs of each virus found in each library is shown. The green bars indicate the number of contigs of the Guadeloupe virus detected in the libraries. Data was generated using the next generation sequencing of fecal samples (see the main text for details.).

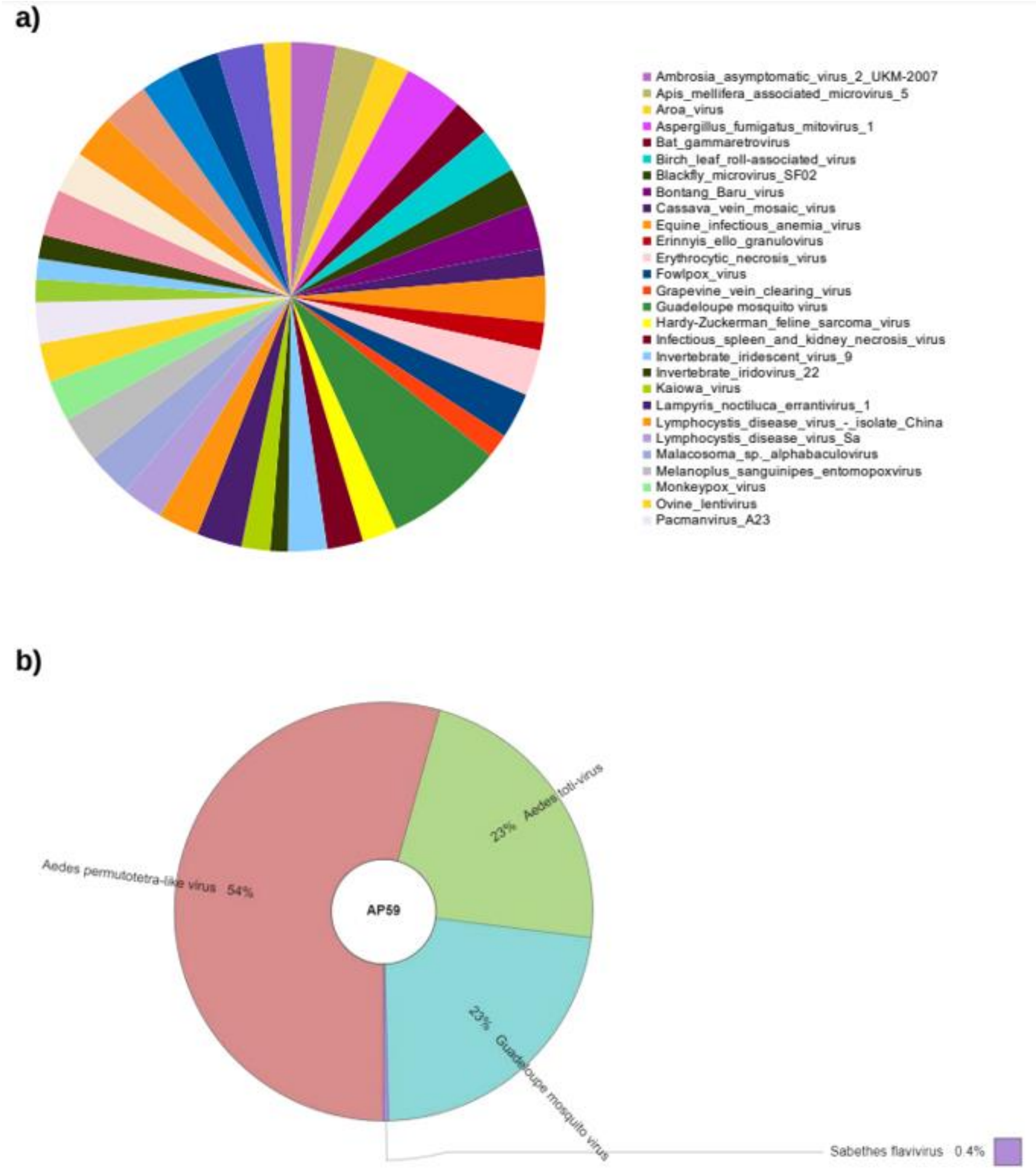

Figure S9. Comparison of viral richness of pool AP59 of Aedes mosquitoes. (**A**) Viruses detected by our pipeline (described in the main text), with the size of each area corresponding to the maximum size of contigs of a certain virus. (**B**) Viral composition determined by KRAKEN software, using adaptable *k*-mers as implemented in the Galaxy web server (https://usegalaxy.org/)

Table S1. Characteristics of pool samples of Aedes aegypti from Macapa.

Sample ID	Location in the Amapa city	Week of sampling in 2017	Intestinal content of females	Number of insects/pool
1	Central	44	empty	1
2	Marabaixo	44	empty	1
3	Marabaixo	30	full	1
4	Marabaixo	30	empty	2
5	Marabaixo	18	empty	1
6	Marabaixo	18	empty	1
7	Marabaixo	18	full	2
8	Marabaixo	18	empty	1
9	Marabaixo	18	full	1
10	Marabaixo	18	empty	3
11	Marabaixo	18	full	1
12	Marabaixo	18	empty	1
13	Marabaixo	18	full	1
14	Marabaixo	34	empty	2
15	Marabaixo	34	empty	1
16	Marabaixo	34	empty	2
17	Central	34	full	1
18	Central	12	full	2
19	Central	12	empty	1
20	Central	12	full	1
21	Marabaixo	14	full	1
22	Marabaixo	14	empty	1
23	Marabaixo	14	empty	1
24	Marabaixo	14	empty	1
25	Marabaixo	14	empty	2
26	Marabaixo	14	full	1
27	Marabaixo	14	full	2
28	Marabaixo	24	full	3
29	Marabaixo	24	empty	1
30	Central	24	empty	1
31	Central	24	empty	2
32	Marabaixo	16	full	3
33	Marabaixo	16	full	1
34	Marabaixo	16	empty	1
35	Marabaixo	16	empty	1
36	Central	14	full	1
37	Marabaixo	46	empty	1
38	Marabaixo	32	empty	2
39	Central	32	empty	1
40	Central	32	empty	1
41	Marabaixo	28	full	1
42	Central	28	empty	1
43	Central	28	empty	1
44	Central	28	empty	1
45	Marabaixo	28	empty	1
46	Central	28	full	1
47	Marabaixo	28	full	1
48	Central	36	full	1
49	Central	52	full	1
50	Central	52	full	1
51	Central	26	empty	2
52	Central	26	empty	5
53	Central	22	empty	2
54	Central	22	full	1
55	Central	22	empty	1
56	Central	22	empty	2
57	Marabaixo	22	empty	2
58	Central	22	full	1
59	Marabaixo	22	empty	1
60	Marabaixo	22	full	1
